# Supplementary figures and images for: Histone methyltransferase activity affects metabolism in human cells independently of transcriptional regulation
Source: PLoS Biol. 2023 Oct 26;21(10):e3002354. doi: 10.1371/journal.pbio.3002354 (PMC10602318; doi:10.1371/journal.pbio.3002354)

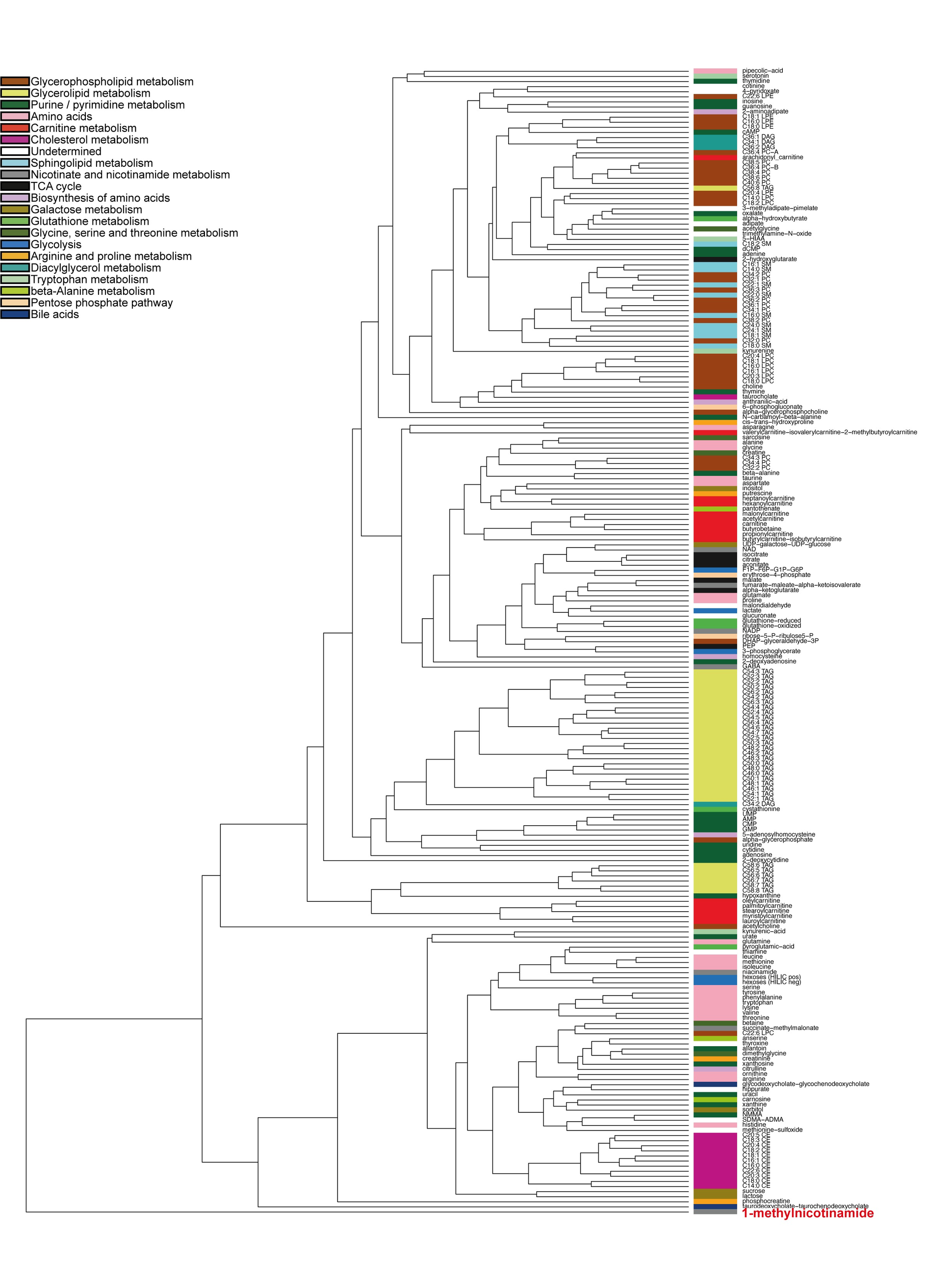

Supplement: S1 Fig — (TIF) [file pbio.3002354.s001.tif]

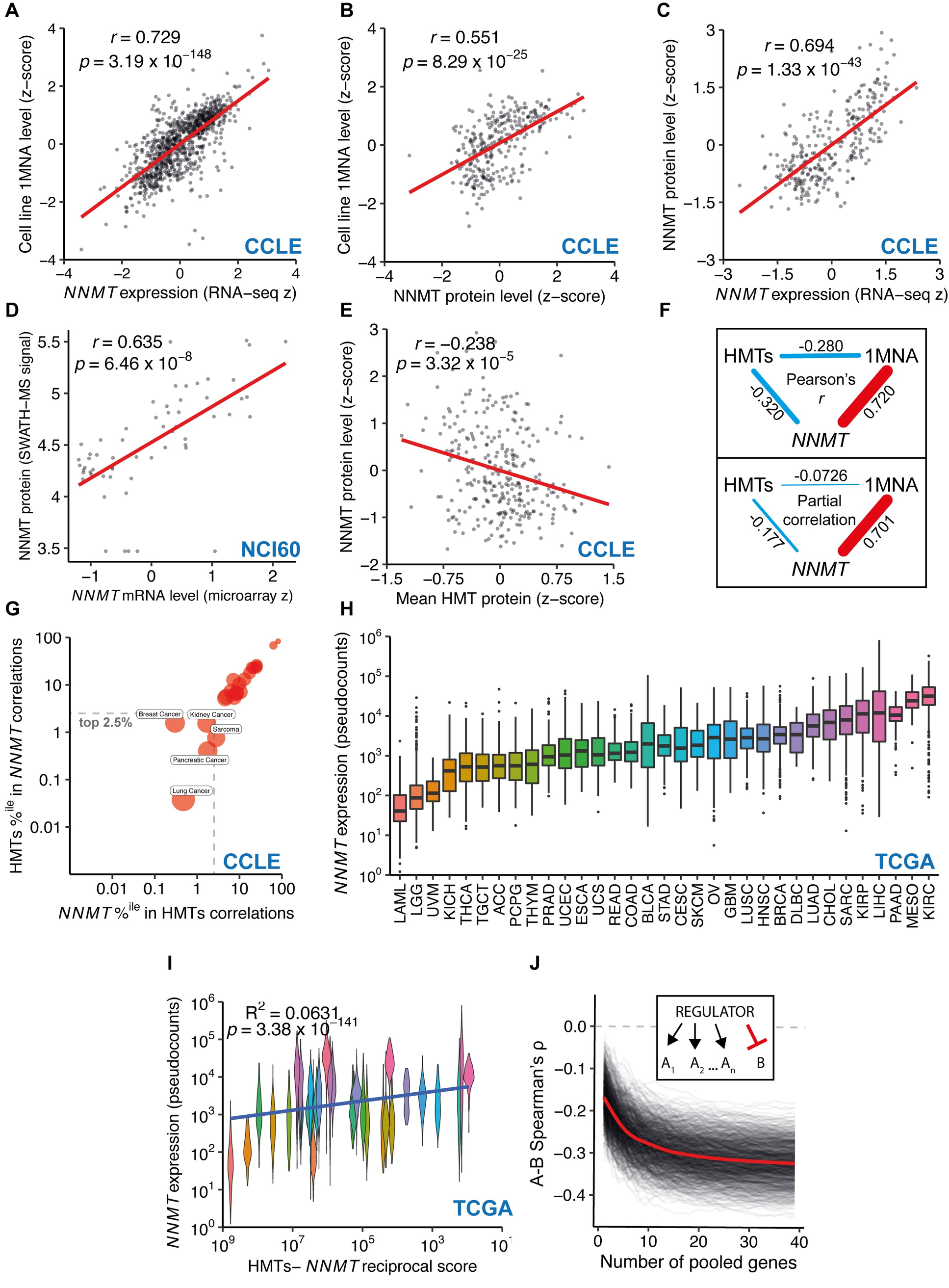

Supplement: S2 Fig — (TIF) [file pbio.3002354.s002.tif]

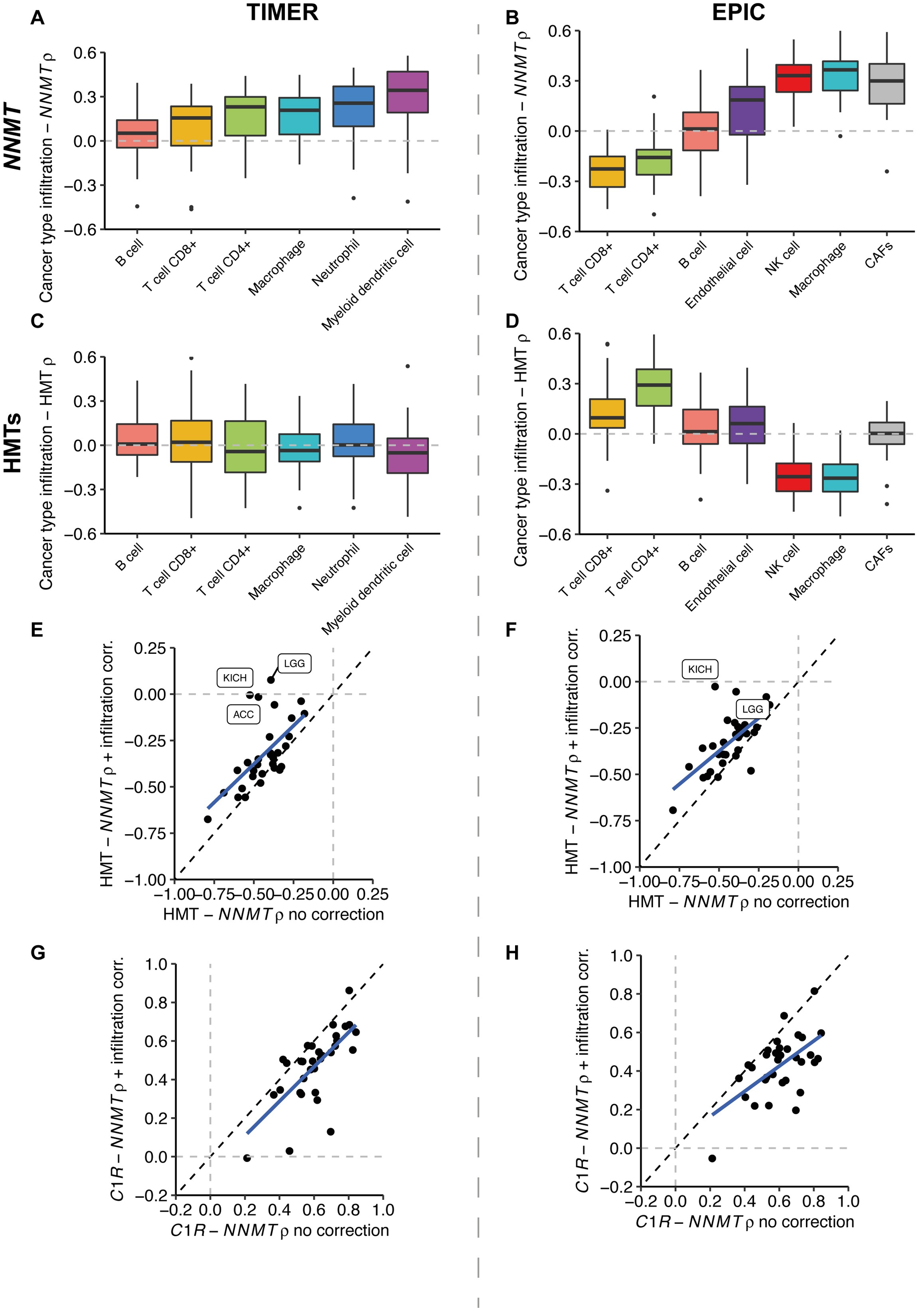

Supplement: S3 Fig — (TIF) [file pbio.3002354.s003.tif]

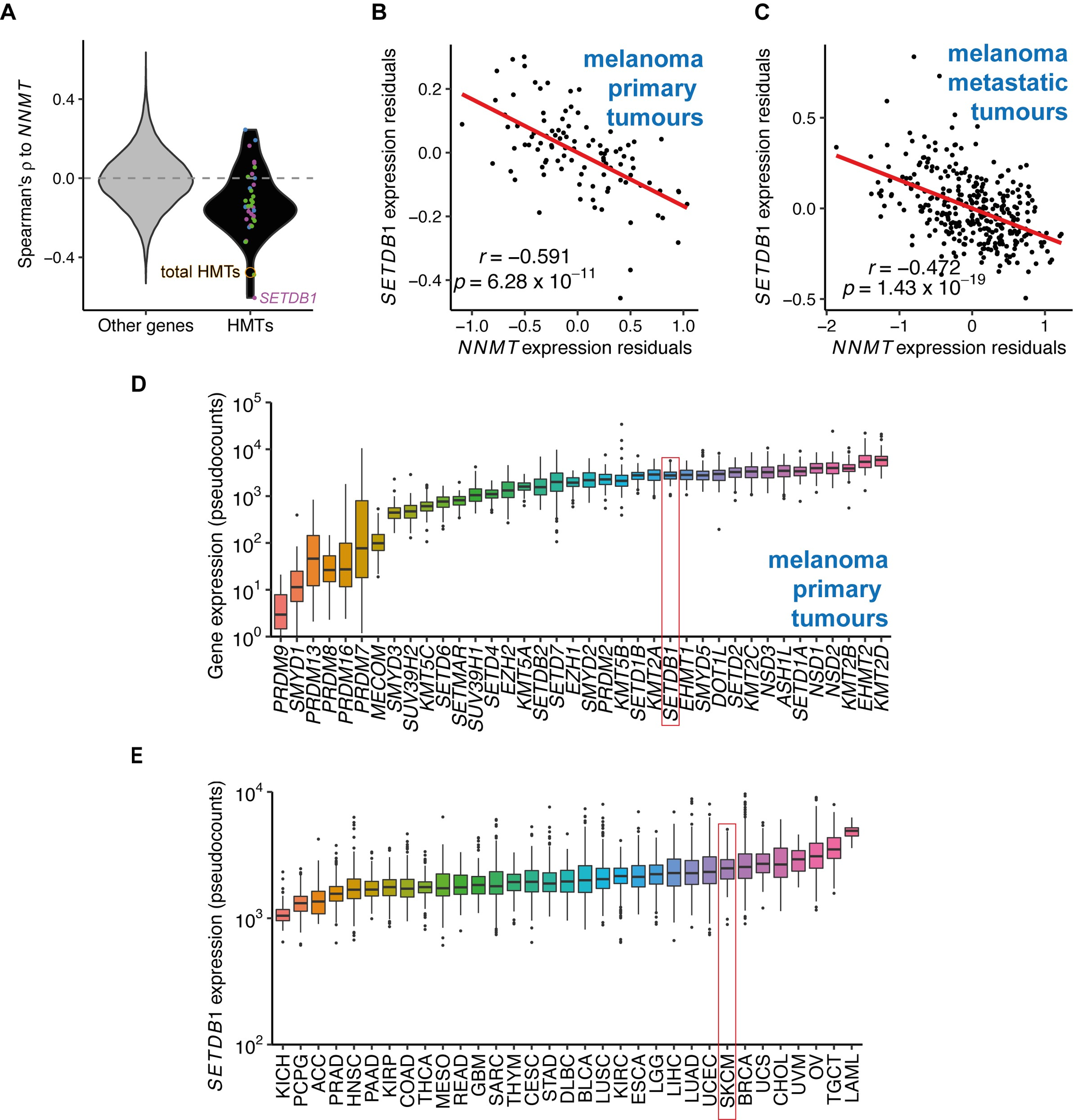

Supplement: S4 Fig — (TIF) [file pbio.3002354.s004.tif]

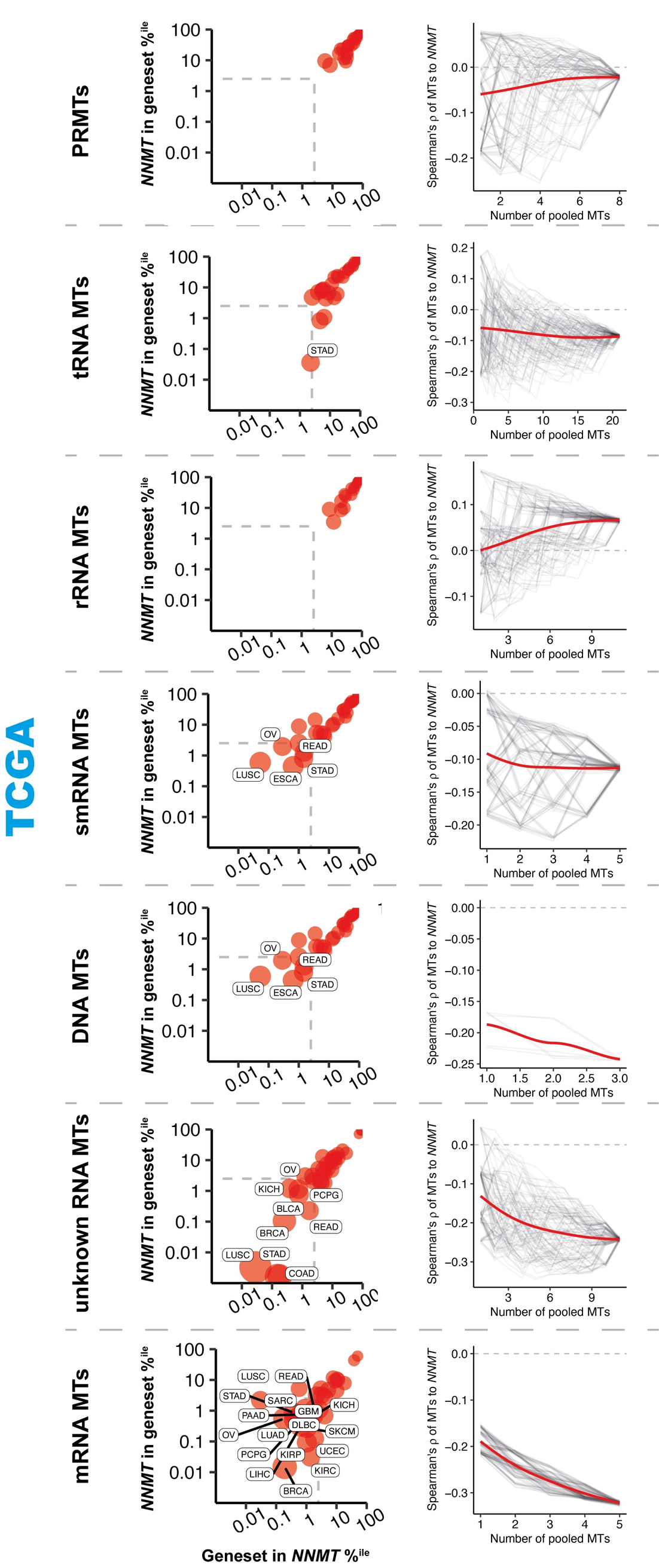

Supplement: S5 Fig — (TIF) [file pbio.3002354.s005.tif]

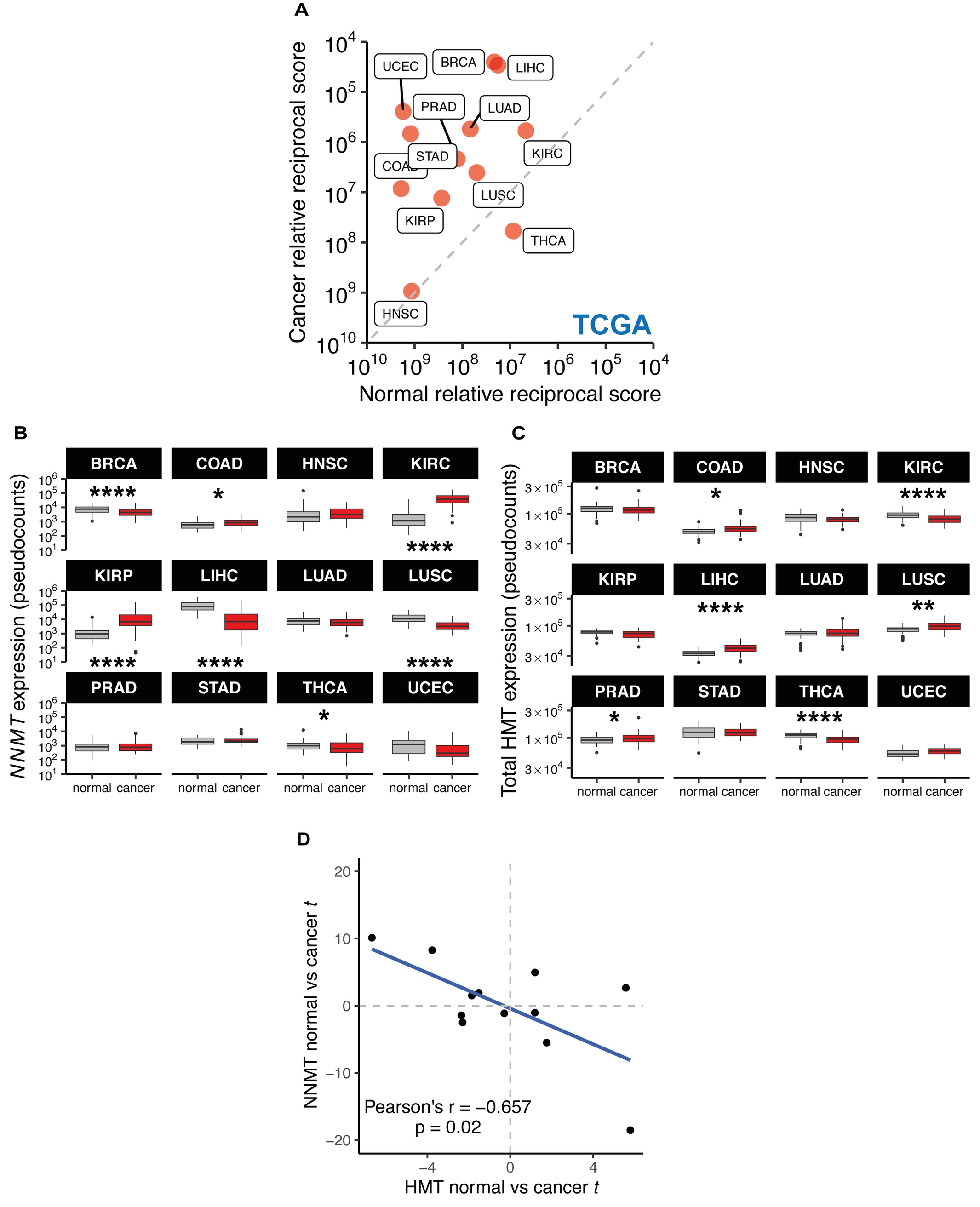

Supplement: S6 Fig — (TIF) [file pbio.3002354.s006.tif]

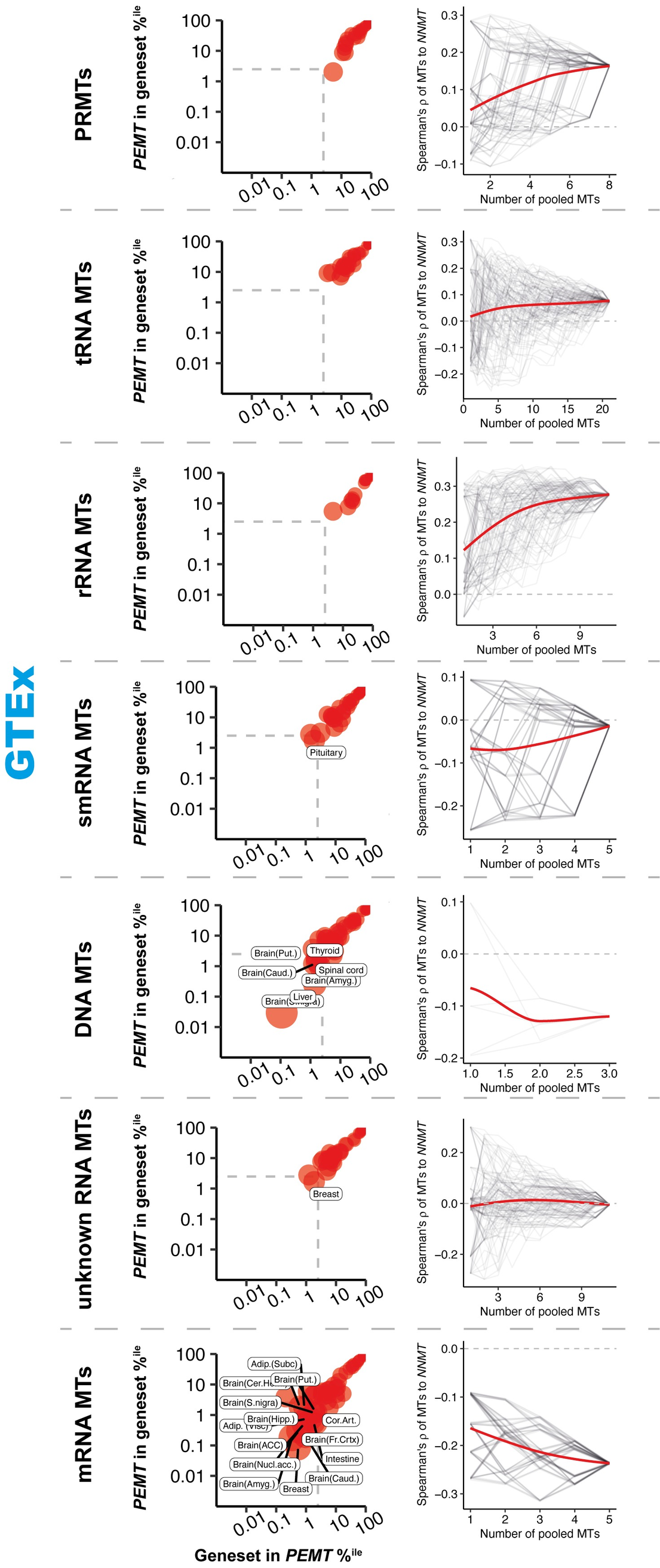

Supplement: S7 Fig — (TIF) [file pbio.3002354.s007.tif]

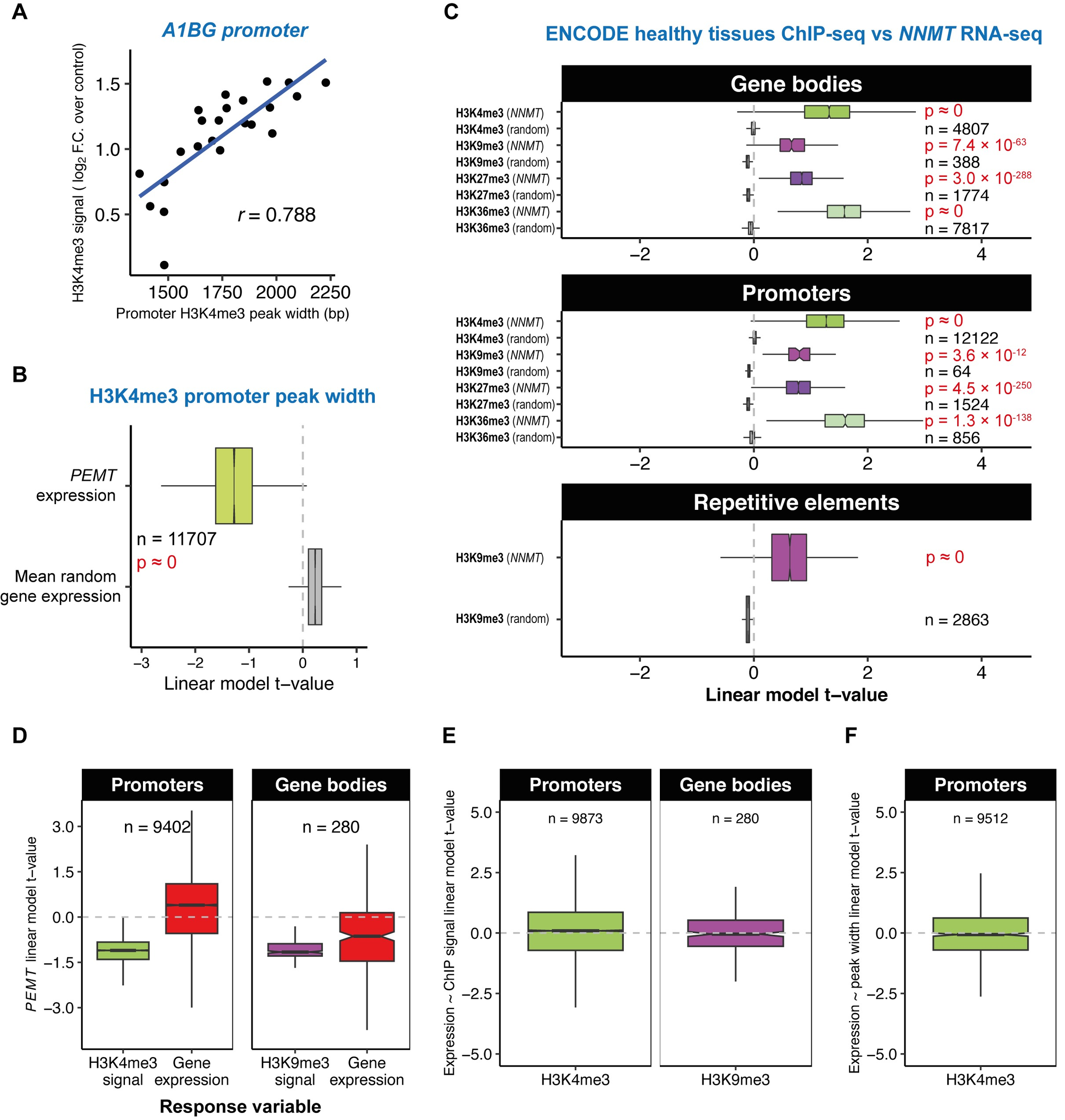

Supplement: S8 Fig — (TIF) [file pbio.3002354.s008.tif]

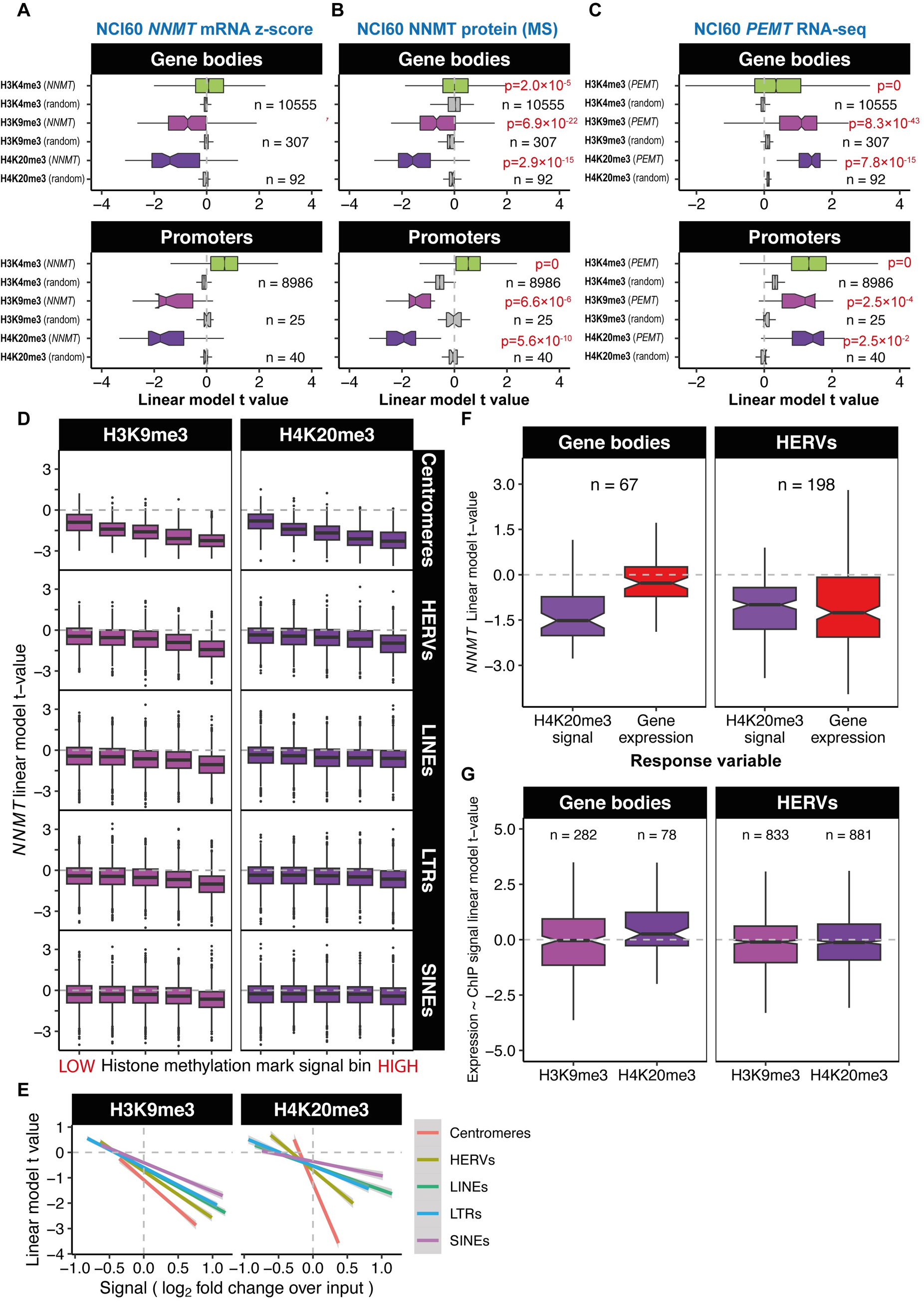

Supplement: S9 Fig — (TIF) [file pbio.3002354.s009.tif]

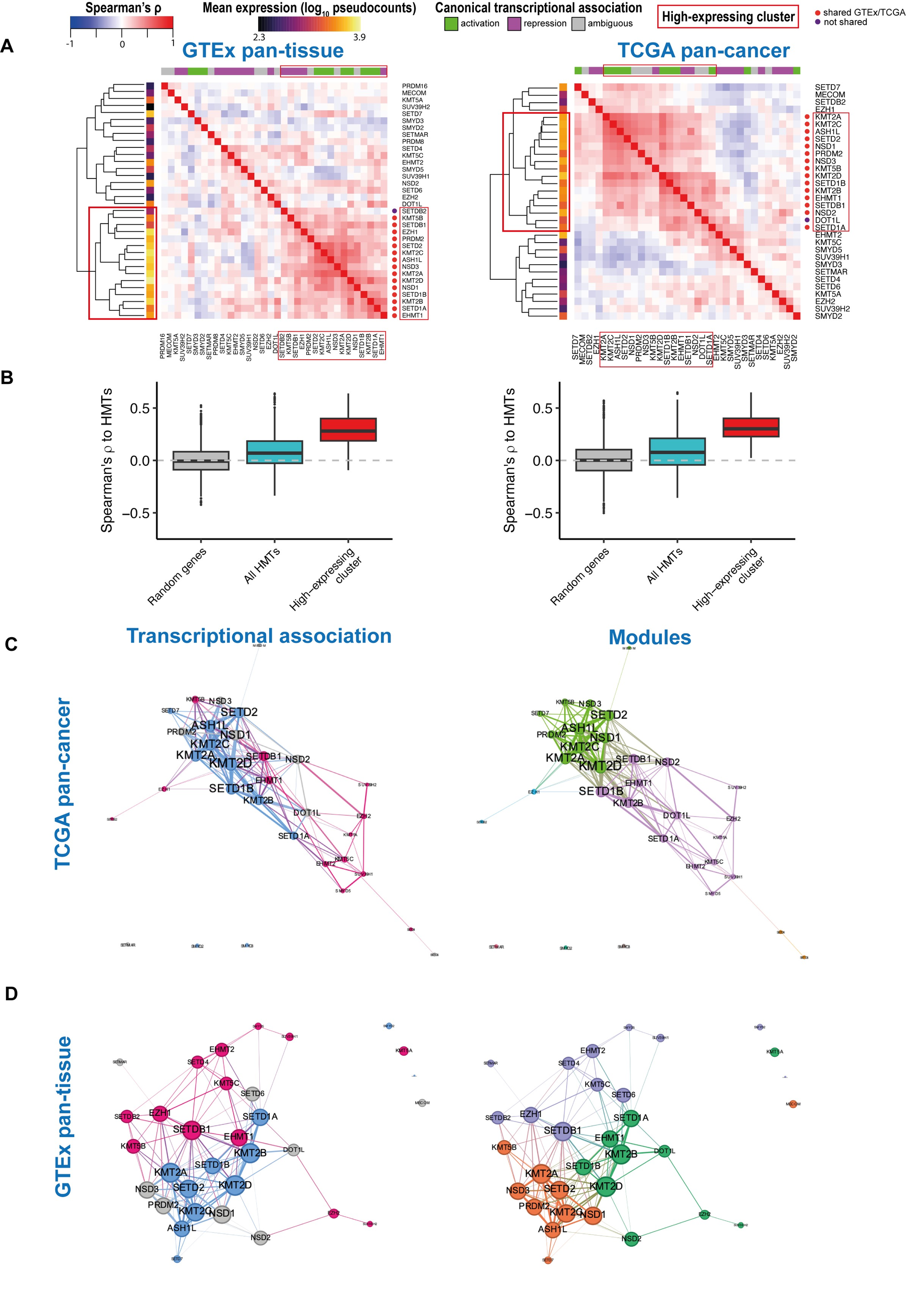

Supplement: S10 Fig — (TIF) [file pbio.3002354.s010.tif]

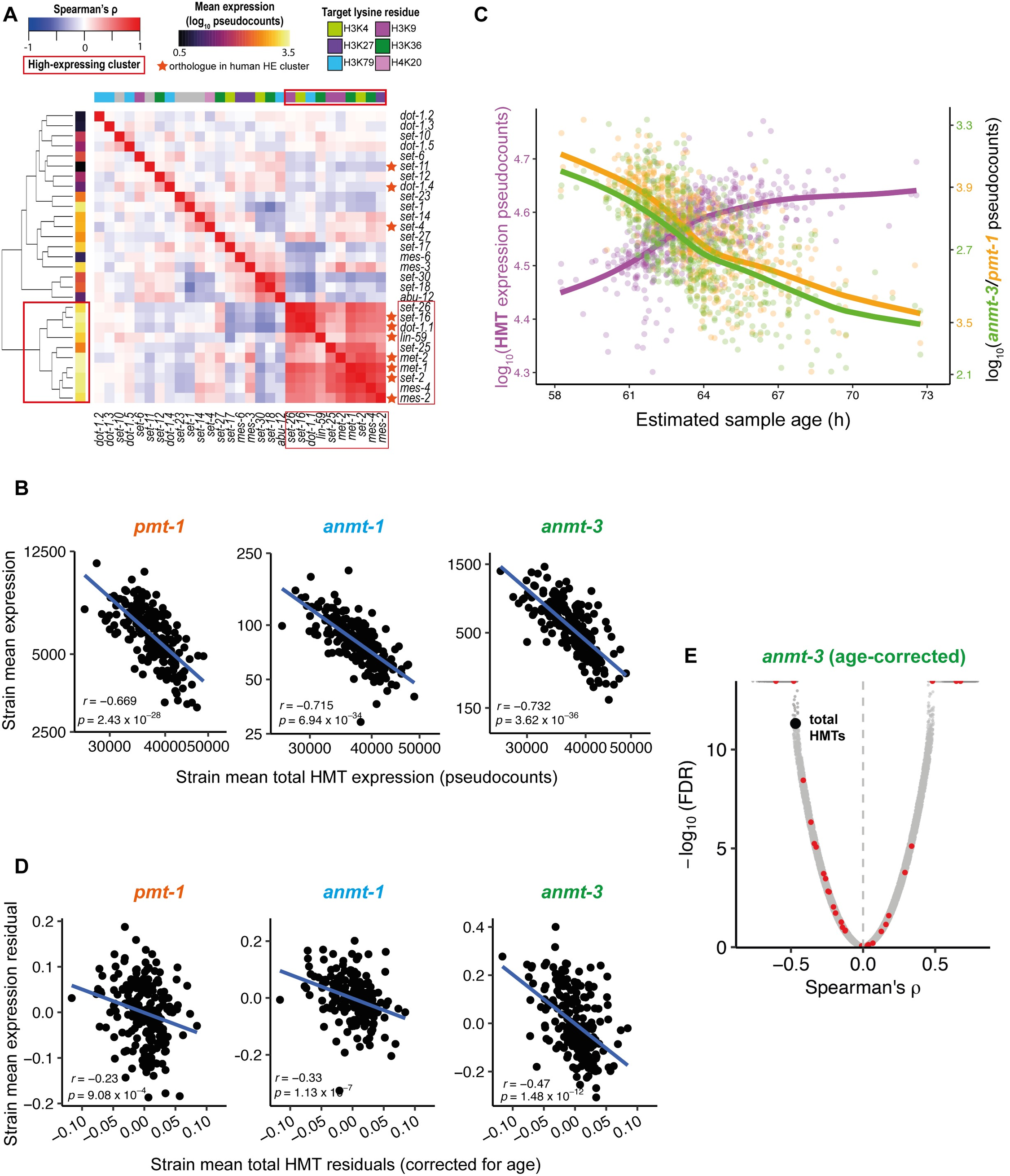

Supplement: S11 Fig — (TIF) [file pbio.3002354.s011.tif]

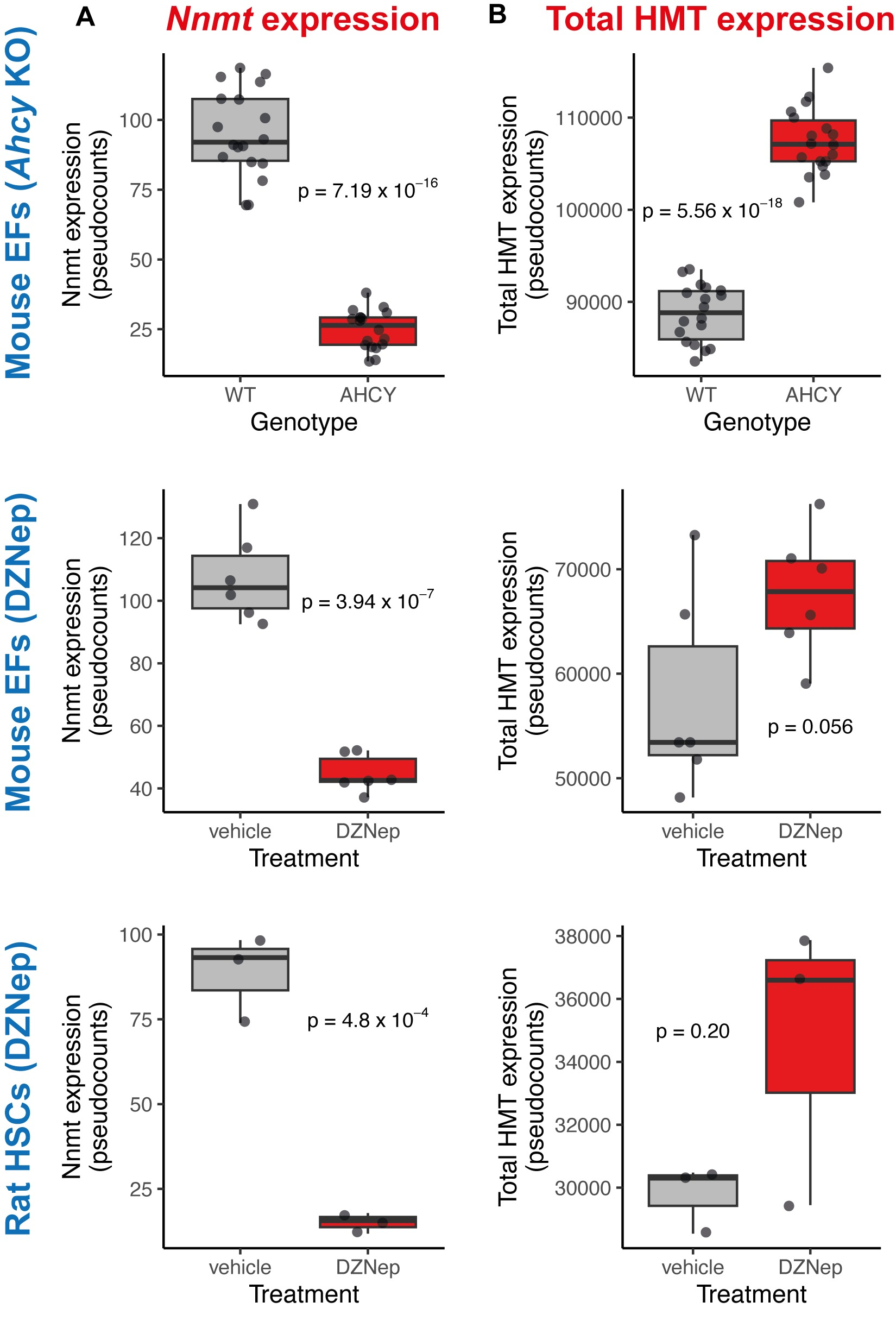

Supplement: S12 Fig — (TIF) [file pbio.3002354.s012.tif]

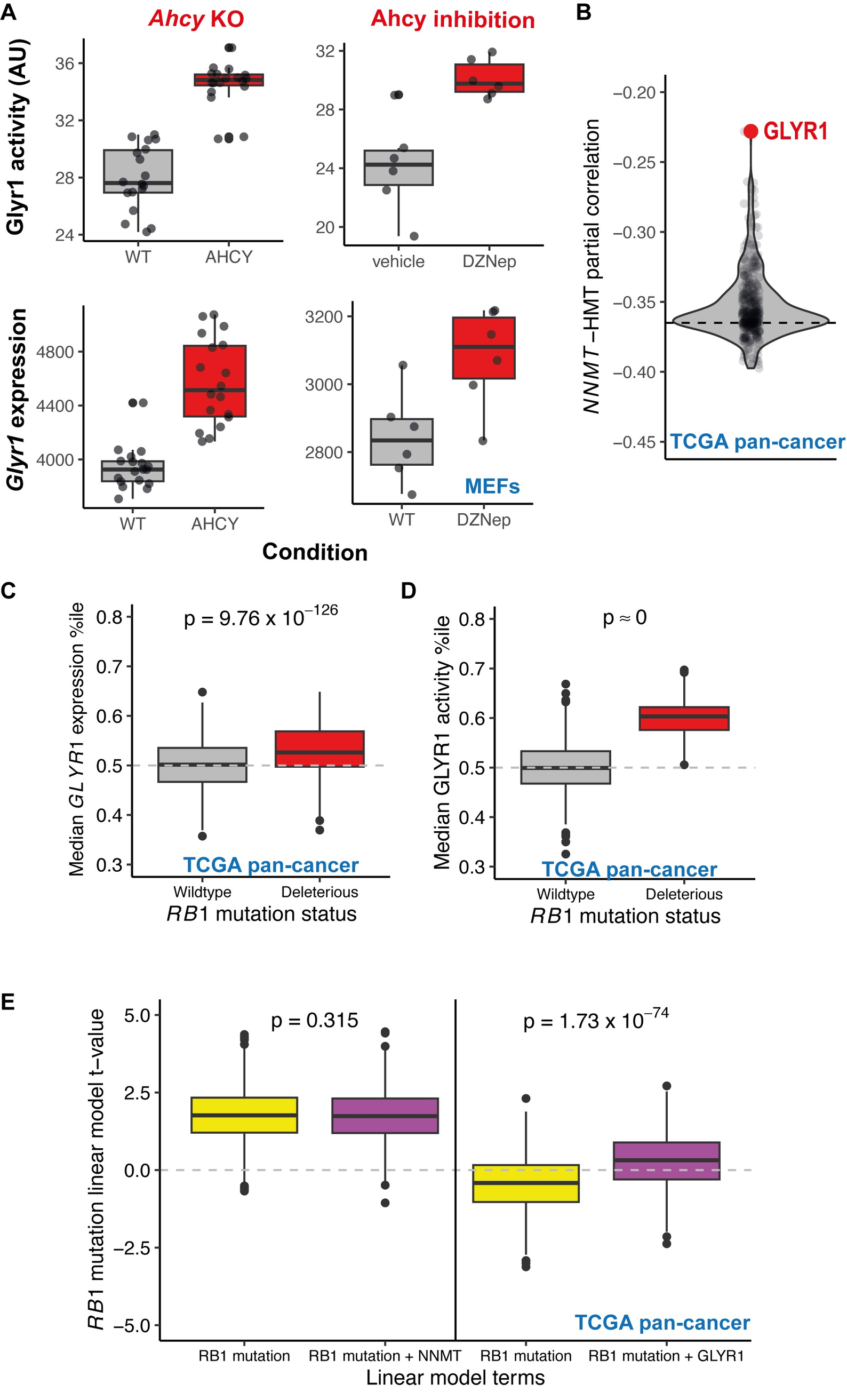

Supplement: S13 Fig — (TIF) [file pbio.3002354.s013.tif]
