# Supplementary material for: Histone methyltransferase activity affects metabolism in human cells independently of transcriptional regulation
Source: PLoS Biol. 2023 Oct 26;21(10):e3002354. doi: 10.1371/journal.pbio.3002354 (PMC10602318; doi:10.1371/journal.pbio.3002354)
Supplement: S5 File — (PDF) [file pbio.3002354.s027.pdf]

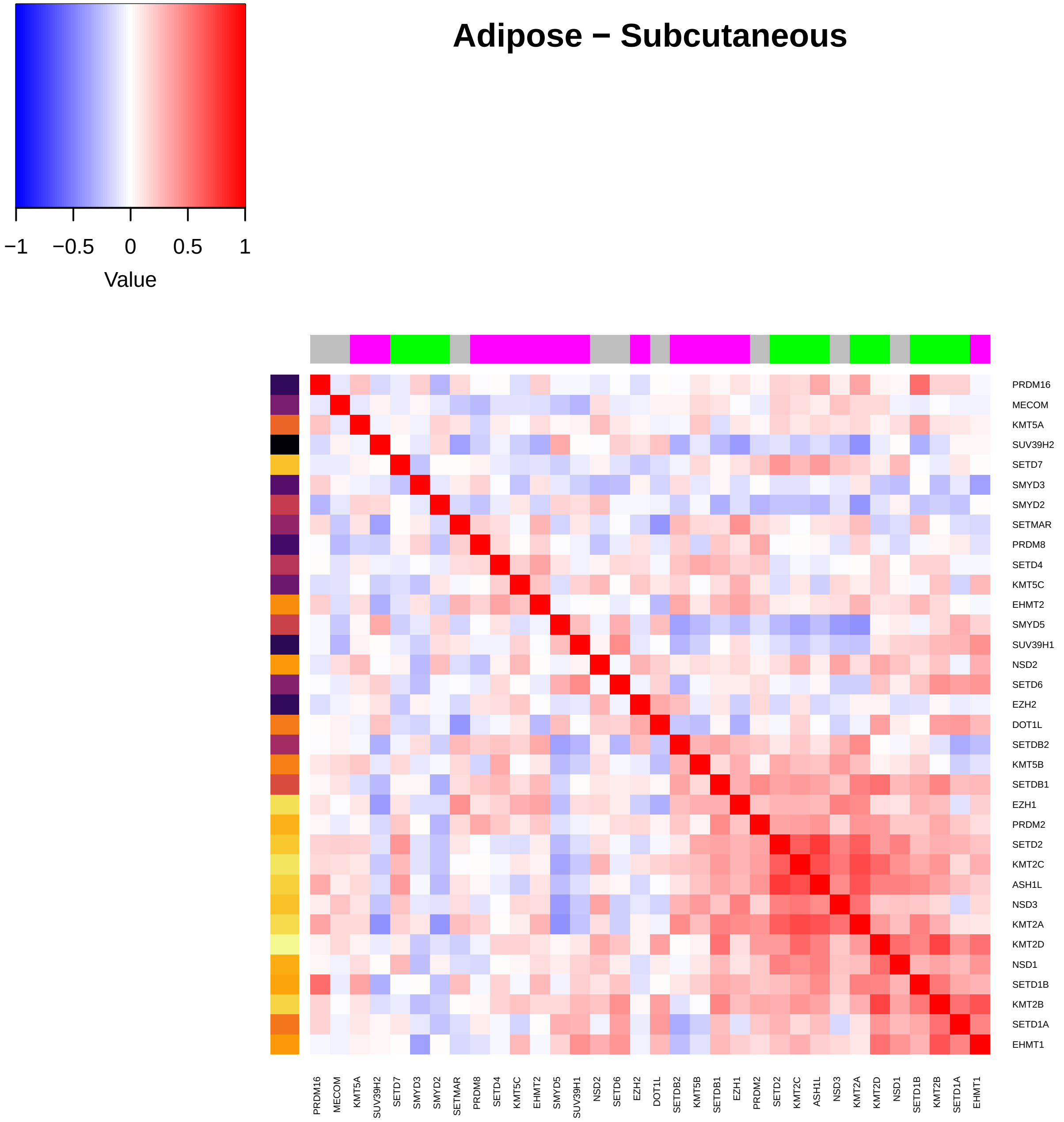
Adipose − Subcutaneous
Value
−1
−0.5
0
0.5
1
PRDM16
MECOM
KMT5A
SUV39H2
SETD7
SMYD3
SMYD2
SETMAR
PRDM8
SETD4
KMT5C
EHMT2
SMYD5
SUV39H1
NSD2
SETD6
EZH2
DOT1L
SETDB2
KMT5B
SETDB1
EZH1
PRDM2
SETD2
KMT2C
ASH1L
NSD3
KMT2A
KMT2D
NSD1
SETD1B
KMT2B
SETD1A
EHMT1

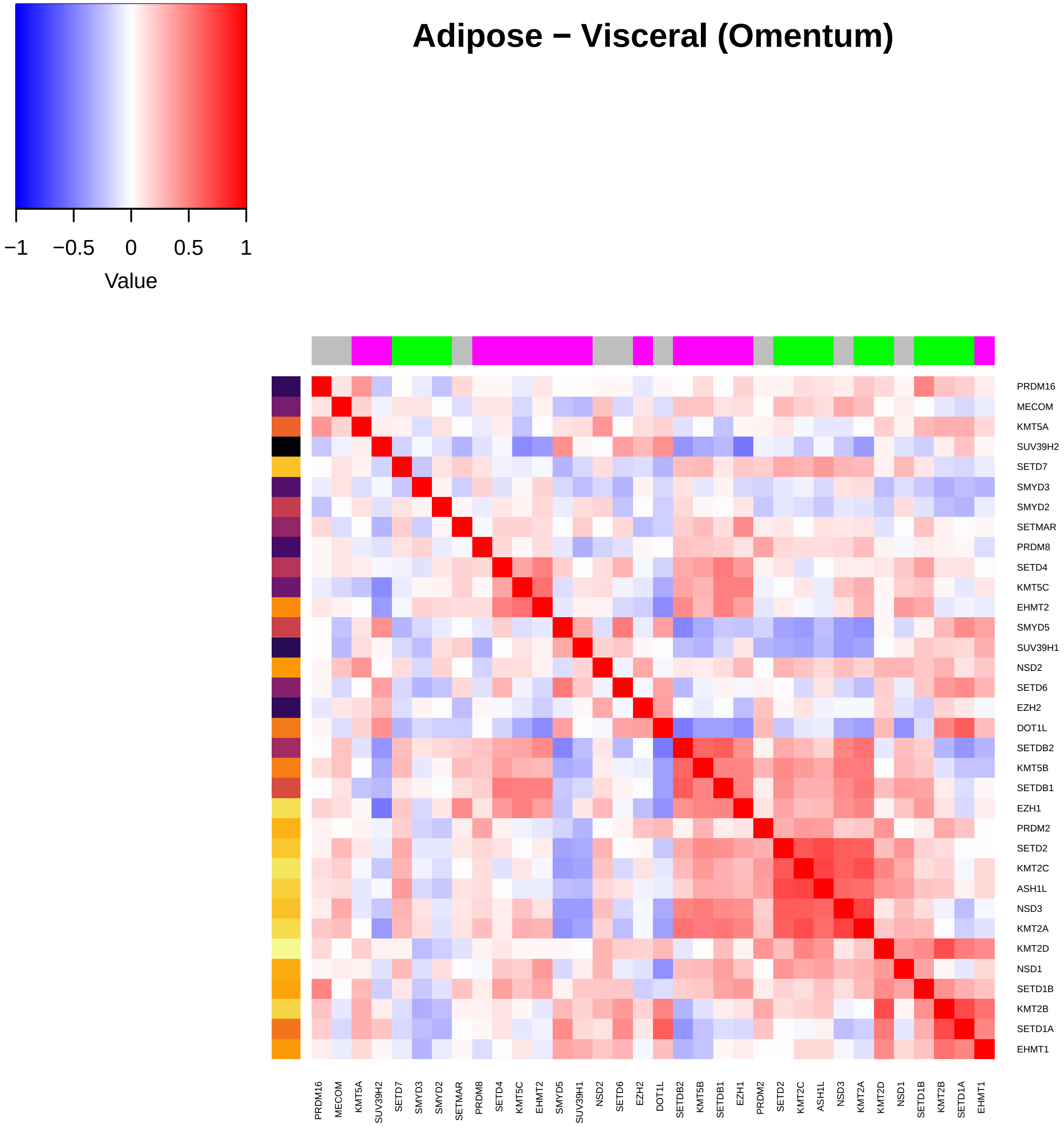
Adipose − Visceral (Omentum)
−1
−0.5
0
0.5
1
Value
PRDM16
MECOM
KMT5A
SUV39H2
SETD7
SMYD3
SMYD2
SETMAR
PRDM8
SETD4
KMT5C
EHMT2
SMYD5
SUV39H1
NSD2
SETD6
EZH2
DOT1L
SETDB2
KMT5B
SETDB1
EZH1
PRDM2
SETD2
KMT2C
ASH1L
NSD3
KMT2A
KMT2D
NSD1
SETD1B
KMT2B
SETD1A
EHMT1

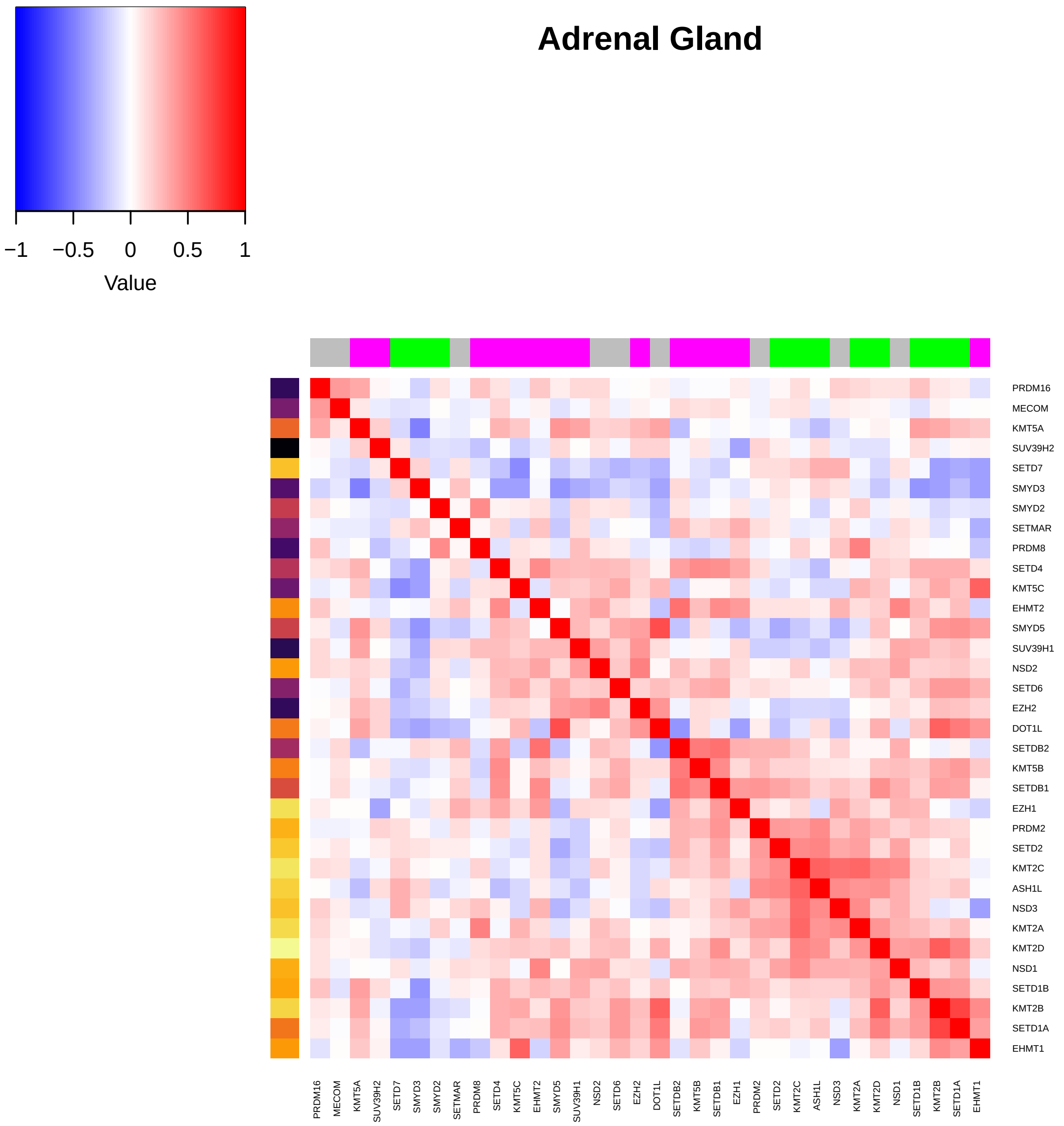

Value
−1
−0.5
0
0.5
1
Adrenal Gland
PRDM16
MECOM
KMT5A
SUV39H2
SETD7
SMYD3
SMYD2
SETMAR
PRDM8
SETD4
KMT5C
EHMT2
SMYD5
SUV39H1
NSD2
SETD6
EZH2
DOT1L
SETDB2
KMT5B
SETDB1
EZH1
PRDM2
SETD2
KMT2C
ASH1L
NSD3
KMT2A
KMT2D
NSD1
SETD1B
KMT2B
SETD1A
EHMT1

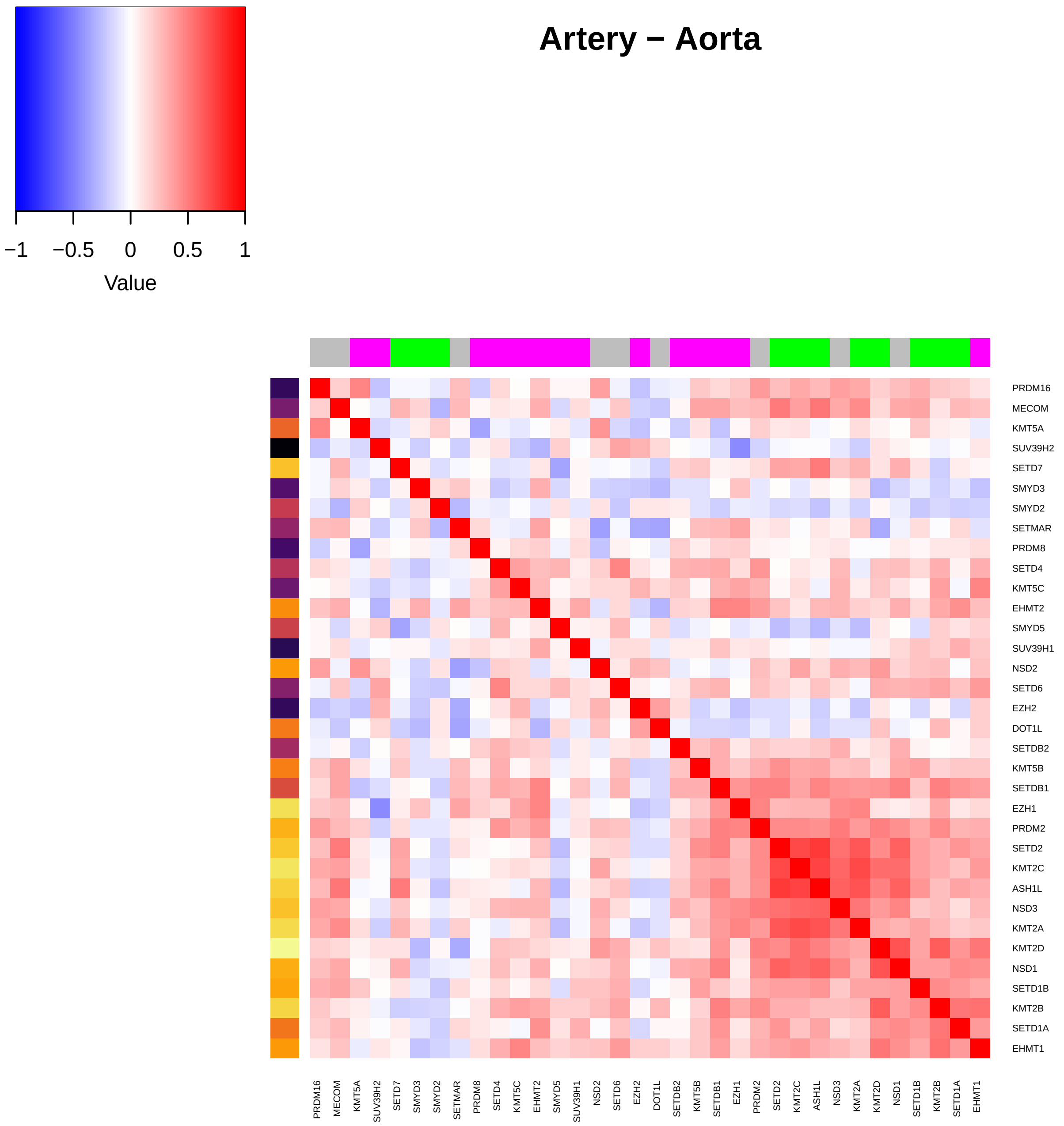

−1
−0.5
0
0.5
1
Value
Artery − Aorta
PRDM16
MECOM
KMT5A
SUV39H2
SETD7
SMYD3
SMYD2
SETMAR
PRDM8
SETD4
KMT5C
EHMT2
SMYD5
SUV39H1
NSD2
SETD6
EZH2
DOT1L
SETDB2
KMT5B
SETDB1
EZH1
PRDM2
SETD2
KMT2C
ASH1L
NSD3
KMT2A
KMT2D
NSD1
SETD1B
KMT2B
SETD1A
EHMT1

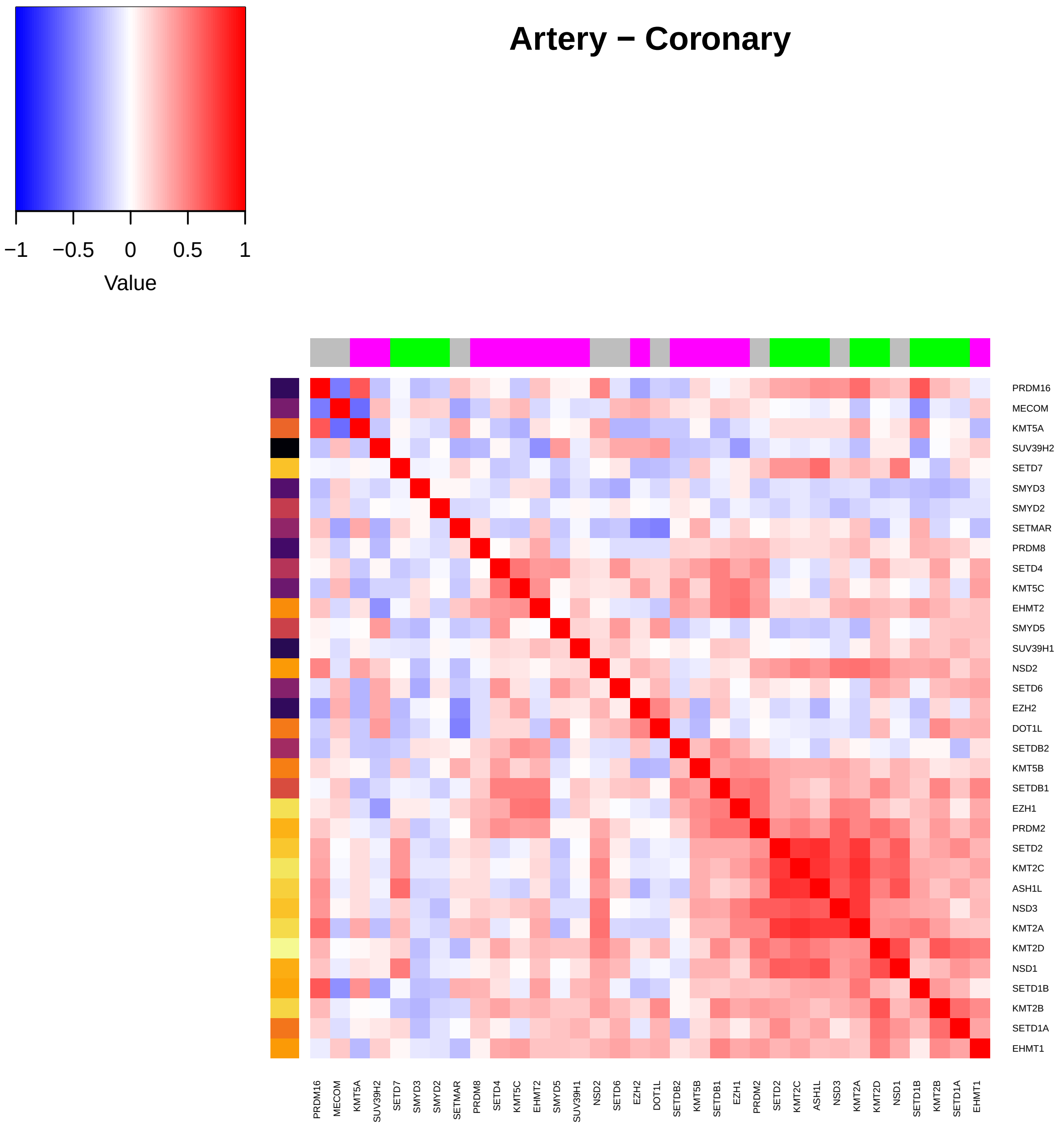
Artery – Coronary
−1
−0.5
0
0.5
1
Value
PRDM16
MECOM
KMT5A
SUV39H2
SETD7
SMYD3
SMYD2
SETMAR
PRDM8
SETD4
KMT5C
EHMT2
SMYD5
SUV39H1
NSD2
SETD6
EZH2
DOT1L
SETDB2
KMT5B
SETDB1
EZH1
PRDM2
SETD2
KMT2C
ASH1L
NSD3
KMT2A
KMT2D
NSD1
SETD1B
KMT2B
SETD1A
EHMT1

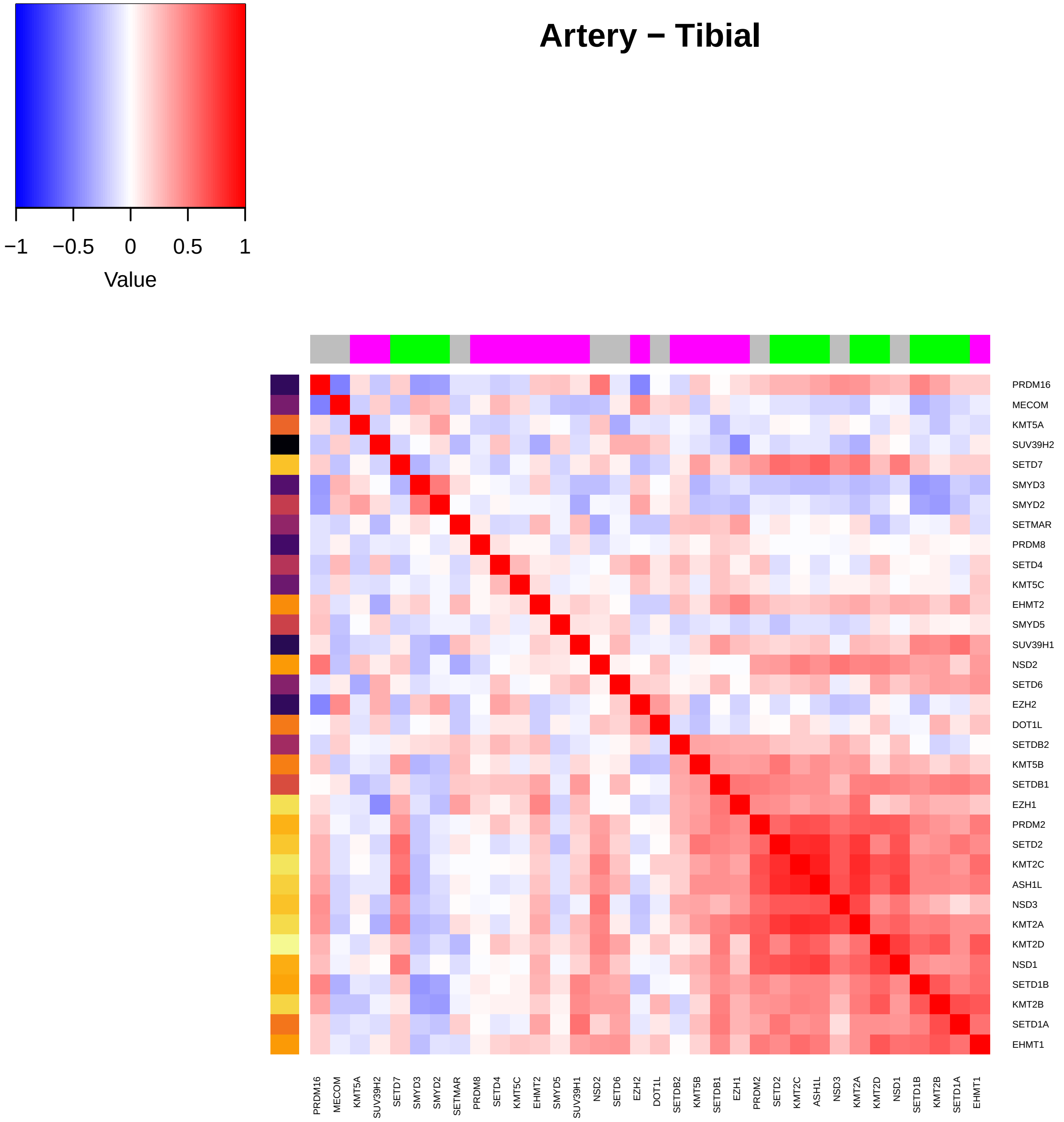
Artery – Tibial
−1
−0.5
0
0.5
1
Value
PRDM16
MECOM
KMT5A
SUV39H2
SETD7
SMYD3
SMYD2
SETMAR
PRDM8
SETD4
KMT5C
EHMT2
SMYD5
SUV39H1
NSD2
SETD6
EZH2
DOT1L
SETDB2
KMT5B
SETDB1
EZH1
PRDM2
SETD2
KMT2C
ASH1L
NSD3
KMT2A
KMT2D
NSD1
SETD1B
KMT2B
SETD1A
EHMT1

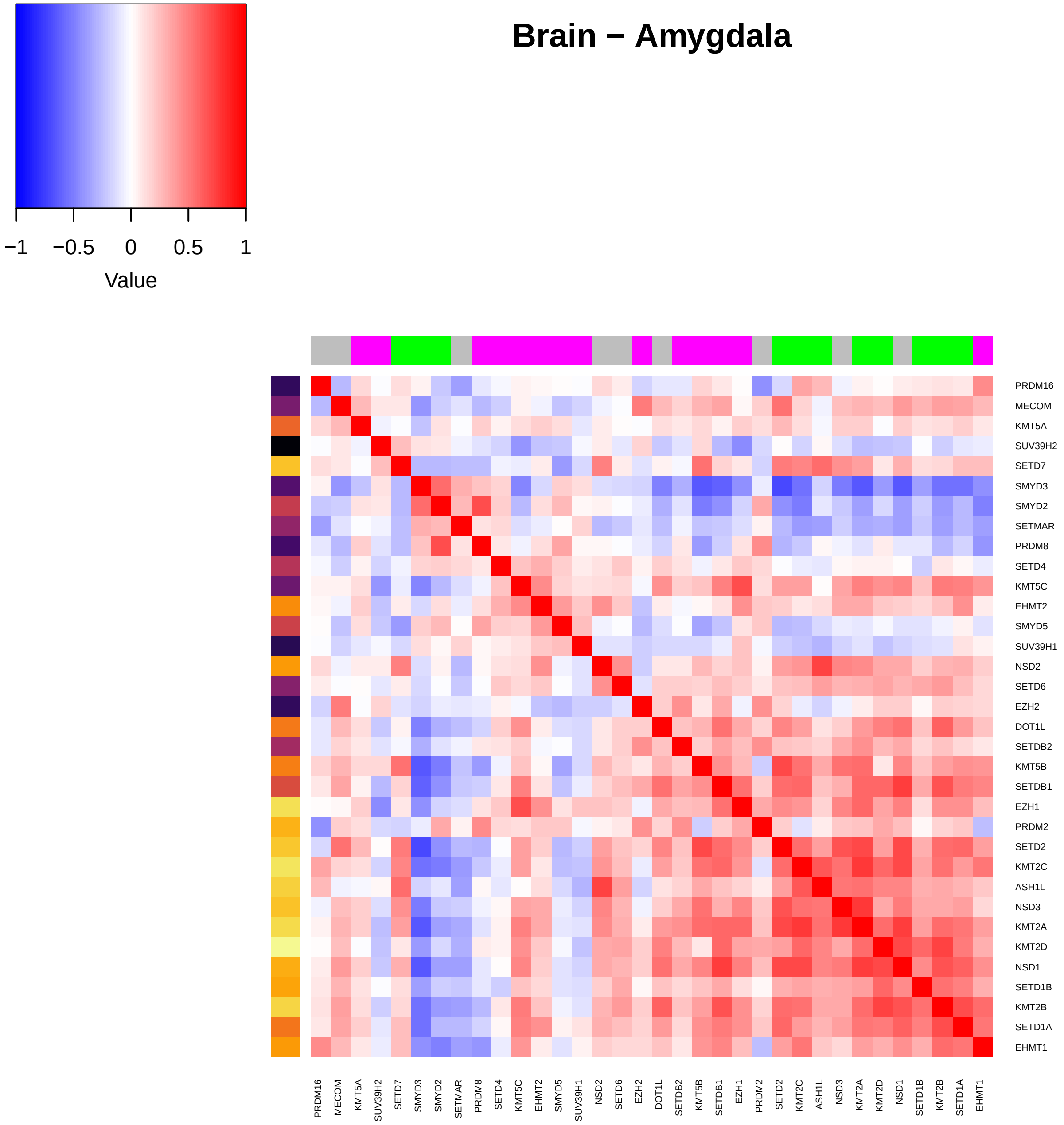
Brain − Amygdala
−1
−0.5
0
0.5
1
Value
PRDM16
MECOM
KMT5A
SUV39H2
SETD7
SMYD3
SMYD2
SETMAR
PRDM8
SETD4
KMT5C
EHMT2
SMYD5
SUV39H1
NSD2
SETD6
EZH2
DOT1L
SETDB2
KMT5B
SETDB1
EZH1
PRDM2
SETD2
KMT2C
ASH1L
NSD3
KMT2A
KMT2D
NSD1
SETD1B
KMT2B
SETD1A
EHMT1

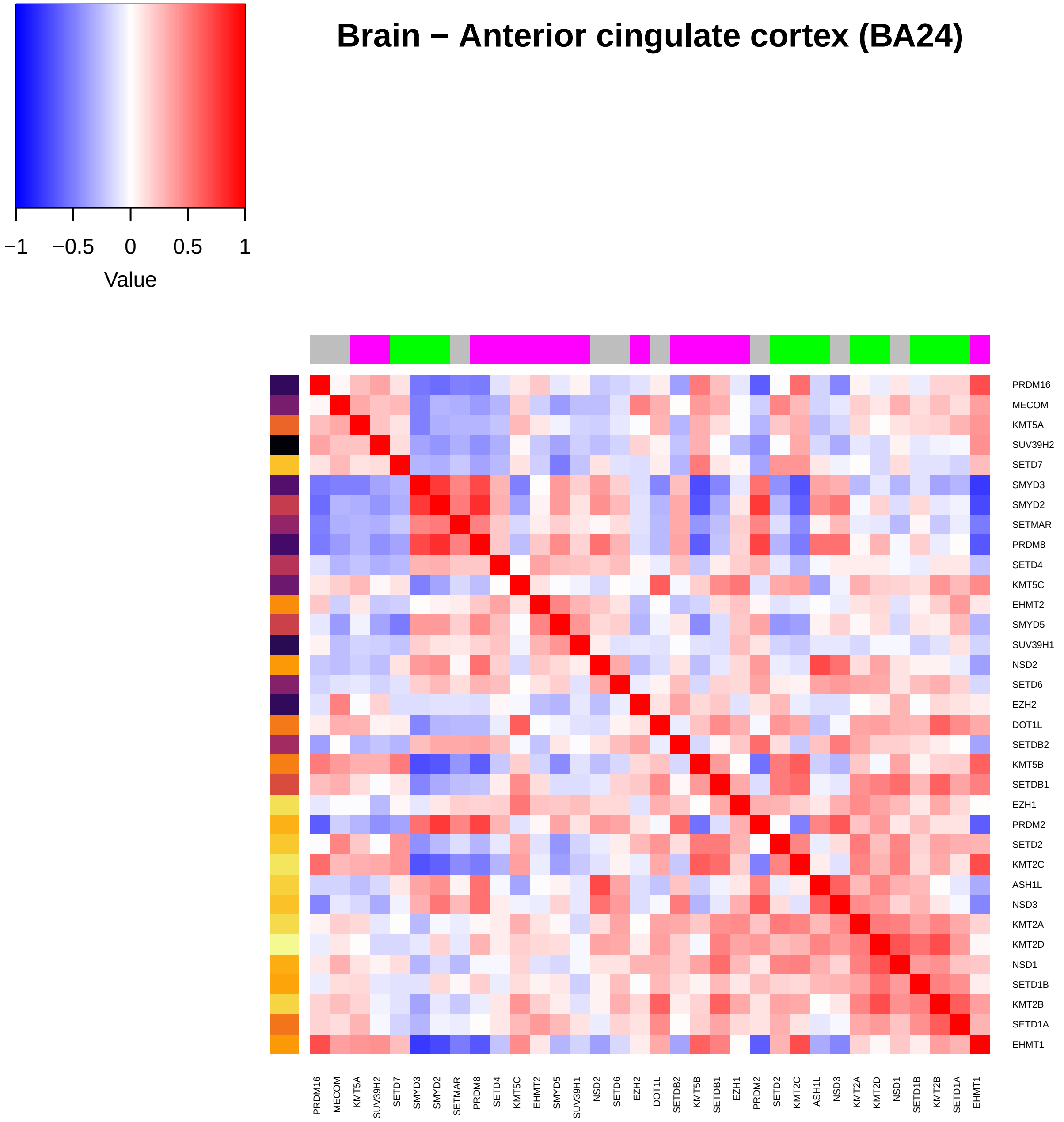
Brain − Anterior cingulate cortex (BA24)
−1
−0.5
0
0.5
1
Value
PRDM16
MECOM
KMT5A
SUV39H2
SETD7
SMYD3
SMYD2
SETMAR
PRDM8
SETD4
KMT5C
EHMT2
SMYD5
SUV39H1
NSD2
SETD6
EZH2
DOT1L
SETDB2
KMT5B
SETDB1
EZH1
PRDM2
SETD2
KMT2C
ASH1L
NSD3
KMT2A
KMT2D
NSD1
SETD1B
KMT2B
SETD1A
EHMT1

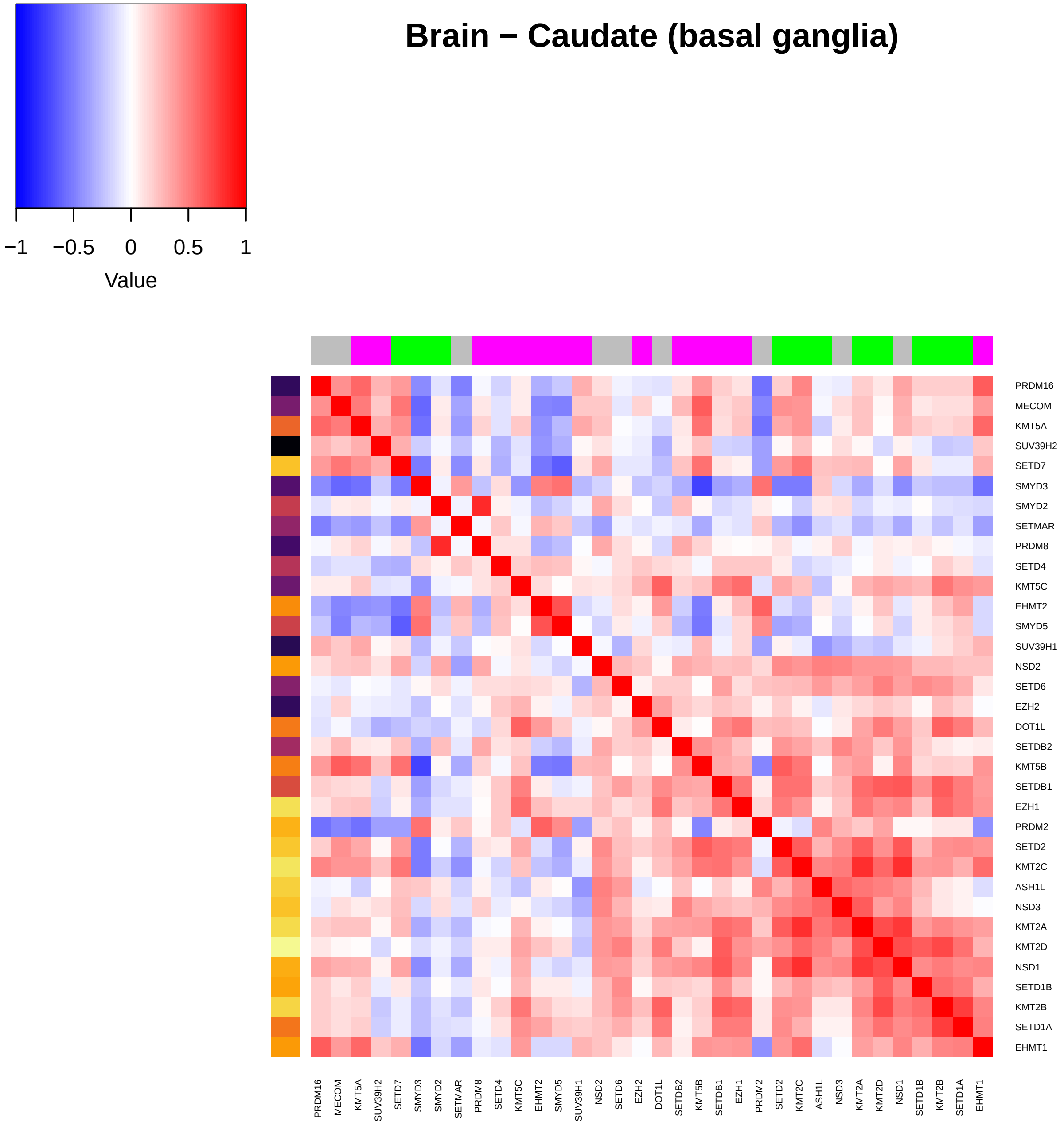
Brain − Caudate (basal ganglia)
−1
−0.5
0
0.5
1
Value
PRDM16
MECOM
KMT5A
SUV39H2
SETD7
SMYD3
SMYD2
SETMAR
PRDM8
SETD4
KMT5C
EHMT2
SMYD5
SUV39H1
NSD2
SETD6
EZH2
DOT1L
SETDB2
KMT5B
SETDB1
EZH1
PRDM2
SETD2
KMT2C
ASH1L
NSD3
KMT2A
KMT2D
NSD1
SETD1B
KMT2B
SETD1A
EHMT1

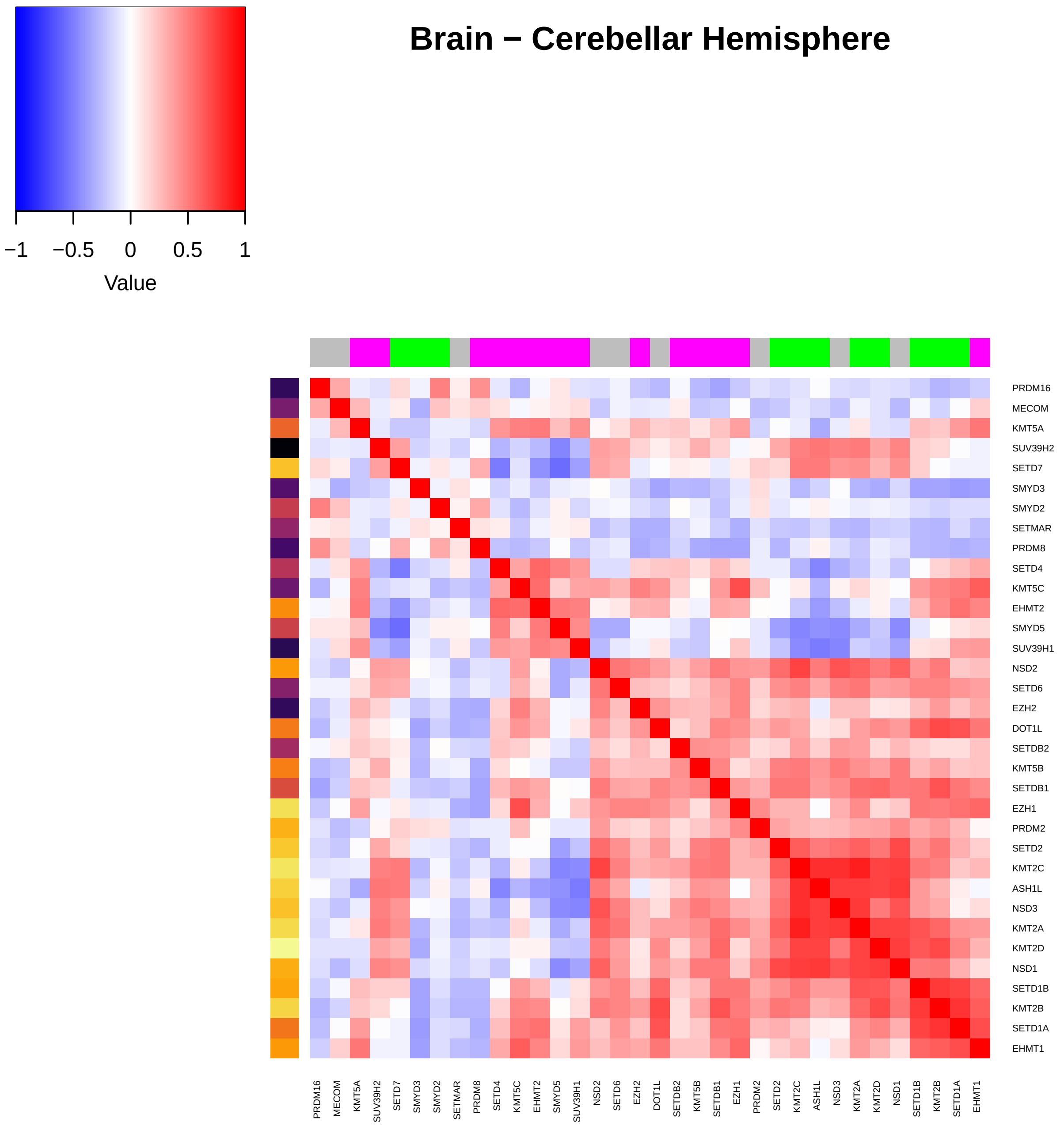

Brain − Cerebellar Hemisphere
−1
−0.5
0
0.5
1
Value
PRDM16
MECOM
KMT5A
SUV39H2
SETD7
SMYD3
SMYD2
SETMAR
PRDM8
SETD4
KMT5C
EHMT2
SMYD5
SUV39H1
NSD2
SETD6
EZH2
DOT1L
SETDB2
KMT5B
SETDB1
EZH1
PRDM2
SETD2
KMT2C
ASH1L
NSD3
KMT2A
KMT2D
NSD1
SETD1B
KMT2B
SETD1A
EHMT1

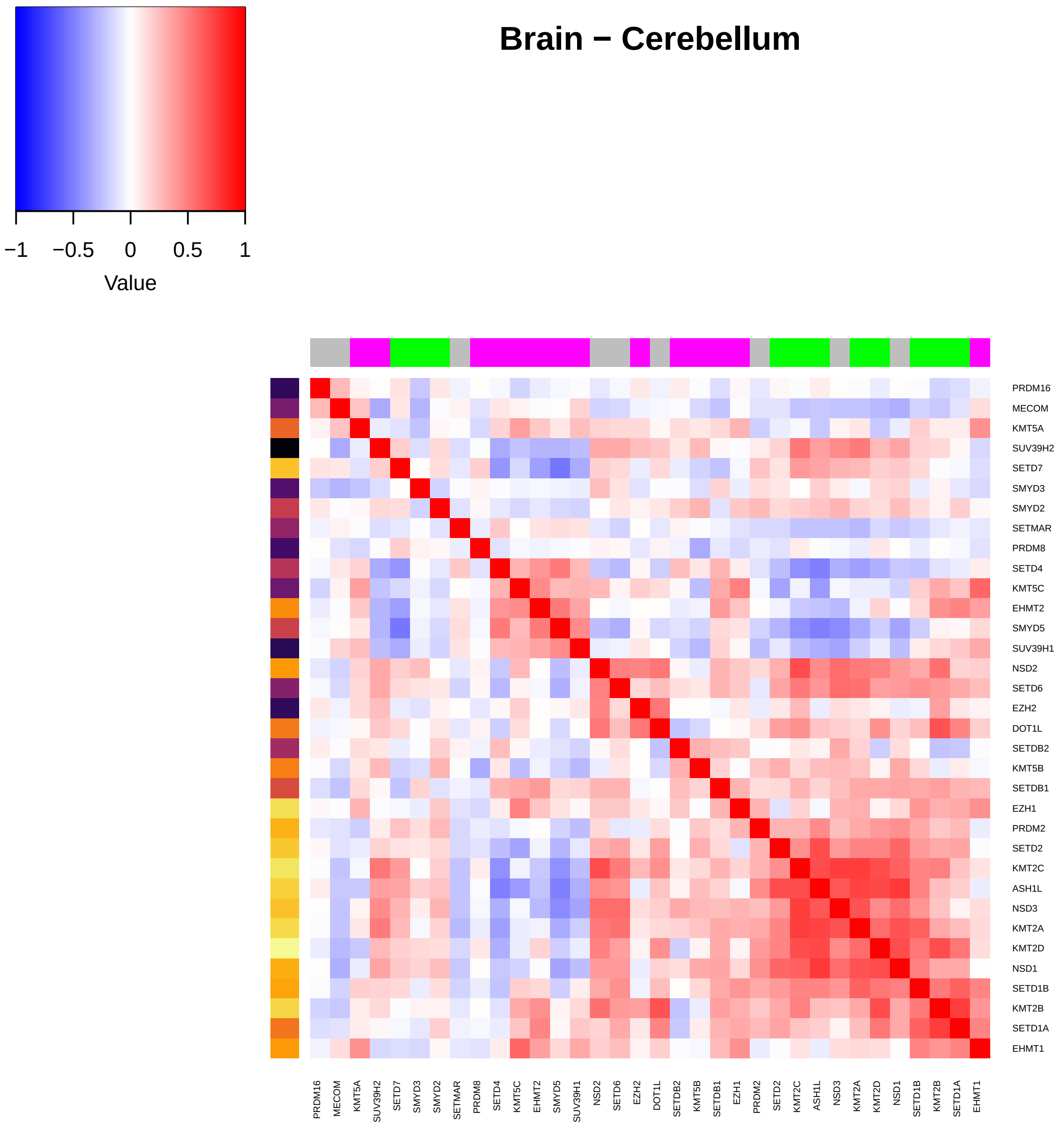
Brain − Cerebellum
−1
−0.5
0
0.5
1
Value
PRDM16
MECOM
KMT5A
SUV39H2
SETD7
SMYD3
SMYD2
SETMAR
PRDM8
SETD4
KMT5C
EHMT2
SMYD5
SUV39H1
NSD2
SETD6
EZH2
DOT1L
SETDB2
KMT5B
SETDB1
EZH1
PRDM2
SETD2
KMT2C
ASH1L
NSD3
KMT2A
KMT2D
NSD1
SETD1B
KMT2B
SETD1A
EHMT1

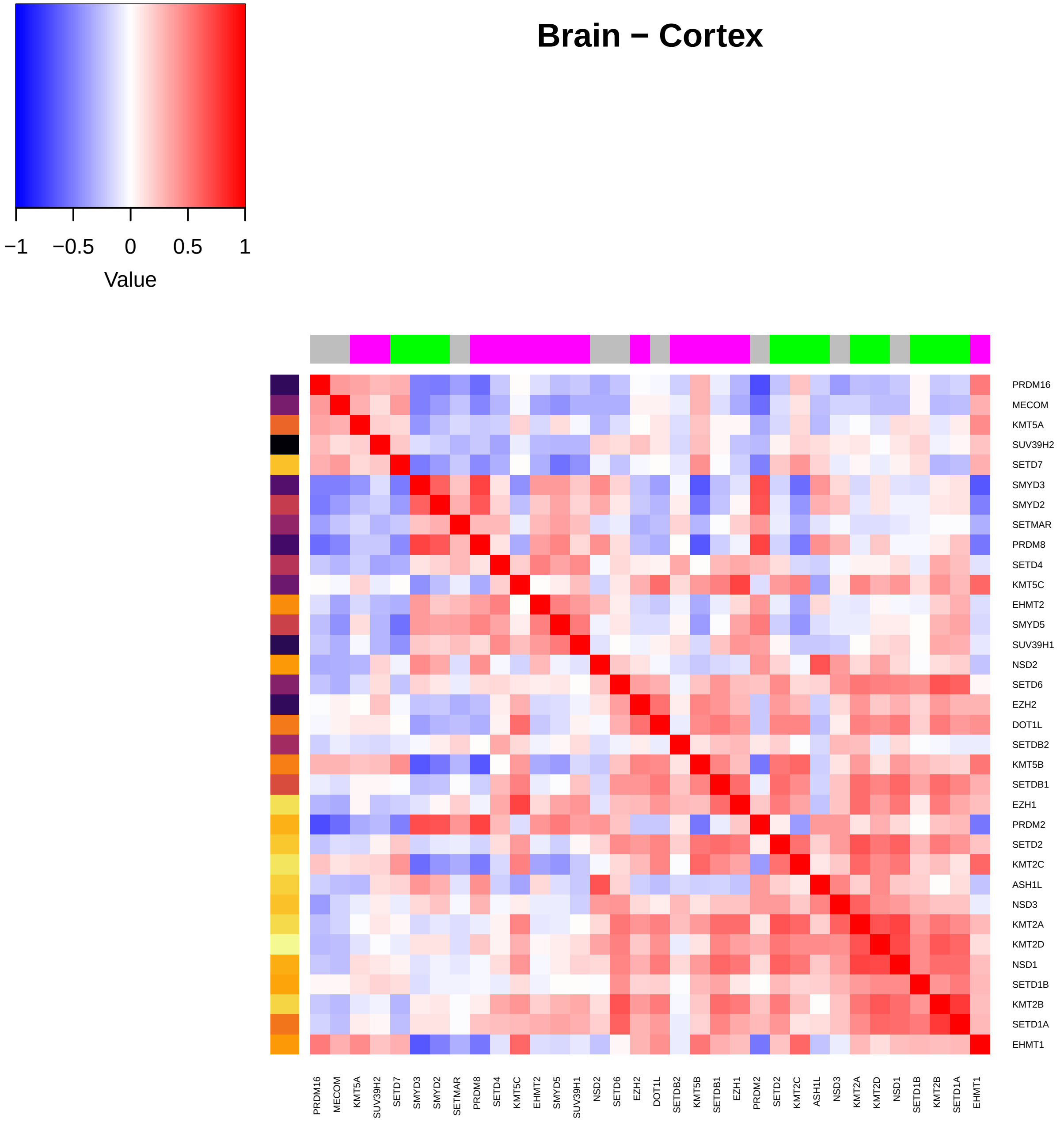

Brain − Cortex
−1
−0.5
0
0.5
1
Value
PRDM16
MECOM
KMT5A
SUV39H2
SETD7
SMYD3
SMYD2
SETMAR
PRDM8
SETD4
KMT5C
EHMT2
SMYD5
SUV39H1
NSD2
SETD6
EZH2
DOT1L
SETDB2
KMT5B
SETDB1
EZH1
PRDM2
SETD2
KMT2C
ASH1L
NSD3
KMT2A
KMT2D
NSD1
SETD1B
KMT2B
SETD1A
EHMT1

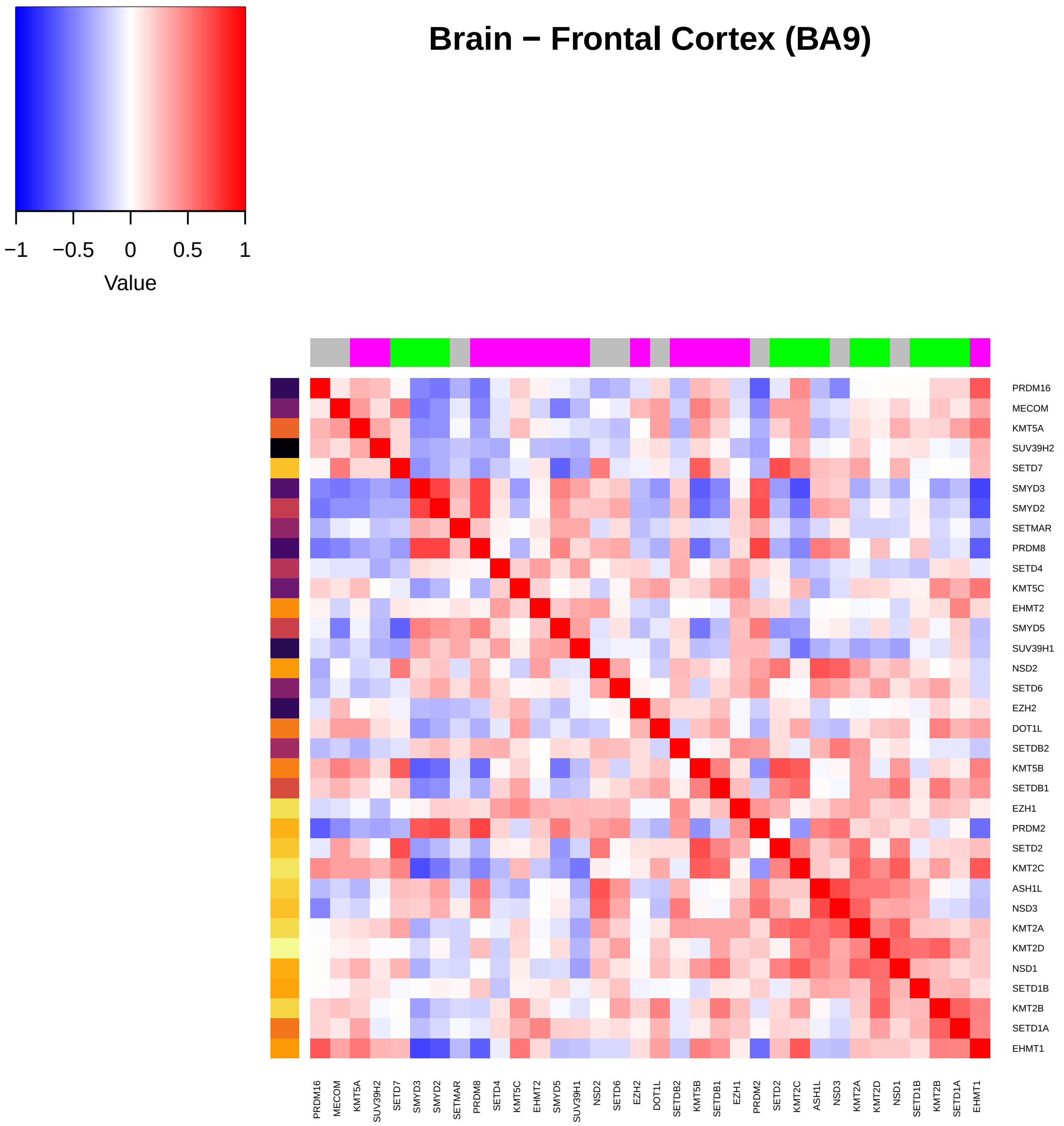
Brain – Frontal Cortex (BA9)
−1
−0.5
0
0.5
1
Value
PRDM16
MECOM
KMT5A
SUV39H2
SETD7
SMYD3
SMYD2
SETMAR
PRDM8
SETD4
KMT5C
EHMT2
SMYD5
SUV39H1
NSD2
SETD6
EZH2
DOT1L
SETDB2
KMT5B
SETDB1
EZH1
PRDM2
SETD2
KMT2C
ASH1L
NSD3
KMT2A
KMT2D
NSD1
SETD1B
KMT2B
SETD1A
EHMT1

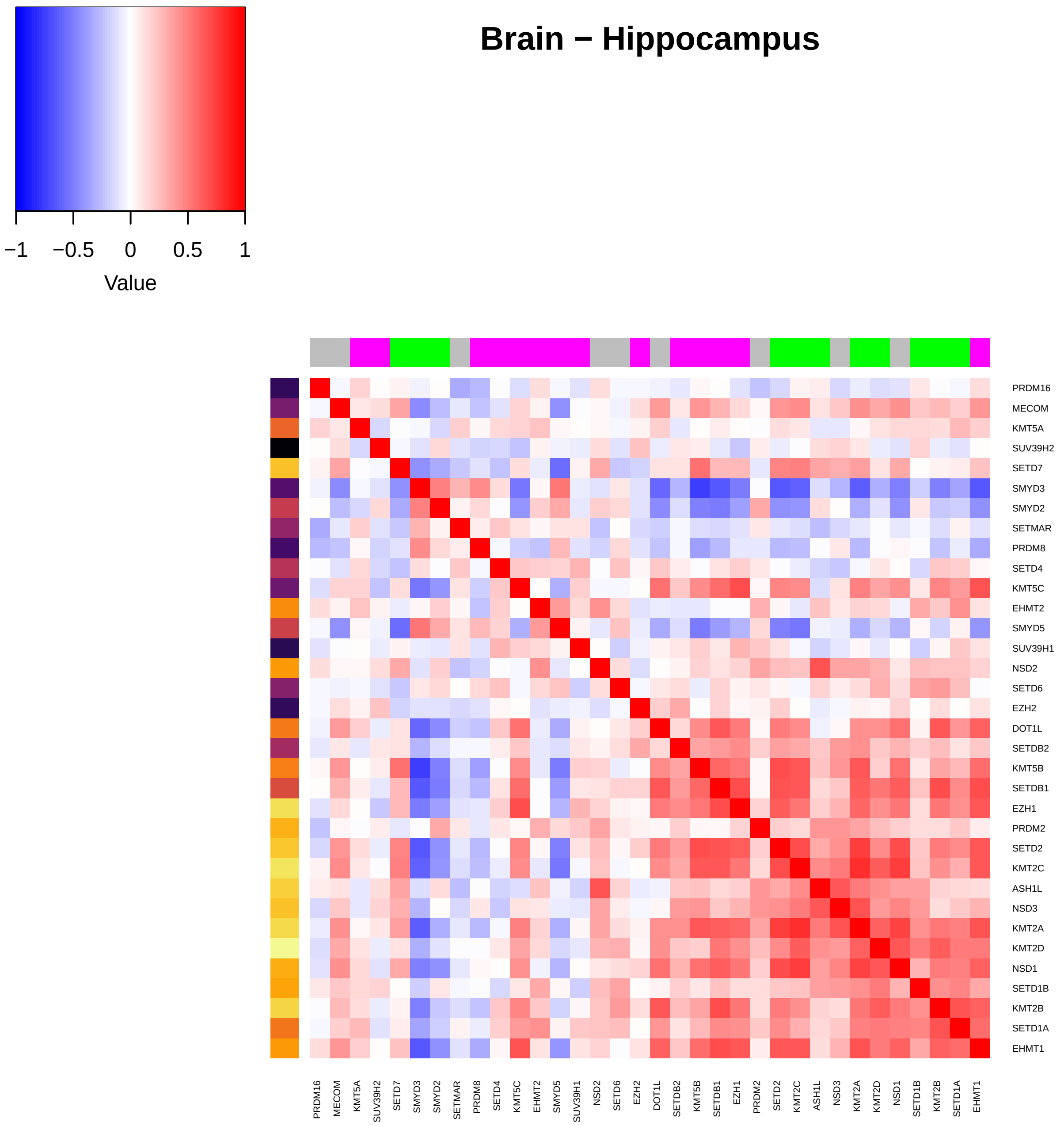

Brain − Hippocampus
−1
−0.5
0
0.5
1
Value
PRDM16
MECOM
KMT5A
SUV39H2
SETD7
SMYD3
SMYD2
SETMAR
PRDM8
SETD4
KMT5C
EHMT2
SMYD5
SUV39H1
NSD2
SETD6
EZH2
DOT1L
SETDB2
KMT5B
SETDB1
EZH1
PRDM2
SETD2
KMT2C
ASH1L
NSD3
KMT2A
KMT2D
NSD1
SETD1B
KMT2B
SETD1A
EHMT1

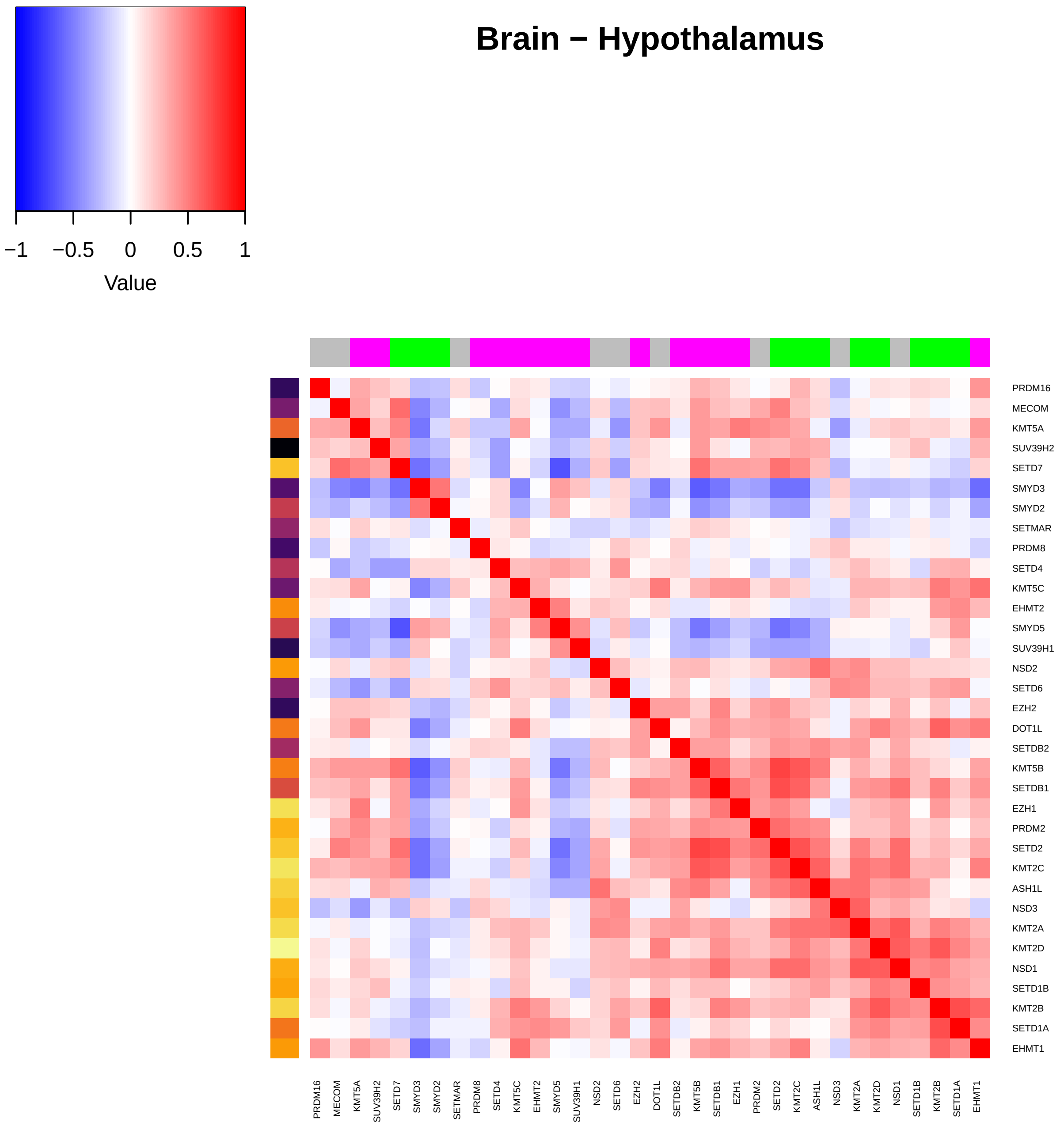
Brain − Hypothalamus
Value
−1
−0.5
0
0.5
1
PRDM16
MECOM
KMT5A
SUV39H2
SETD7
SMYD3
SMYD2
SETMAR
PRDM8
SETD4
KMT5C
EHMT2
SMYD5
SUV39H1
NSD2
SETD6
EZH2
DOT1L
SETDB2
KMT5B
SETDB1
EZH1
PRDM2
SETD2
KMT2C
ASH1L
NSD3
KMT2A
KMT2D
NSD1
SETD1B
KMT2B
SETD1A
EHMT1

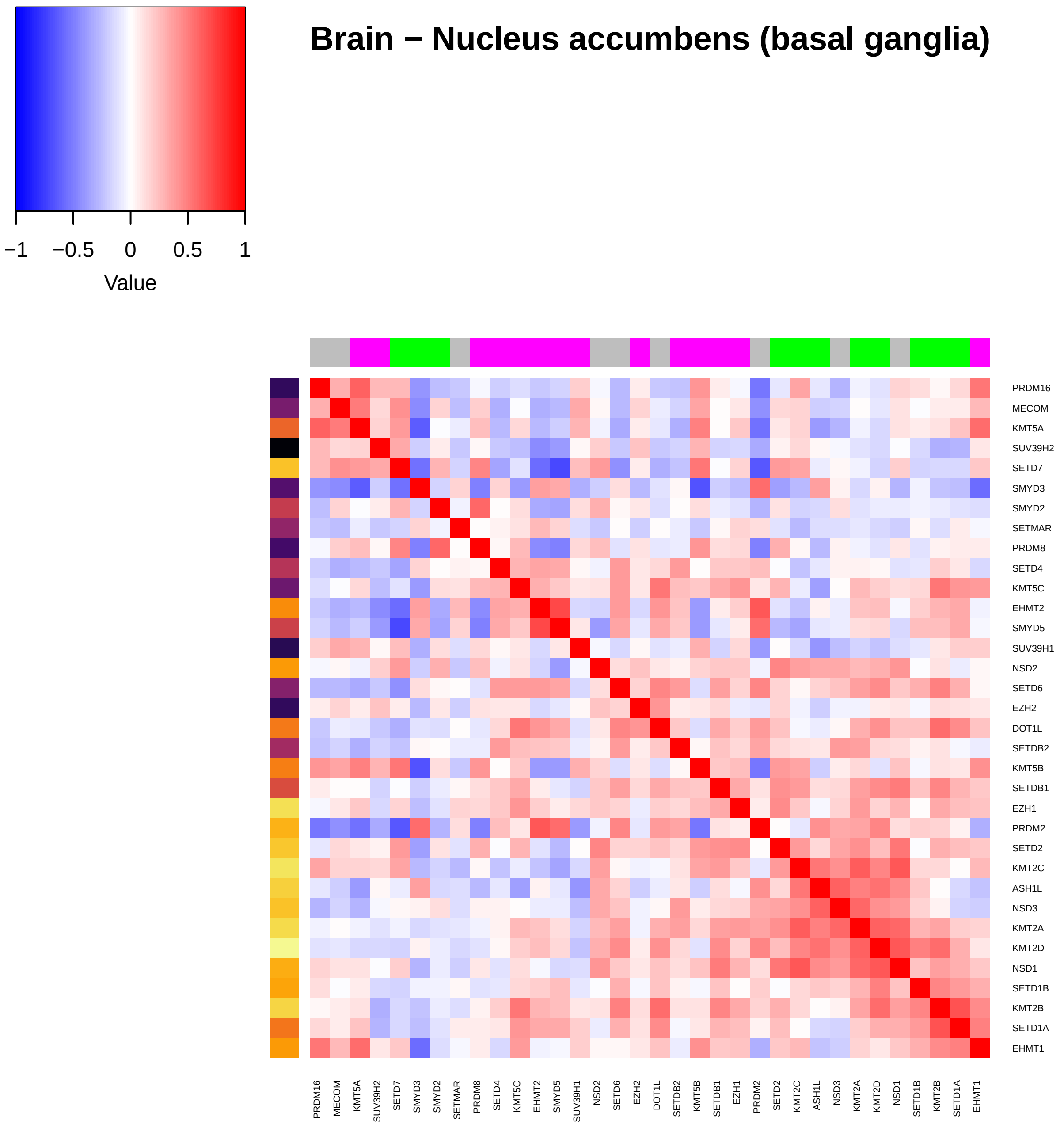
Brain − Nucleus accumbens (basal ganglia)
−1
−0.5
0
0.5
1
Value
PRDM16
MECOM
KMT5A
SUV39H2
SETD7
SMYD3
SMYD2
SETMAR
PRDM8
SETD4
KMT5C
EHMT2
SMYD5
SUV39H1
NSD2
SETD6
EZH2
DOT1L
SETDB2
KMT5B
SETDB1
EZH1
PRDM2
SETD2
KMT2C
ASH1L
NSD3
KMT2A
KMT2D
NSD1
SETD1B
KMT2B
SETD1A
EHMT1

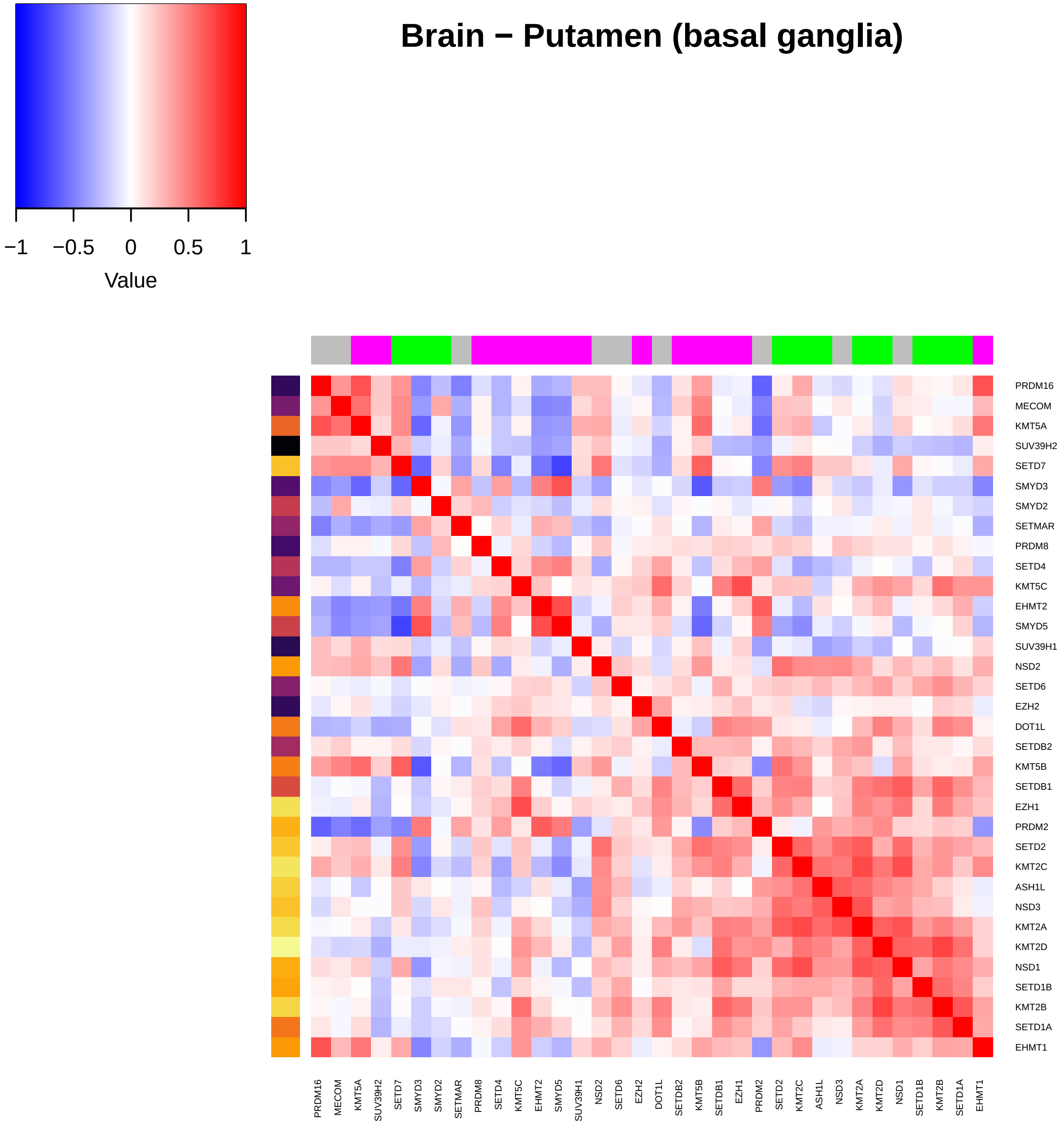
Brain − Putamen (basal ganglia)
−1
−0.5
0
0.5
1
Value
PRDM16
MECOM
KMT5A
SUV39H2
SETD7
SMYD3
SMYD2
SETMAR
PRDM8
SETD4
KMT5C
EHMT2
SMYD5
SUV39H1
NSD2
SETD6
EZH2
DOT1L
SETDB2
KMT5B
SETDB1
EZH1
PRDM2
SETD2
KMT2C
ASH1L
NSD3
KMT2A
KMT2D
NSD1
SETD1B
KMT2B
SETD1A
EHMT1

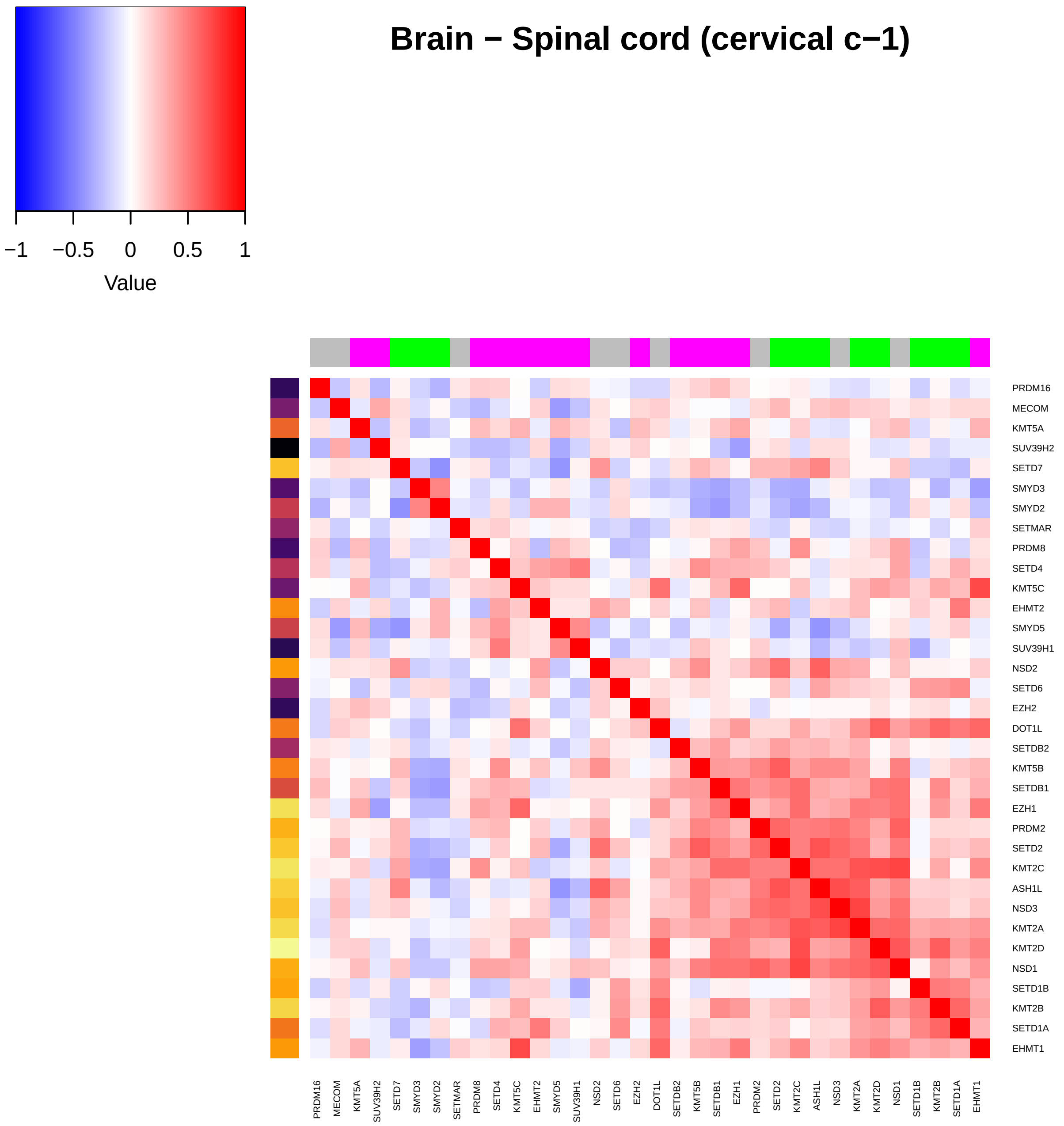
Brain − Spinal cord (cervical c−1)
−1
−0.5
0
0.5
1
Value
PRDM16
MECOM
KMT5A
SUV39H2
SETD7
SMYD3
SMYD2
SETMAR
PRDM8
SETD4
KMT5C
EHMT2
SMYD5
SUV39H1
NSD2
SETD6
EZH2
DOT1L
SETDB2
KMT5B
SETDB1
EZH1
PRDM2
SETD2
KMT2C
ASH1L
NSD3
KMT2A
KMT2D
NSD1
SETD1B
KMT2B
SETD1A
EHMT1

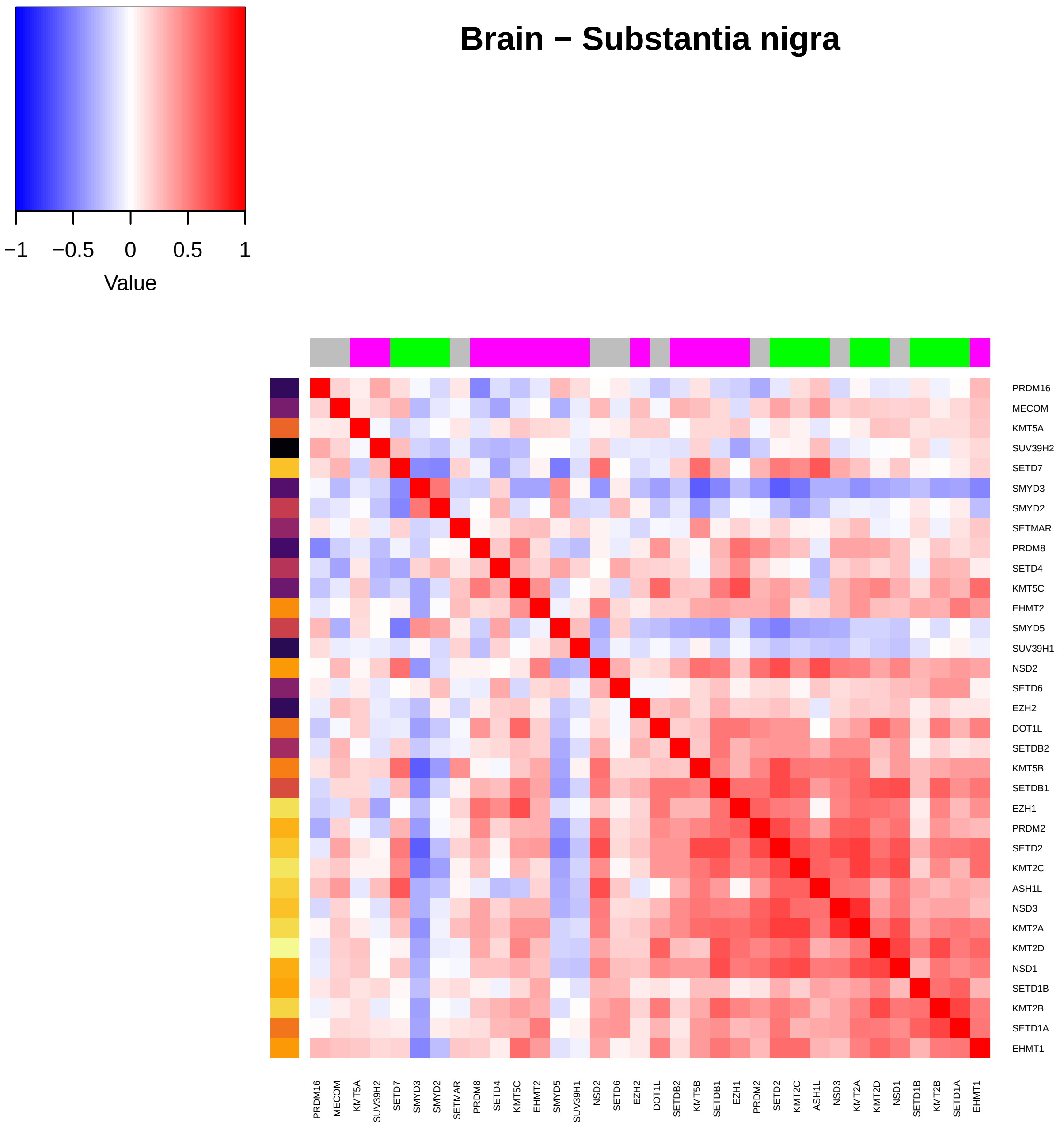
Brain − Substantia nigra
−1
−0.5
0
0.5
1
Value
PRDM16
MECOM
KMT5A
SUV39H2
SETD7
SMYD3
SMYD2
SETMAR
PRDM8
SETD4
KMT5C
EHMT2
SMYD5
SUV39H1
NSD2
SETD6
EZH2
DOT1L
SETDB2
KMT5B
SETDB1
EZH1
PRDM2
SETD2
KMT2C
ASH1L
NSD3
KMT2A
KMT2D
NSD1
SETD1B
KMT2B
SETD1A
EHMT1

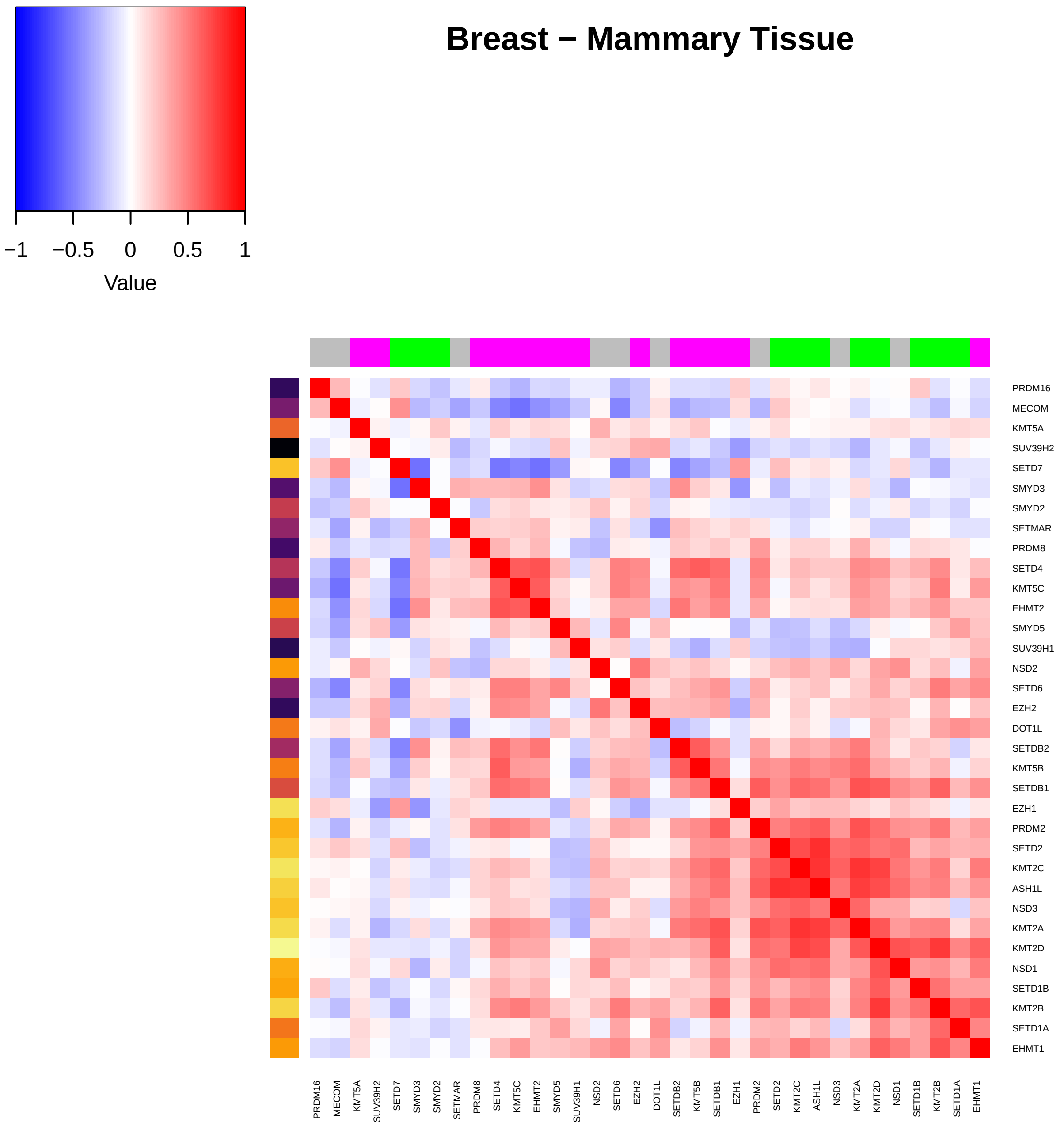
Breast − Mammary Tissue
−1
−0.5
0
0.5
1
Value
PRDM16
MECOM
KMT5A
SUV39H2
SETD7
SMYD3
SMYD2
SETMAR
PRDM8
SETD4
KMT5C
EHMT2
SMYD5
SUV39H1
NSD2
SETD6
EZH2
DOT1L
SETDB2
KMT5B
SETDB1
EZH1
PRDM2
SETD2
KMT2C
ASH1L
NSD3
KMT2A
KMT2D
NSD1
SETD1B
KMT2B
SETD1A
EHMT1

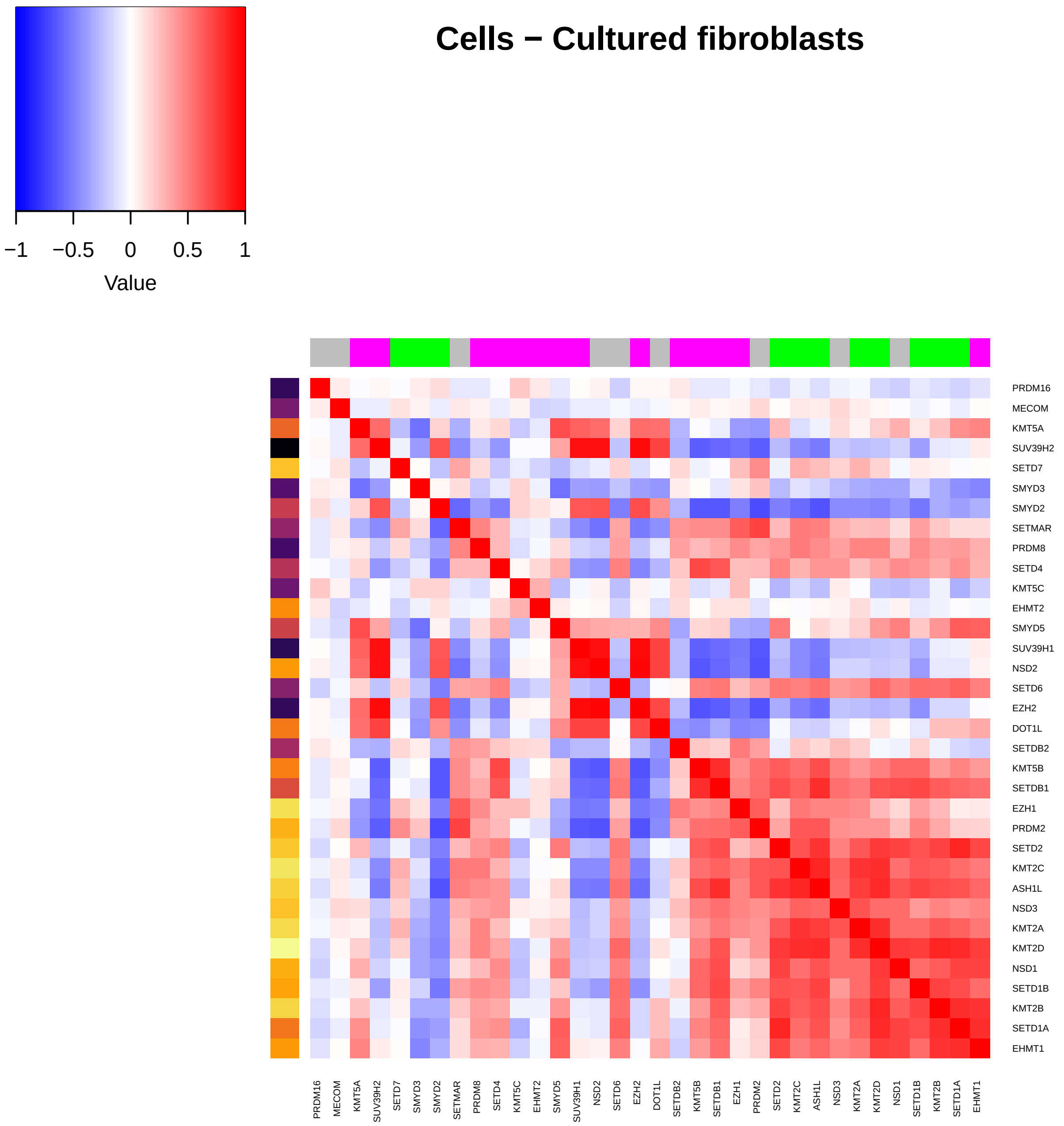

Cells − Cultured fibroblasts
−1
−0.5
0
0.5
1
Value
PRDM16
MECOM
KMT5A
SUV39H2
SETD7
SMYD3
SMYD2
SETMAR
PRDM8
SETD4
KMT5C
EHMT2
SMYD5
SUV39H1
NSD2
SETD6
EZH2
DOT1L
SETDB2
KMT5B
SETDB1
EZH1
PRDM2
SETD2
KMT2C
ASH1L
NSD3
KMT2A
KMT2D
NSD1
SETD1B
KMT2B
SETD1A
EHMT1

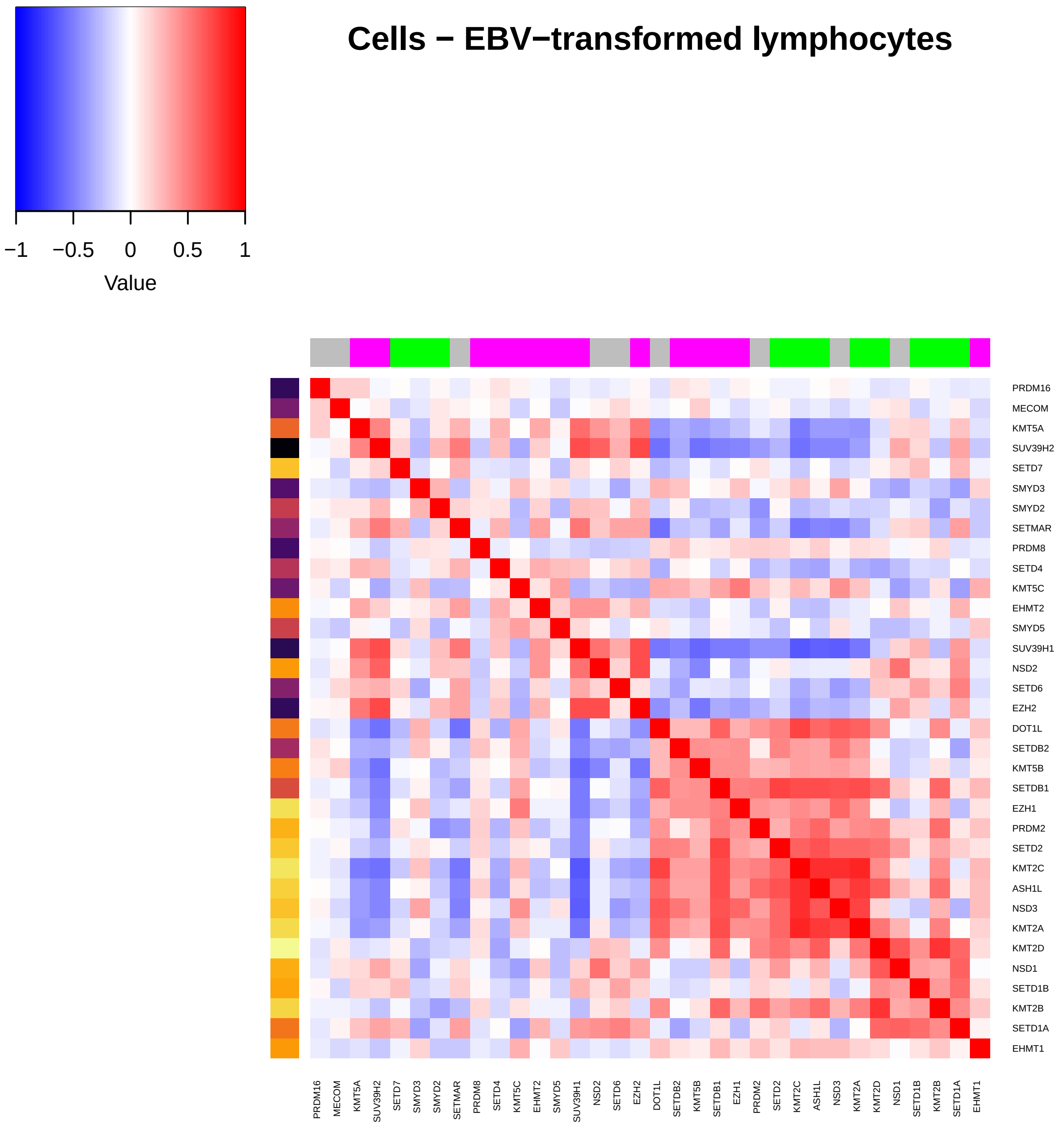

Cells − EBV−transformed lymphocytes
−1
−0.5
0
0.5
1
Value
PRDM16
MECOM
KMT5A
SUV39H2
SETD7
SMYD3
SMYD2
SETMAR
PRDM8
SETD4
KMT5C
EHMT2
SMYD5
SUV39H1
NSD2
SETD6
EZH2
DOT1L
SETDB2
KMT5B
SETDB1
EZH1
PRDM2
SETD2
KMT2C
ASH1L
NSD3
KMT2A
KMT2D
NSD1
SETD1B
KMT2B
SETD1A
EHMT1

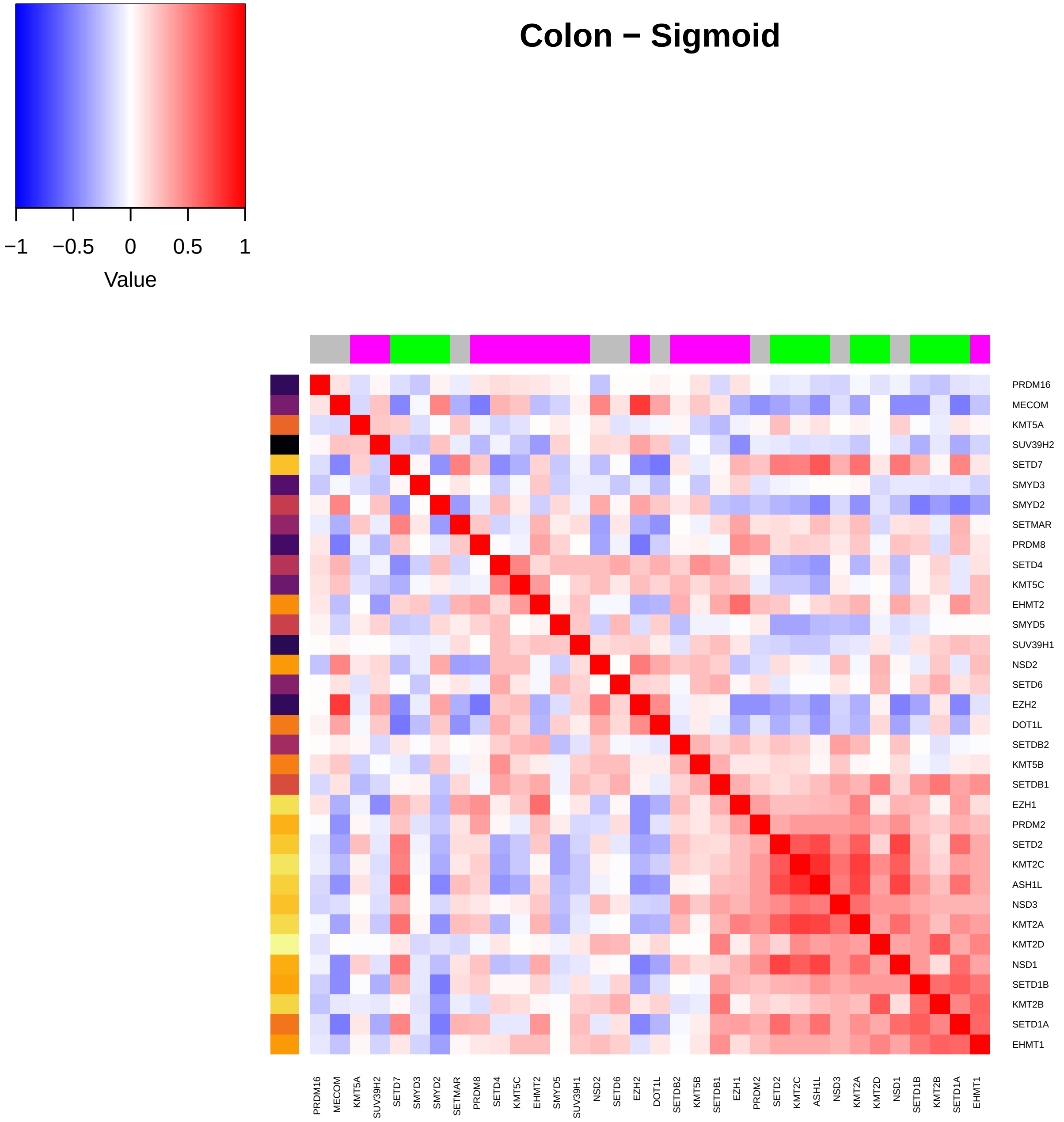
Colon − Sigmoid
−1
−0.5
0
0.5
1
Value
PRDM16
MECOM
KMT5A
SUV39H2
SETD7
SMYD3
SMYD2
SETMAR
PRDM8
SETD4
KMT5C
EHMT2
SMYD5
SUV39H1
NSD2
SETD6
EZH2
DOT1L
SETDB2
KMT5B
SETDB1
EZH1
PRDM2
SETD2
KMT2C
ASH1L
NSD3
KMT2A
KMT2D
NSD1
SETD1B
KMT2B
SETD1A
EHMT1

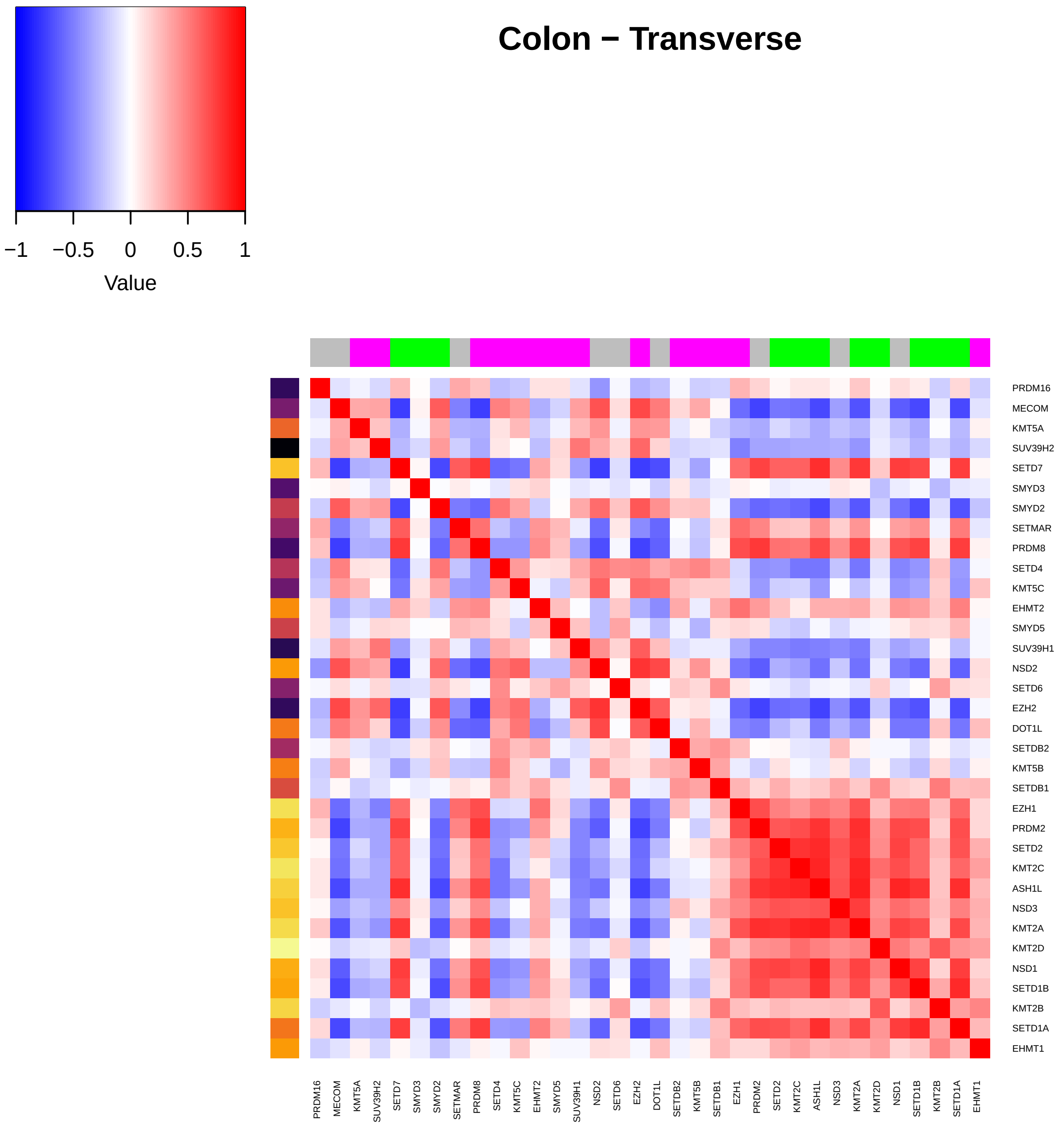
Colon − Transverse
−1
−0.5
0
0.5
1
Value
PRDM16
MECOM
KMT5A
SUV39H2
SETD7
SMYD3
SMYD2
SETMAR
PRDM8
SETD4
KMT5C
EHMT2
SMYD5
SUV39H1
NSD2
SETD6
EZH2
DOT1L
SETDB2
KMT5B
SETDB1
EZH1
PRDM2
SETD2
KMT2C
ASH1L
NSD3
KMT2A
KMT2D
NSD1
SETD1B
KMT2B
SETD1A
EHMT1

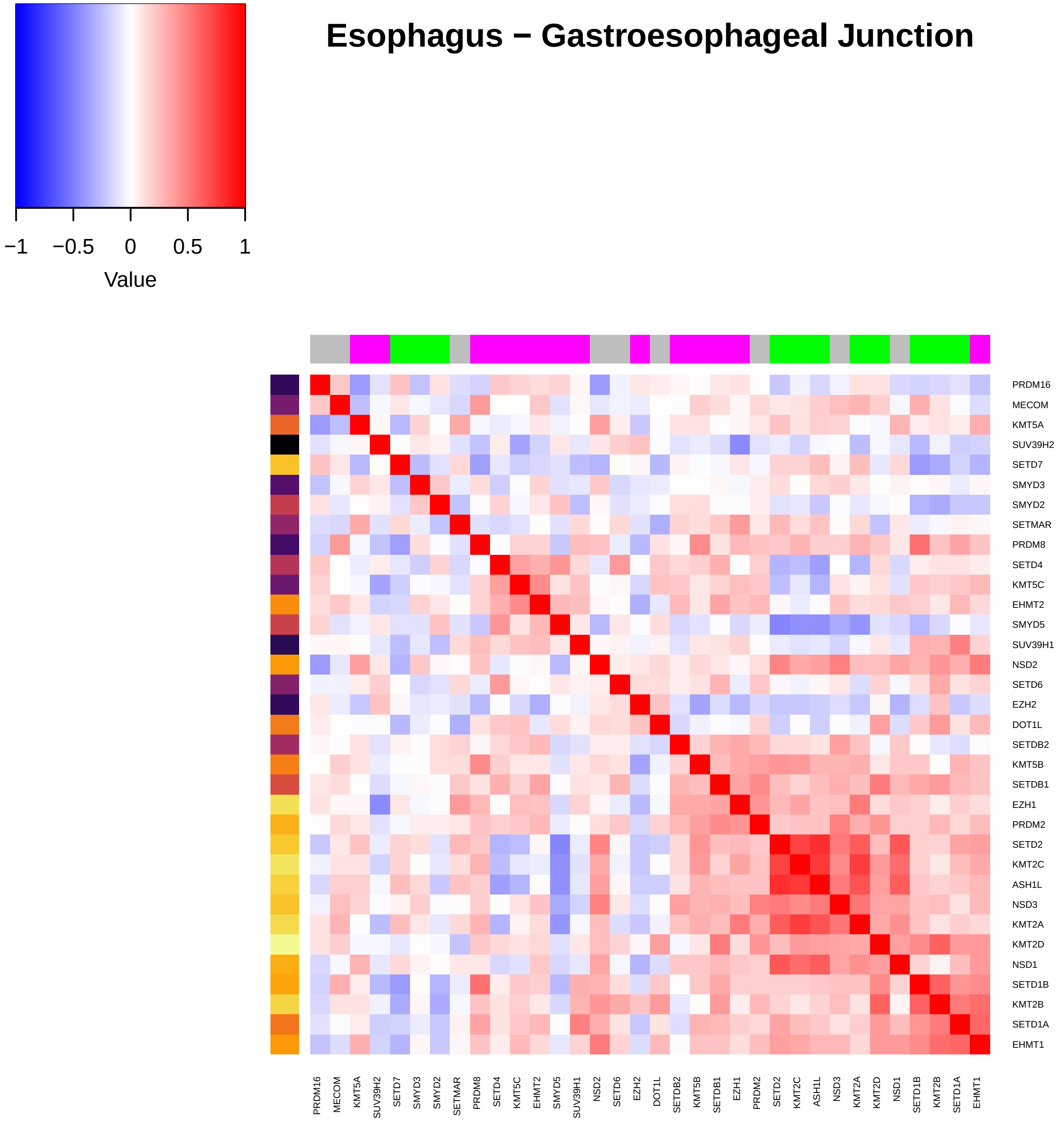
Esophagus − Gastroesophageal Junction
−1
−0.5
0
0.5
1
Value
PRDM16
MECOM
KMT5A
SUV39H2
SETD7
SMYD3
SMYD2
SETMAR
PRDM8
SETD4
KMT5C
EHMT2
SMYD5
SUV39H1
NSD2
SETD6
EZH2
DOT1L
SETDB2
KMT5B
SETDB1
EZH1
PRDM2
SETD2
KMT2C
ASH1L
NSD3
KMT2A
KMT2D
NSD1
SETD1B
KMT2B
SETD1A
EHMT1

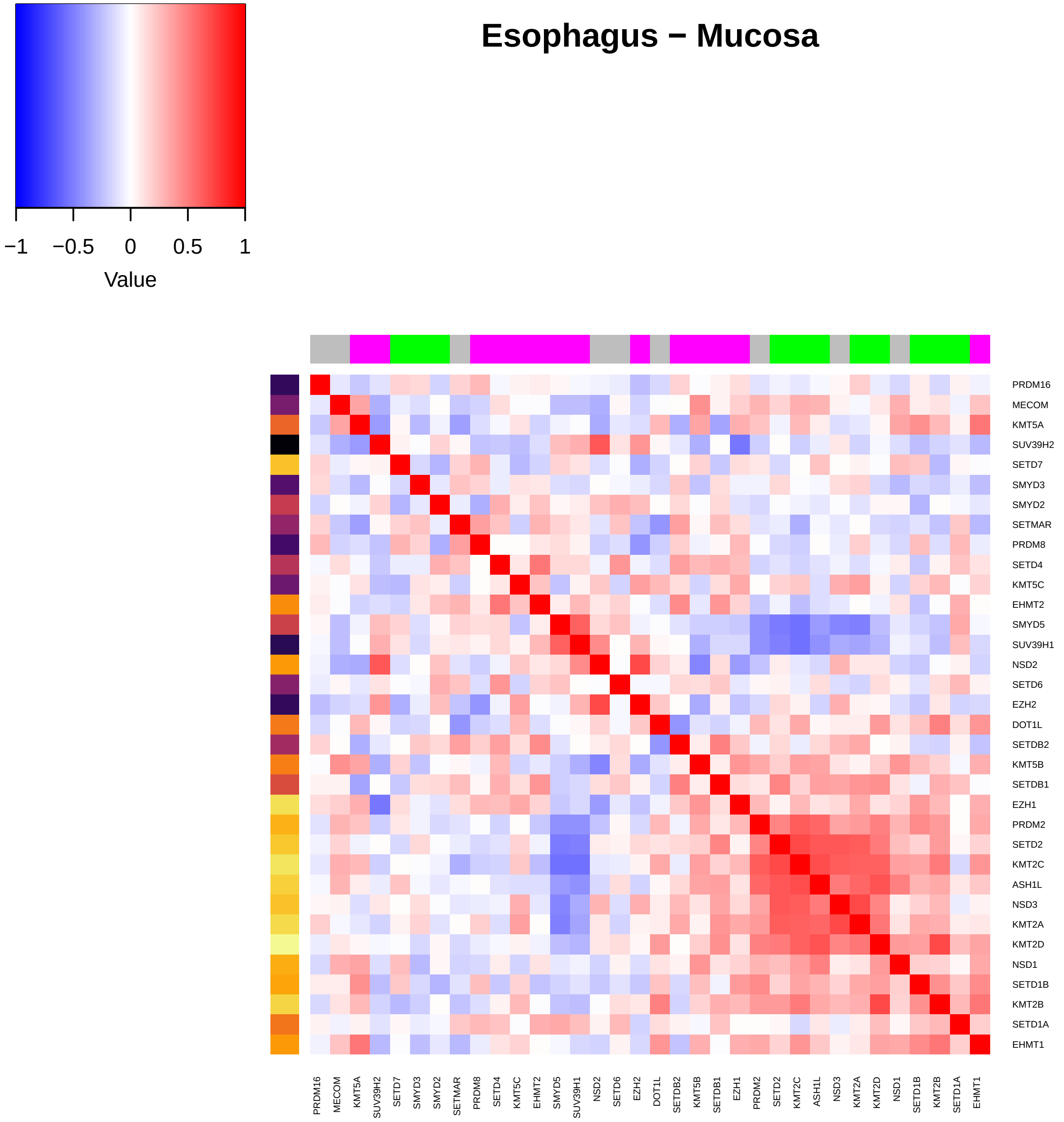

Esophagus − Mucosa
Value
−1
−0.5
0
0.5
1
PRDM16
MECOM
KMT5A
SUV39H2
SETD7
SMYD3
SMYD2
SETMAR
PRDM8
SETD4
KMT5C
EHMT2
SMYD5
SUV39H1
NSD2
SETD6
EZH2
DOT1L
SETDB2
KMT5B
SETDB1
EZH1
PRDM2
SETD2
KMT2C
ASH1L
NSD3
KMT2A
KMT2D
NSD1
SETD1B
KMT2B
SETD1A
EHMT1

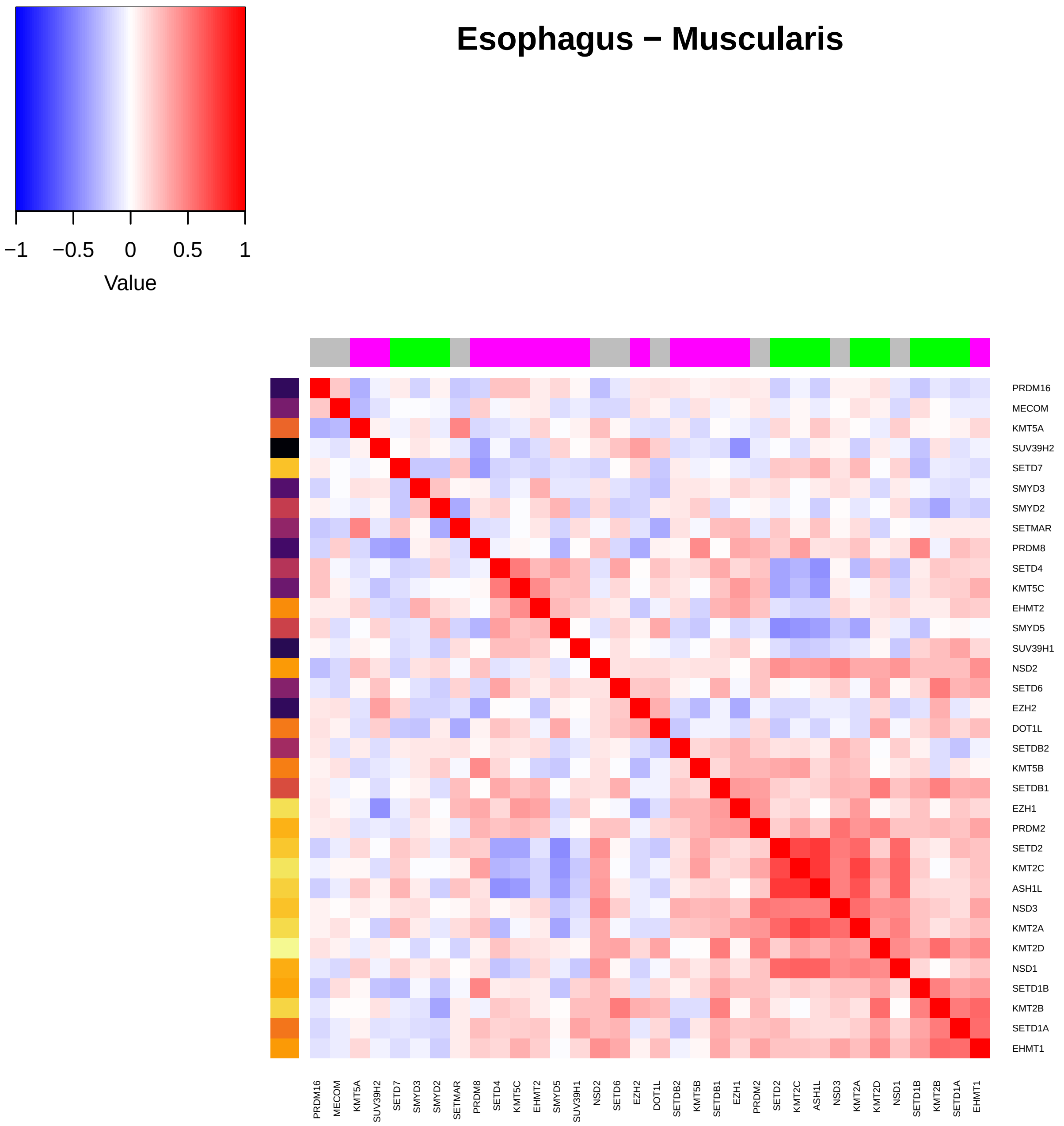

Esophagus − Muscularis
−1
−0.5
0
0.5
1
Value
PRDM16
MECOM
KMT5A
SUV39H2
SETD7
SMYD3
SMYD2
SETMAR
PRDM8
SETD4
KMT5C
EHMT2
SMYD5
SUV39H1
NSD2
SETD6
EZH2
DOT1L
SETDB2
KMT5B
SETDB1
EZH1
PRDM2
SETD2
KMT2C
ASH1L
NSD3
KMT2A
KMT2D
NSD1
SETD1B
KMT2B
SETD1A
EHMT1

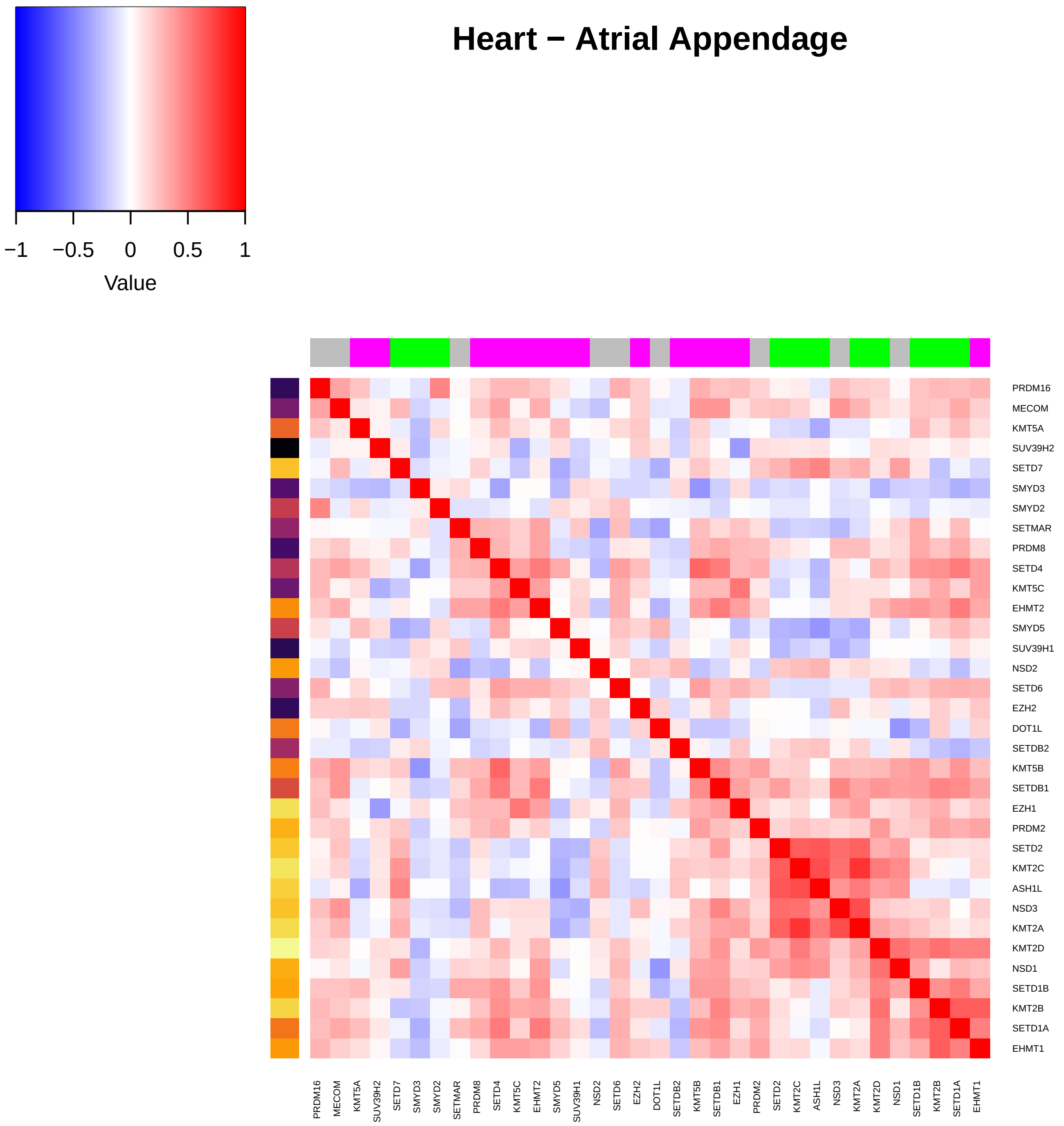

Heart − Atrial Appendage
−1
−0.5
0
0.5
1
Value
PRDM16
MECOM
KMT5A
SUV39H2
SETD7
SMYD3
SMYD2
SETMAR
PRDM8
SETD4
KMT5C
EHMT2
SMYD5
SUV39H1
NSD2
SETD6
EZH2
DOT1L
SETDB2
KMT5B
SETDB1
EZH1
PRDM2
SETD2
KMT2C
ASH1L
NSD3
KMT2A
KMT2D
NSD1
SETD1B
KMT2B
SETD1A
EHMT1

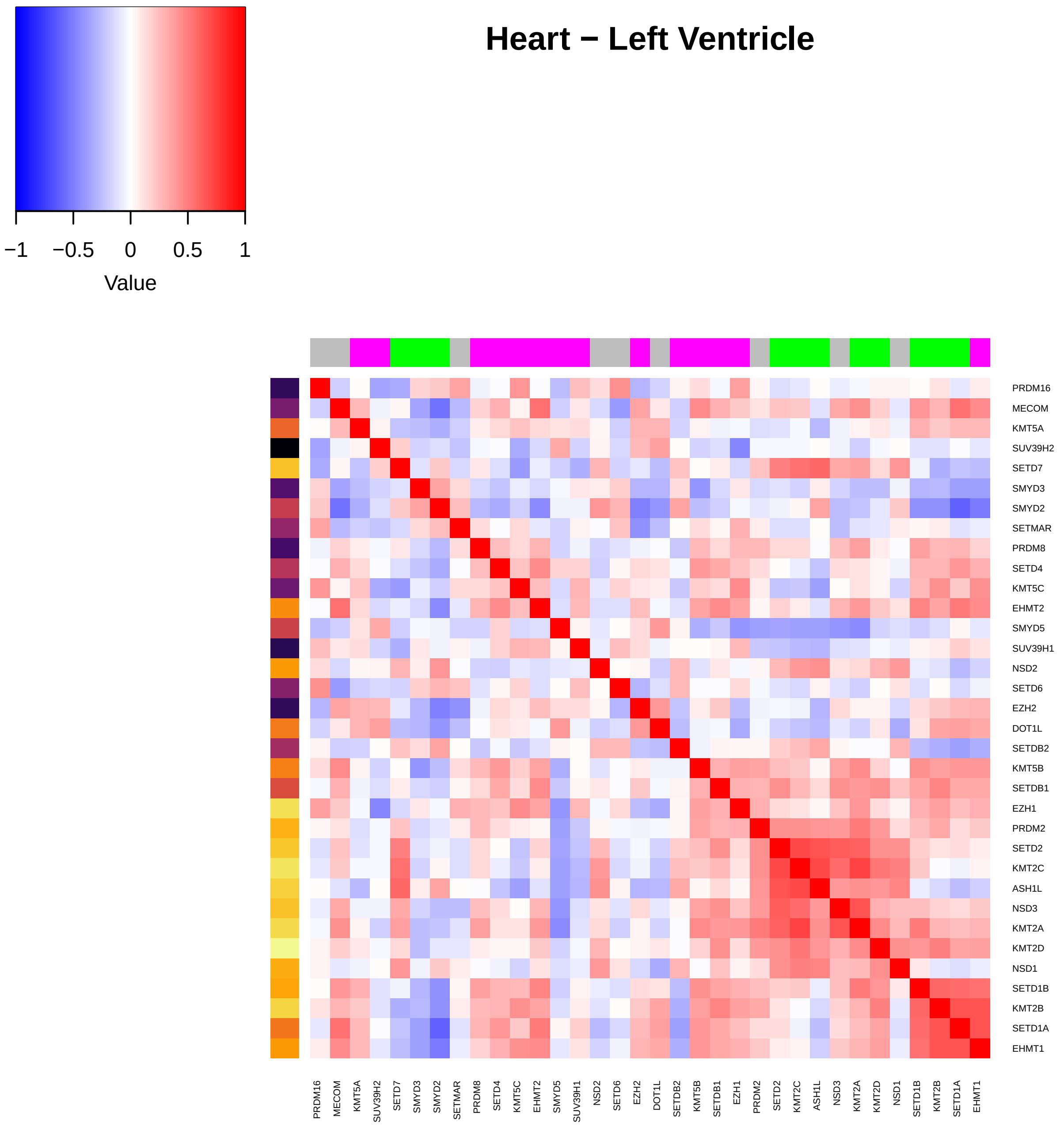

Heart − Left Ventricle
−1
−0.5
0
0.5
1
Value
PRDM16
MECOM
KMT5A
SUV39H2
SETD7
SMYD3
SMYD2
SETMAR
PRDM8
SETD4
KMT5C
EHMT2
SMYD5
SUV39H1
NSD2
SETD6
EZH2
DOT1L
SETDB2
KMT5B
SETDB1
EZH1
PRDM2
SETD2
KMT2C
ASH1L
NSD3
KMT2A
KMT2D
NSD1
SETD1B
KMT2B
SETD1A
EHMT1

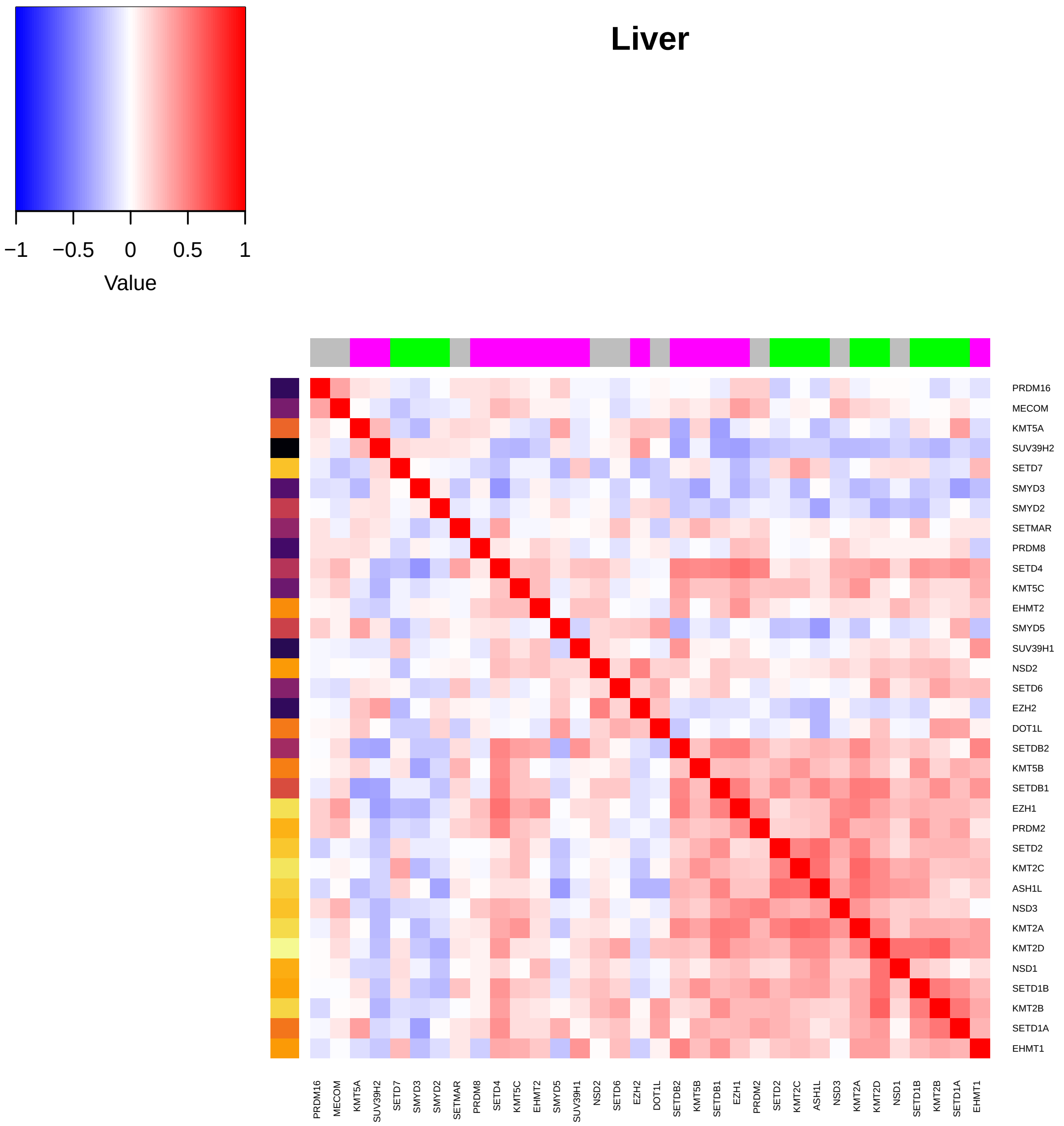

Liver
−1
−0.5
0
0.5
1
Value
PRDM16
MECOM
KMT5A
SUV39H2
SETD7
SMYD3
SMYD2
SETMAR
PRDM8
SETD4
KMT5C
EHMT2
SMYD5
SUV39H1
NSD2
SETD6
EZH2
DOT1L
SETDB2
KMT5B
SETDB1
EZH1
PRDM2
SETD2
KMT2C
ASH1L
NSD3
KMT2A
KMT2D
NSD1
SETD1B
KMT2B
SETD1A
EHMT1

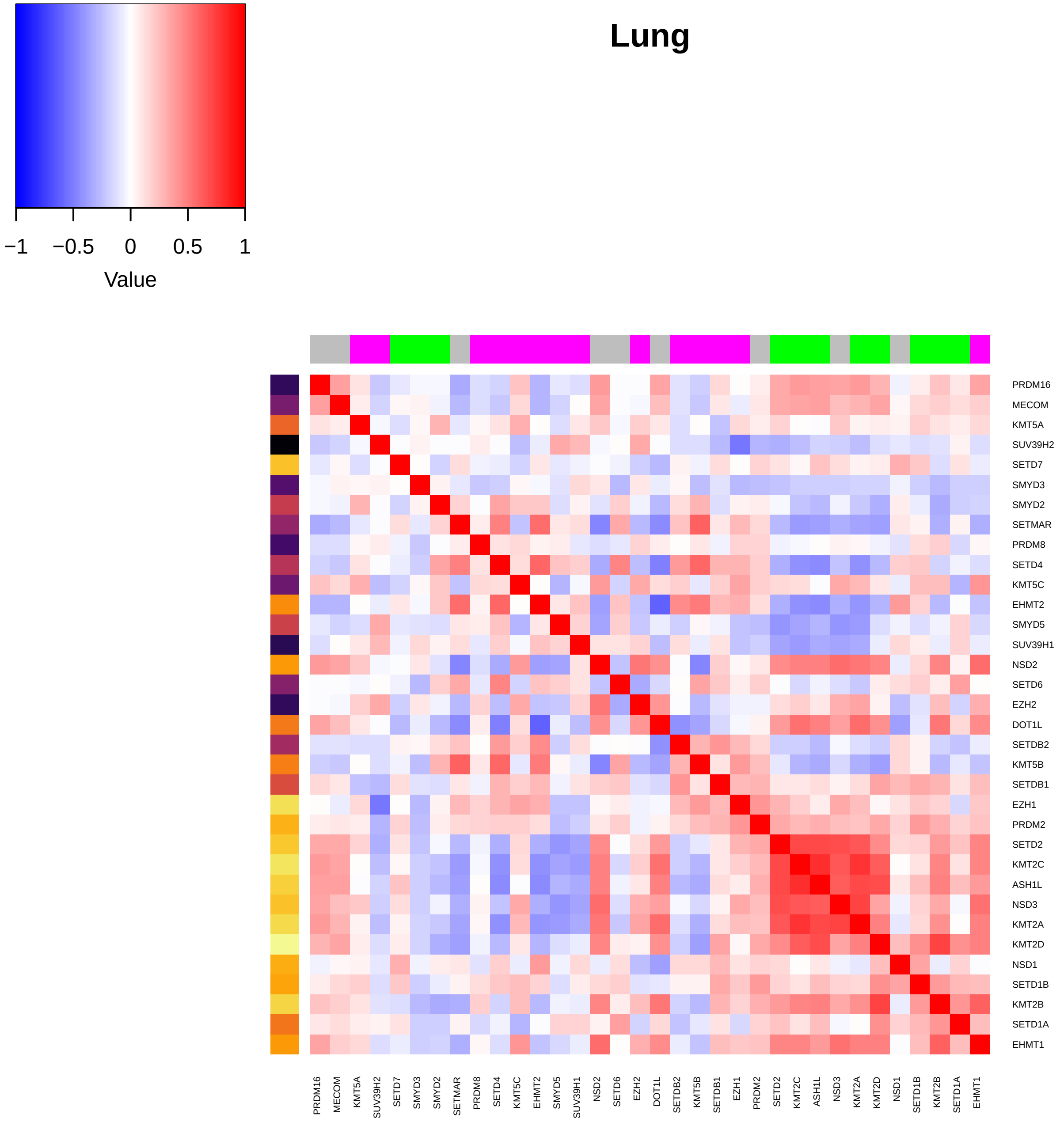

Value
−1
−0.5
0
0.5
1
Lung
PRDM16
MECOM
KMT5A
SUV39H2
SETD7
SMYD3
SMYD2
SETMAR
PRDM8
SETD4
KMT5C
EHMT2
SMYD5
SUV39H1
NSD2
SETD6
EZH2
DOT1L
SETDB2
KMT5B
SETDB1
EZH1
PRDM2
SETD2
KMT2C
ASH1L
NSD3
KMT2A
KMT2D
NSD1
SETD1B
KMT2B
SETD1A
EHMT1

Minor Salivary Gland

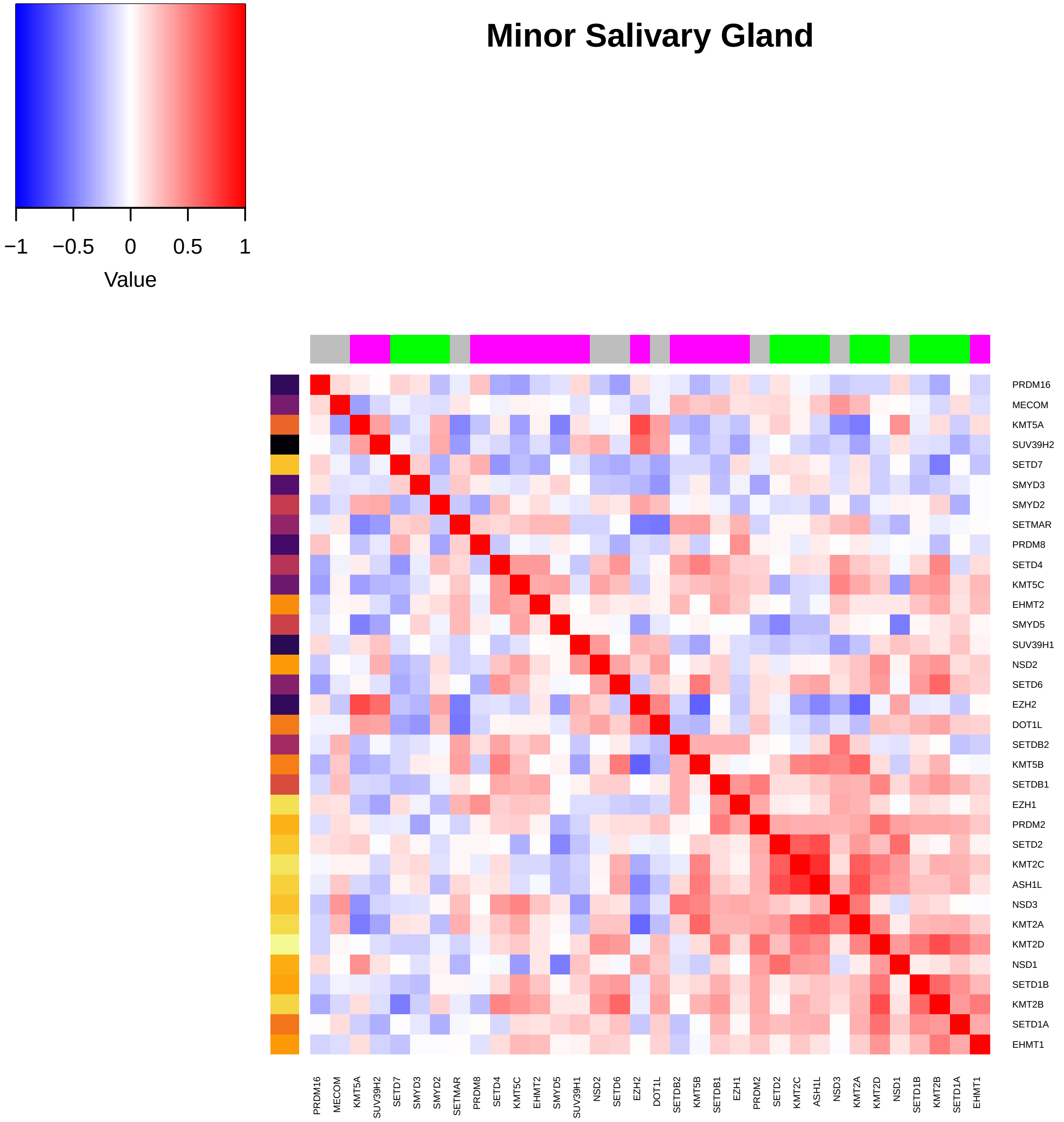

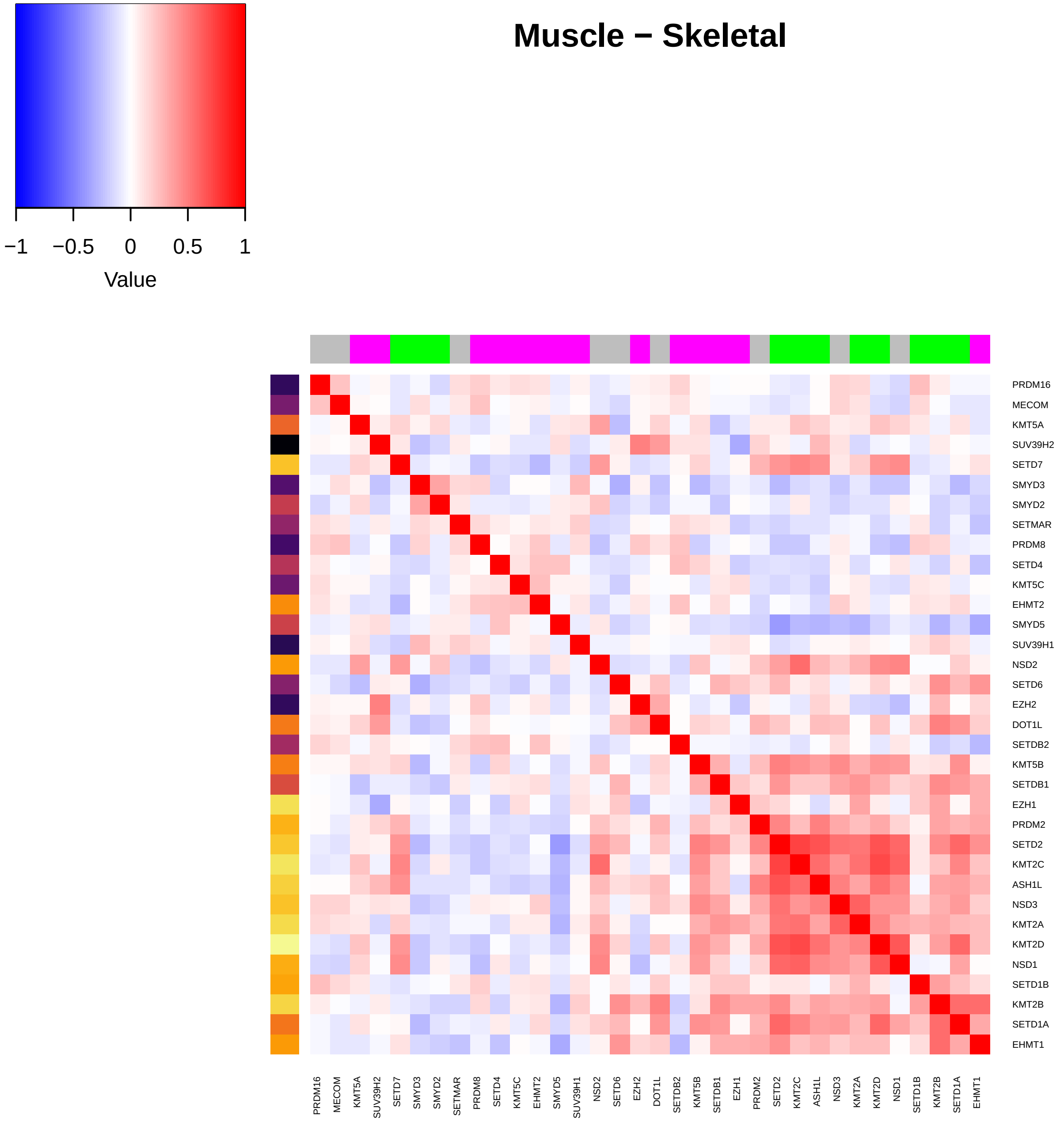
Muscle − Skeletal
Value
−1
−0.5
0
0.5
1
PRDM16
MECOM
KMT5A
SUV39H2
SETD7
SMYD3
SMYD2
SETMAR
PRDM8
SETD4
KMT5C
EHMT2
SMYD5
SUV39H1
NSD2
SETD6
EZH2
DOT1L
SETDB2
KMT5B
SETDB1
EZH1
PRDM2
SETD2
KMT2C
ASH1L
NSD3
KMT2A
KMT2D
NSD1
SETD1B
KMT2B
SETD1A
EHMT1

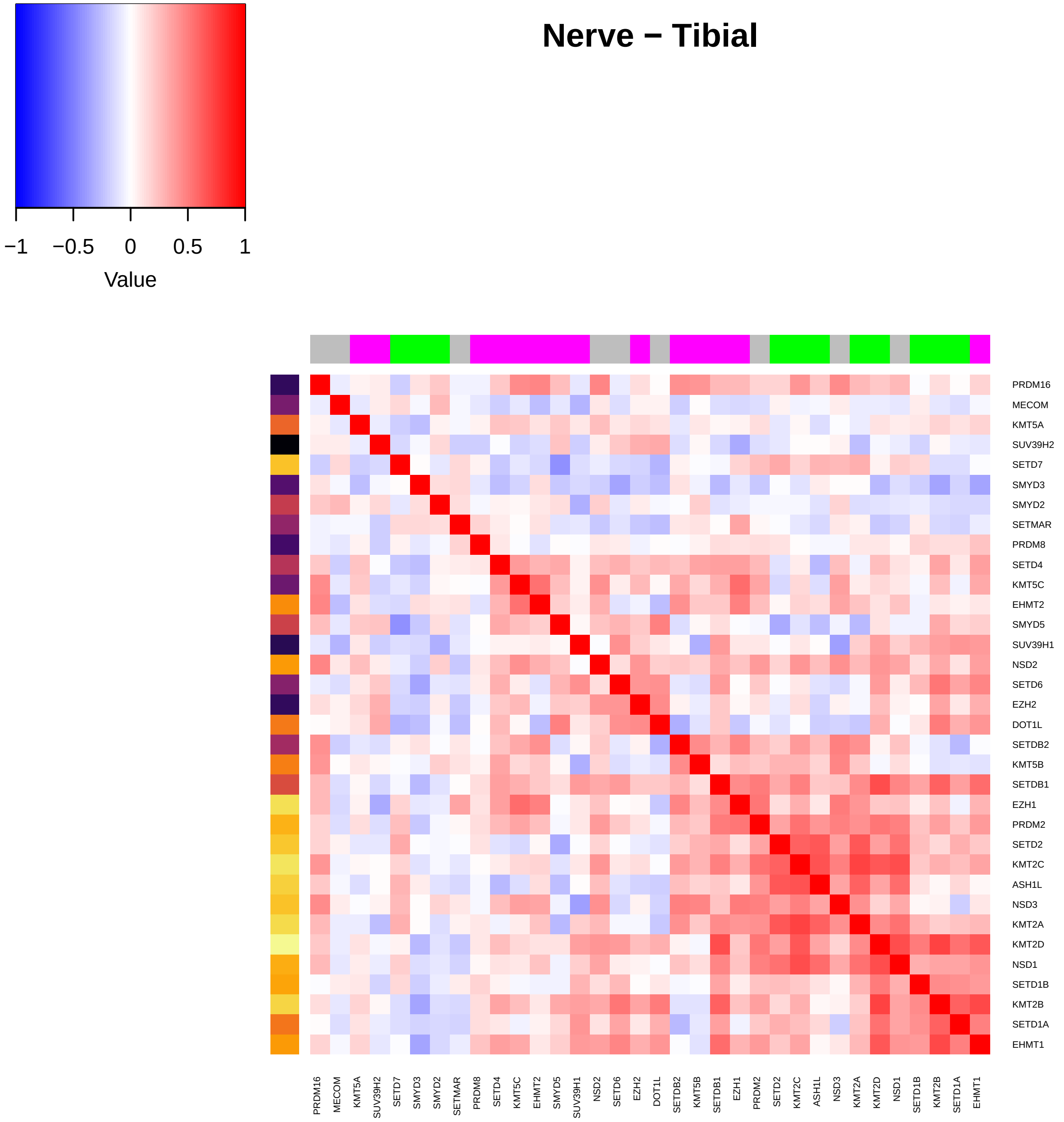

Nerve − Tibial
−1
−0.5
0
0.5
1
Value
PRDM16
MECOM
KMT5A
SUV39H2
SETD7
SMYD3
SMYD2
SETMAR
PRDM8
SETD4
KMT5C
EHMT2
SMYD5
SUV39H1
NSD2
SETD6
EZH2
DOT1L
SETDB2
KMT5B
SETDB1
EZH1
PRDM2
SETD2
KMT2C
ASH1L
NSD3
KMT2A
KMT2D
NSD1
SETD1B
KMT2B
SETD1A
EHMT1

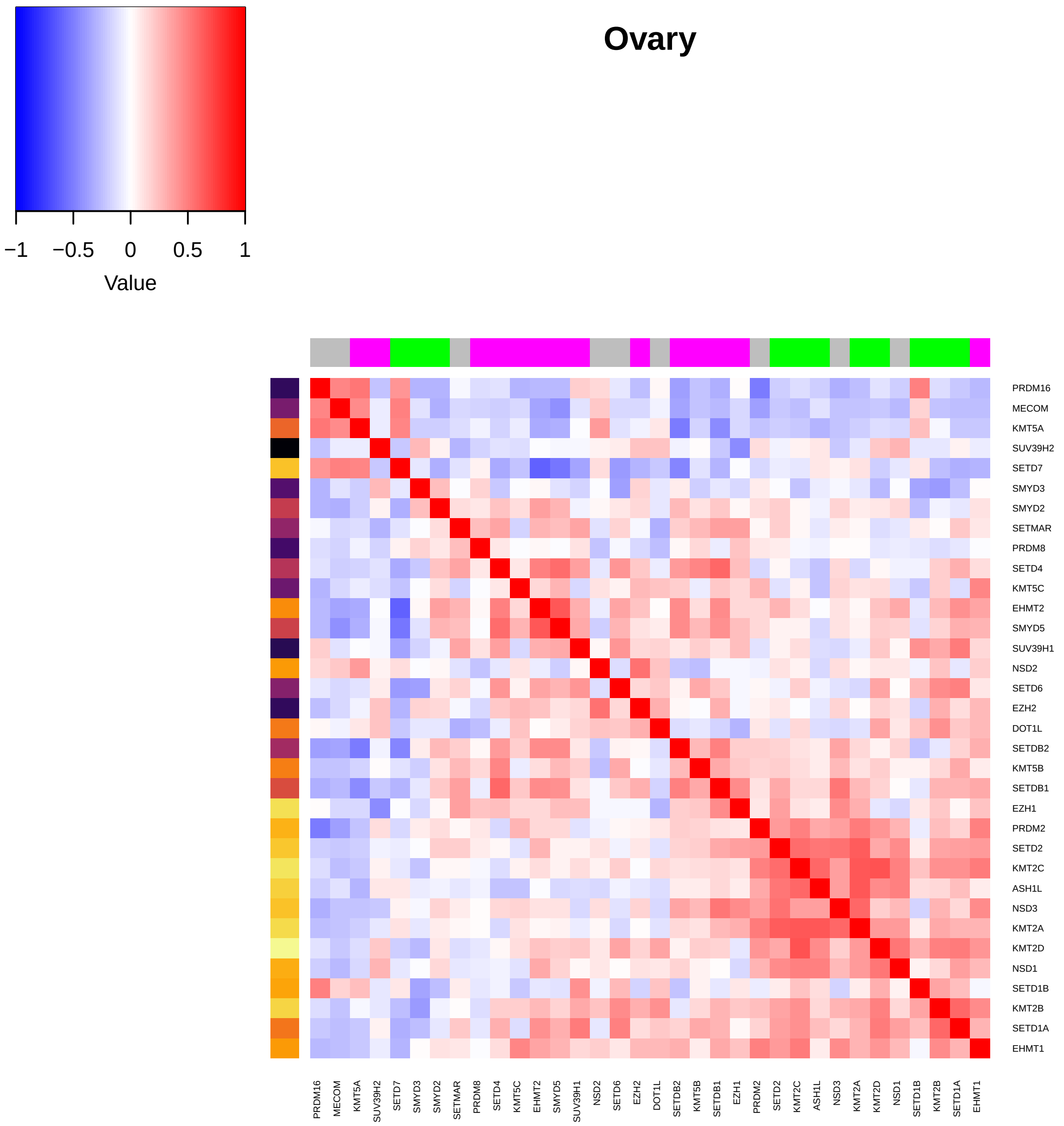

Ovary
−1
−0.5
0
0.5
1
Value
PRDM16
MECOM
KMT5A
SUV39H2
SETD7
SMYD3
SMYD2
SETMAR
PRDM8
SETD4
KMT5C
EHMT2
SMYD5
SUV39H1
NSD2
SETD6
EZH2
DOT1L
SETDB2
KMT5B
SETDB1
EZH1
PRDM2
SETD2
KMT2C
ASH1L
NSD3
KMT2A
KMT2D
NSD1
SETD1B
KMT2B
SETD1A
EHMT1

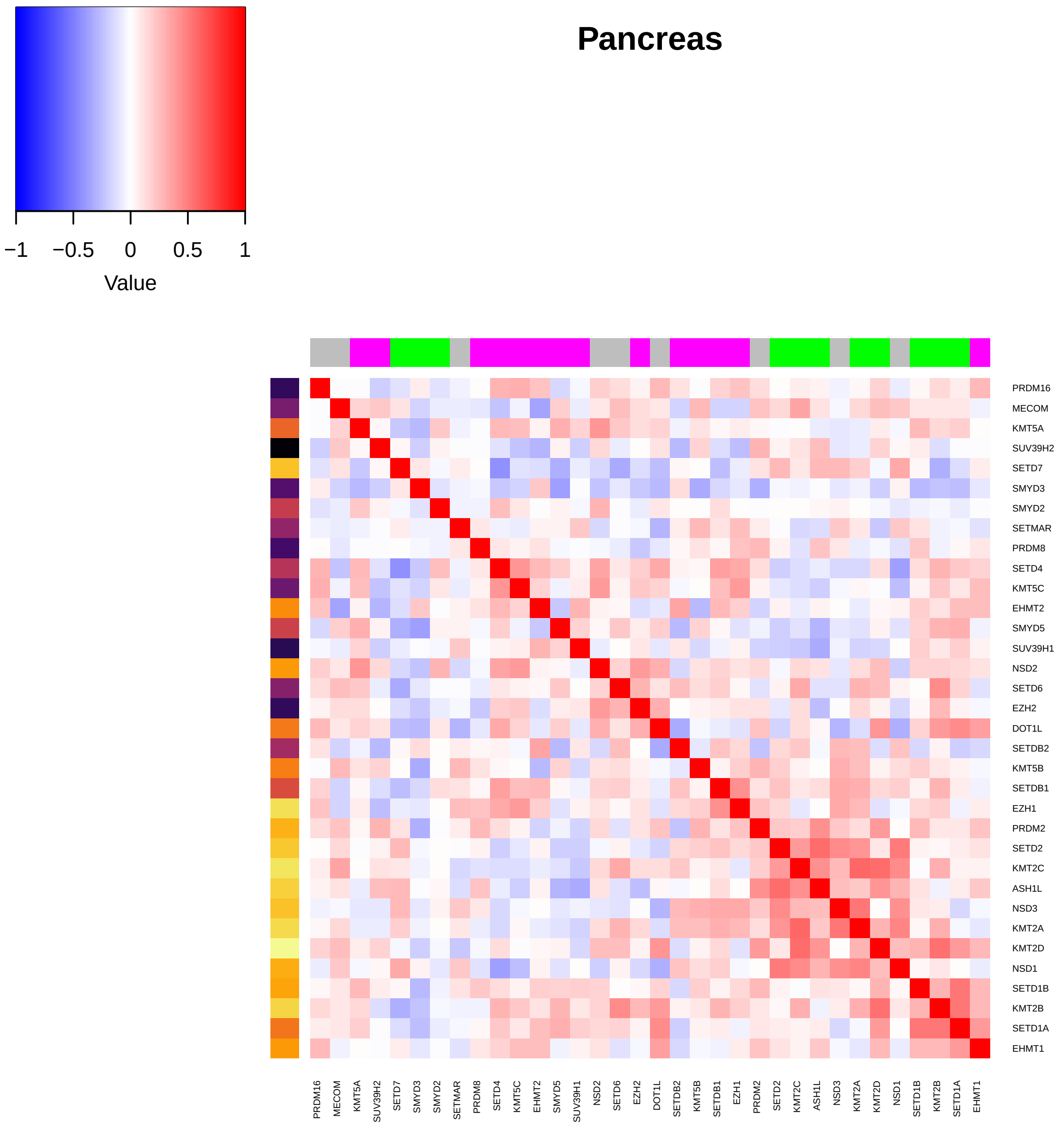
Pancreas
Value
−1
−0.5
0
0.5
1
PRDM16
MECOM
KMT5A
SUV39H2
SETD7
SMYD3
SMYD2
SETMAR
PRDM8
SETD4
KMT5C
EHMT2
SMYD5
SUV39H1
NSD2
SETD6
EZH2
DOT1L
SETDB2
KMT5B
SETDB1
EZH1
PRDM2
SETD2
KMT2C
ASH1L
NSD3
KMT2A
KMT2D
NSD1
SETD1B
KMT2B
SETD1A
EHMT1

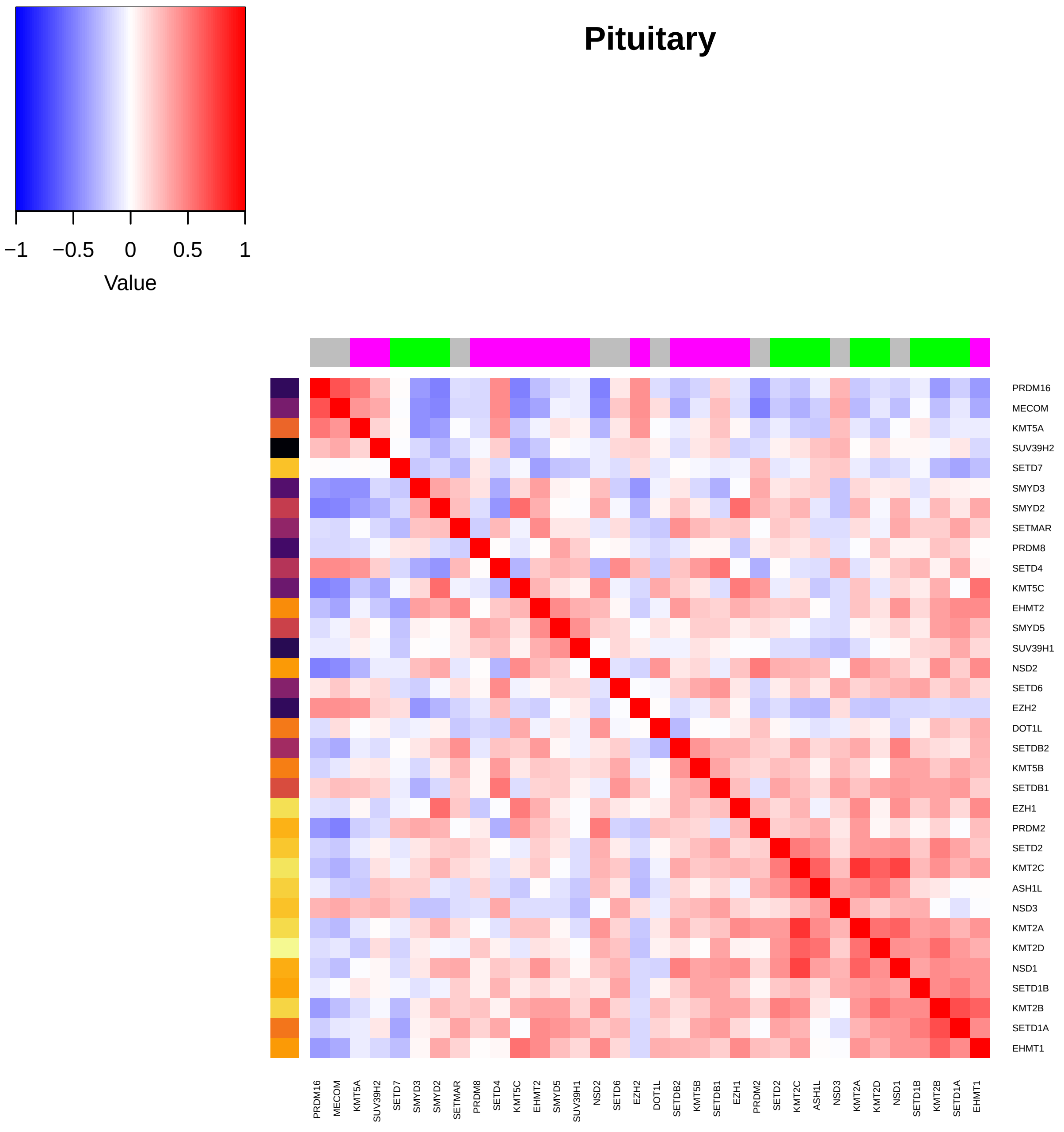

Pituitary
−1
−0.5
0
0.5
1
Value
PRDM16
MECOM
KMT5A
SUV39H2
SETD7
SMYD3
SMYD2
SETMAR
PRDM8
SETD4
KMT5C
EHMT2
SMYD5
SUV39H1
NSD2
SETD6
EZH2
DOT1L
SETDB2
KMT5B
SETDB1
EZH1
PRDM2
SETD2
KMT2C
ASH1L
NSD3
KMT2A
KMT2D
NSD1
SETD1B
KMT2B
SETD1A
EHMT1

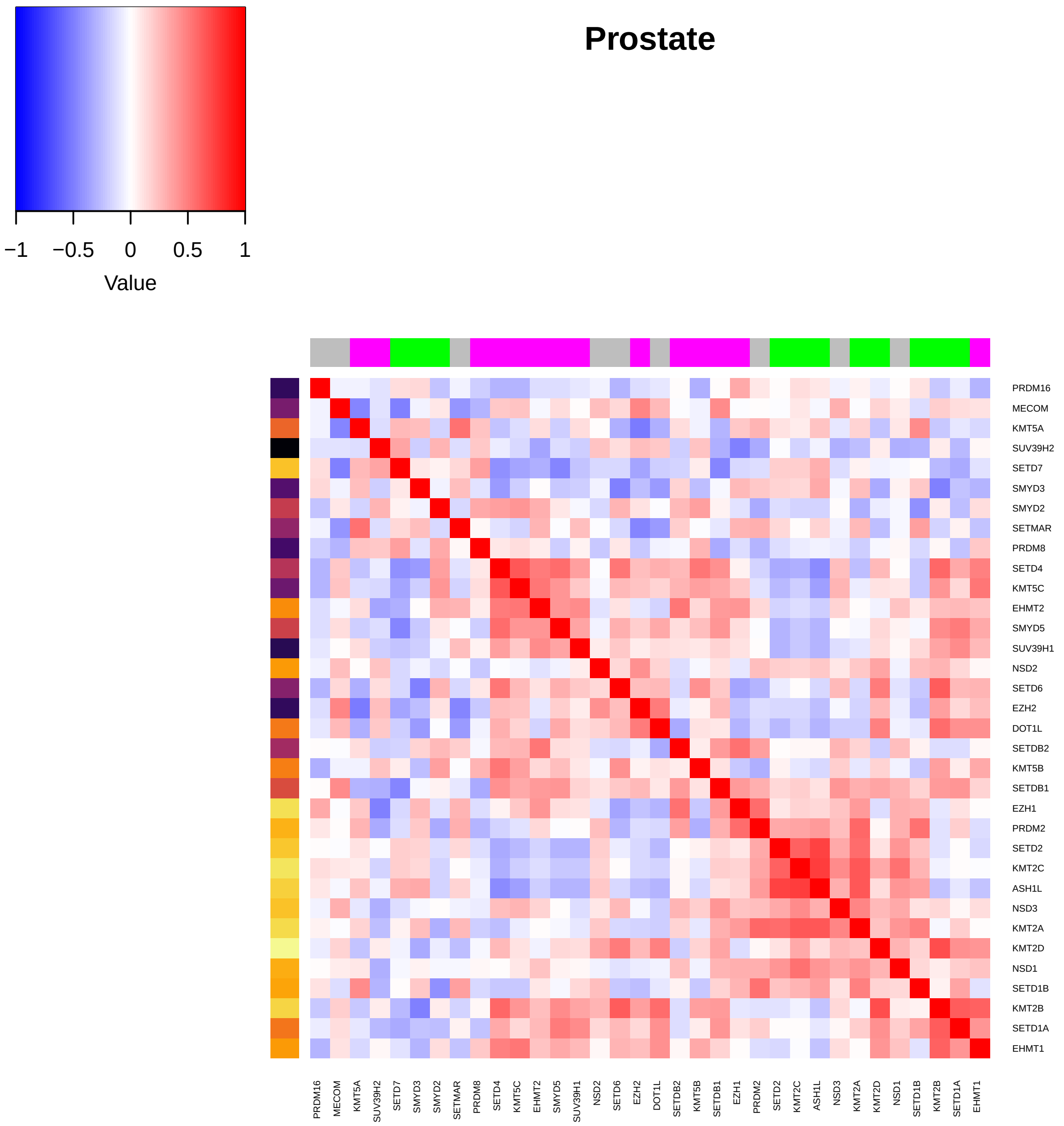

Prostate
−1
−0.5
0
0.5
1
Value
PRDM16
MECOM
KMT5A
SUV39H2
SETD7
SMYD3
SMYD2
SETMAR
PRDM8
SETD4
KMT5C
EHMT2
SMYD5
SUV39H1
NSD2
SETD6
EZH2
DOT1L
SETDB2
KMT5B
SETDB1
EZH1
PRDM2
SETD2
KMT2C
ASH1L
NSD3
KMT2A
KMT2D
NSD1
SETD1B
KMT2B
SETD1A
EHMT1

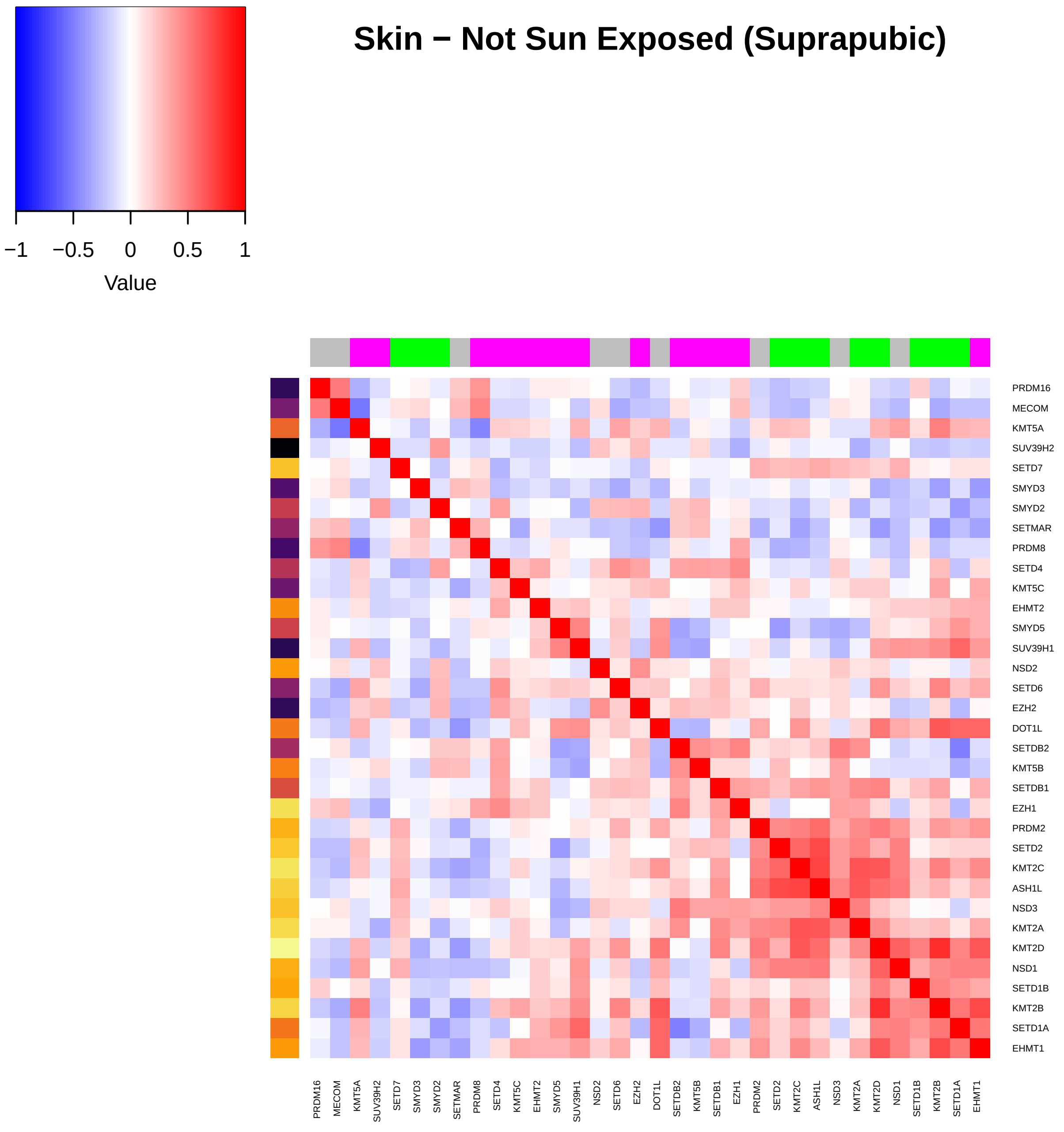
Skin − Not Sun Exposed (Suprapubic)
Value
−1
−0.5
0
0.5
1
PRDM16
MECOM
KMT5A
SUV39H2
SETD7
SMYD3
SMYD2
SETMAR
PRDM8
SETD4
KMT5C
EHMT2
SMYD5
SUV39H1
NSD2
SETD6
EZH2
DOT1L
SETDB2
KMT5B
SETDB1
EZH1
PRDM2
SETD2
KMT2C
ASH1L
NSD3
KMT2A
KMT2D
NSD1
SETD1B
KMT2B
SETD1A
EHMT1

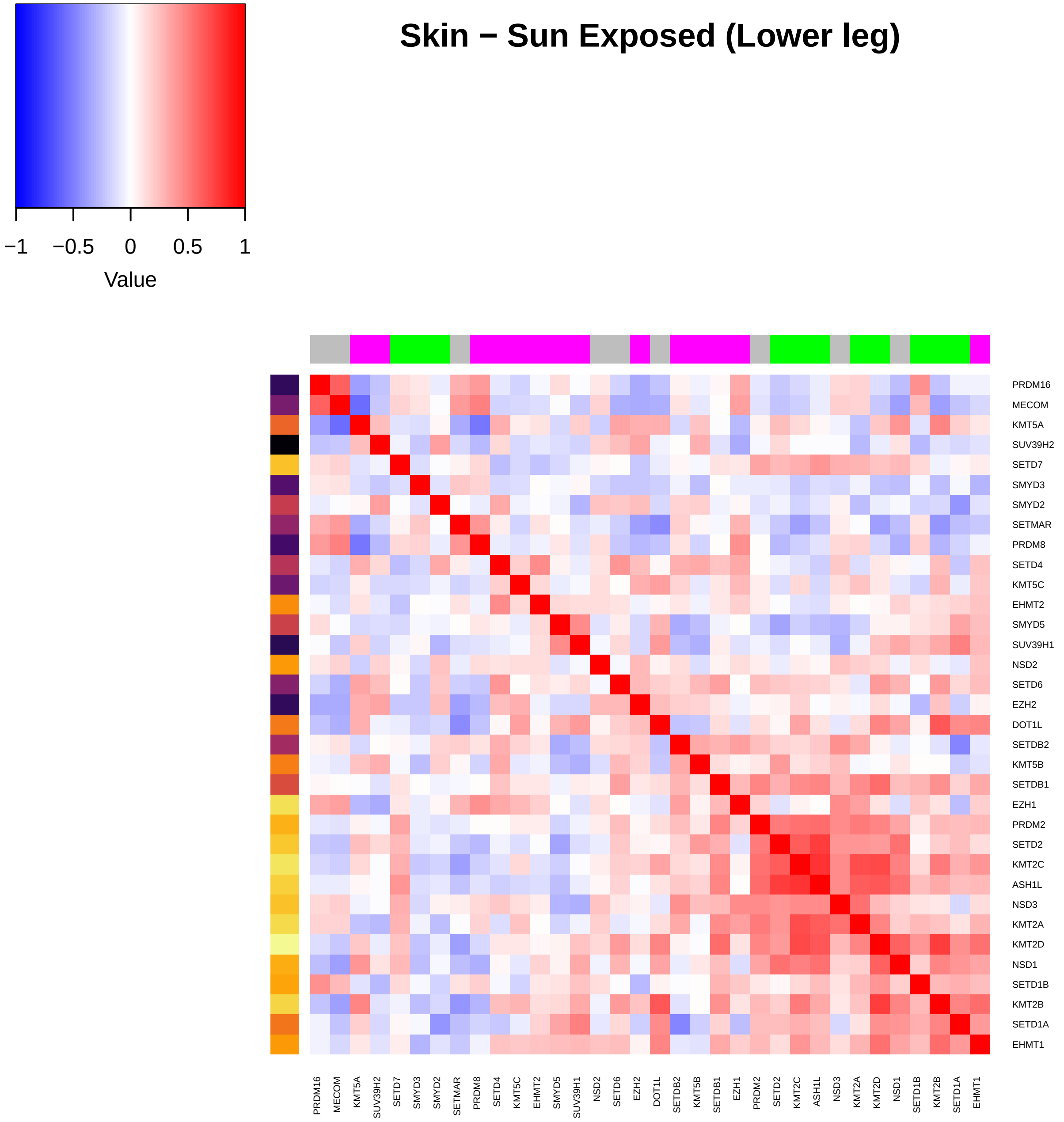
Skin − Sun Exposed (Lower leg)
−1
−0.5
0
0.5
1
Value
PRDM16
MECOM
KMT5A
SUV39H2
SETD7
SMYD3
SMYD2
SETMAR
PRDM8
SETD4
KMT5C
EHMT2
SMYD5
SUV39H1
NSD2
SETD6
EZH2
DOT1L
SETDB2
KMT5B
SETDB1
EZH1
PRDM2
SETD2
KMT2C
ASH1L
NSD3
KMT2A
KMT2D
NSD1
SETD1B
KMT2B
SETD1A
EHMT1

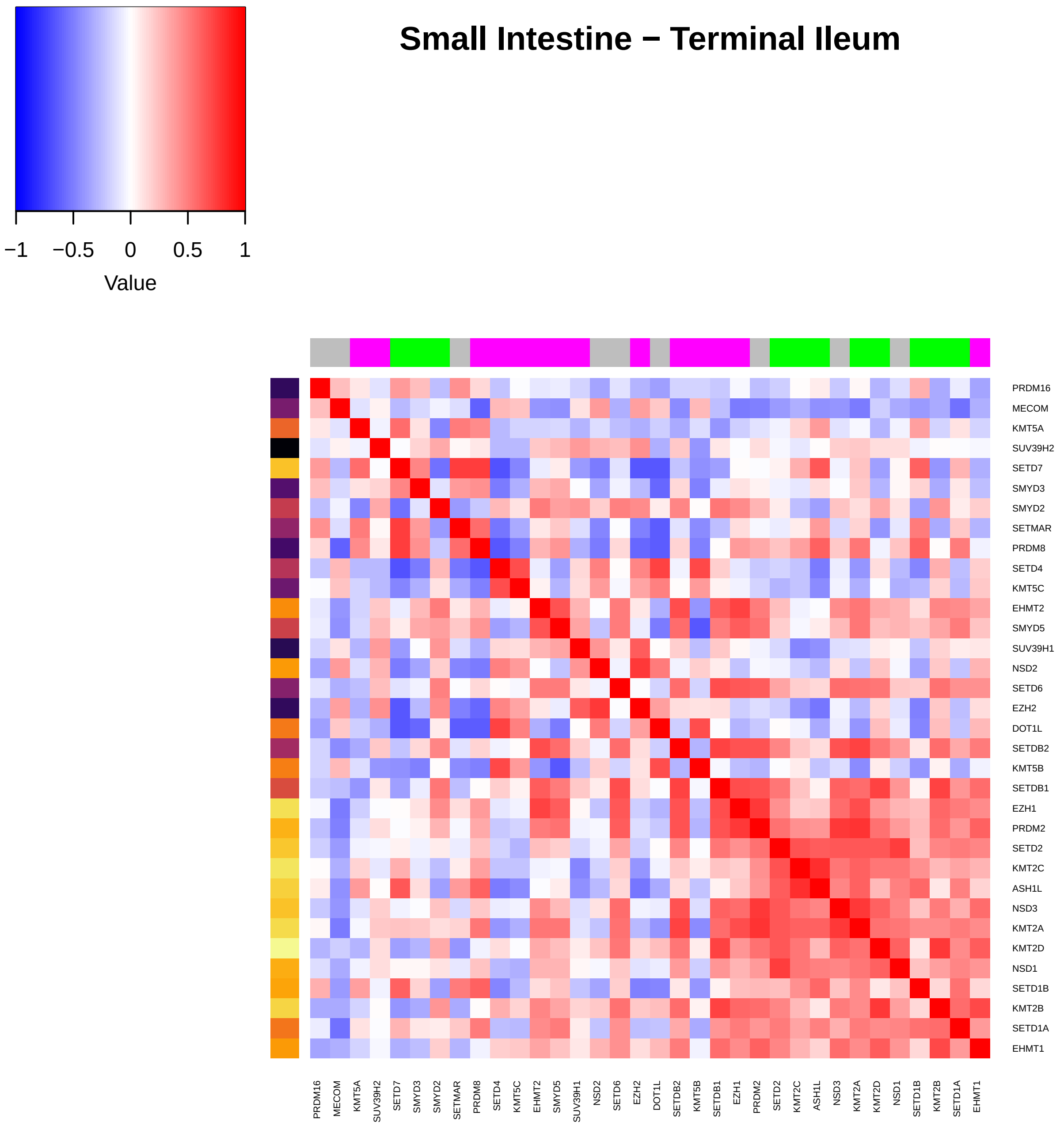
Small Intestine − Terminal Ileum
Value
−1
−0.5
0
0.5
1
PRDM16
MECOM
KMT5A
SUV39H2
SETD7
SMYD3
SMYD2
SETMAR
PRDM8
SETD4
KMT5C
EHMT2
SMYD5
SUV39H1
NSD2
SETD6
EZH2
DOT1L
SETDB2
KMT5B
SETDB1
EZH1
PRDM2
SETD2
KMT2C
ASH1L
NSD3
KMT2A
KMT2D
NSD1
SETD1B
KMT2B
SETD1A
EHMT1

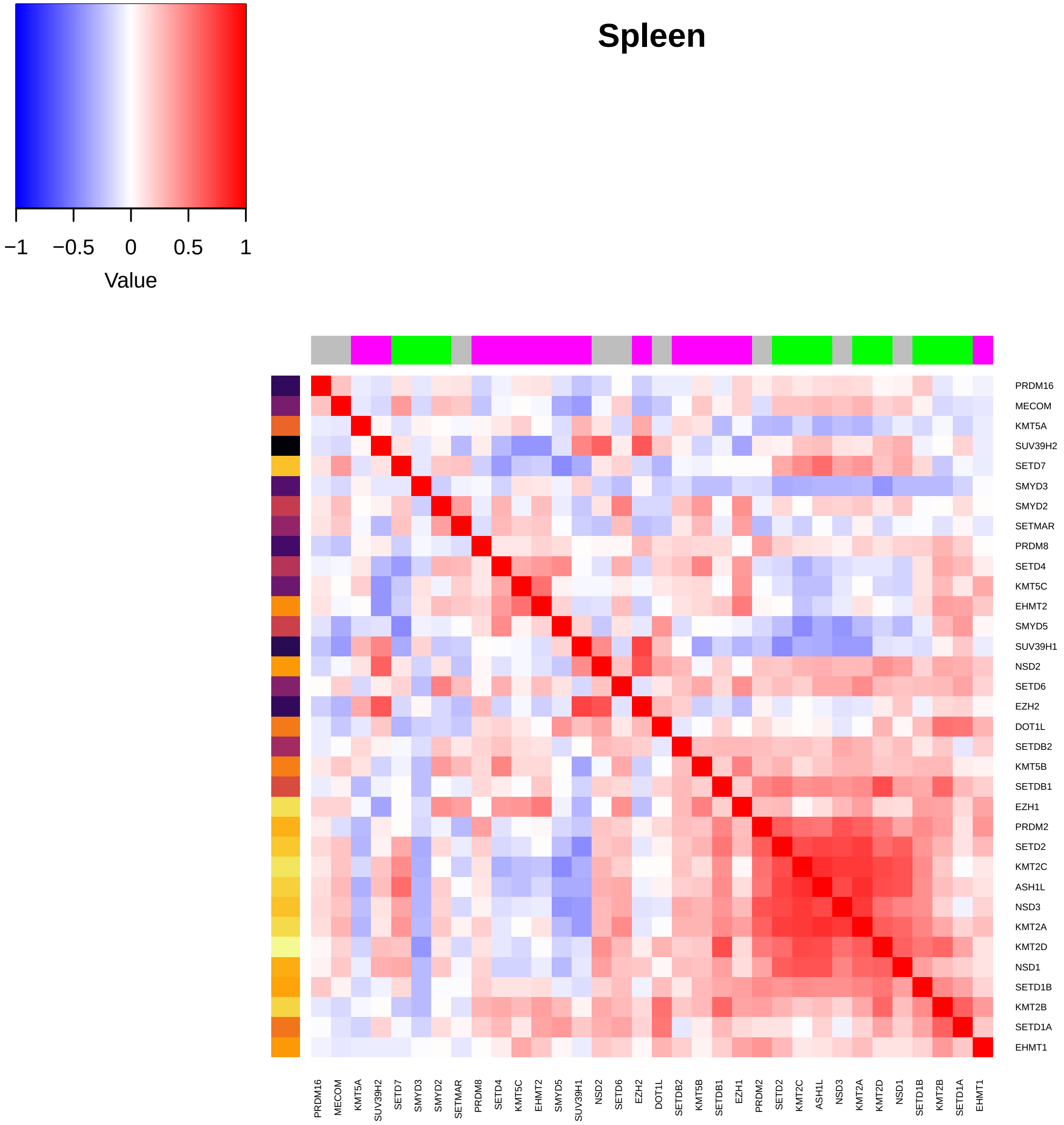
Spleen
Value
−1
−0.5
0
0.5
1
PRDM16
MECOM
KMT5A
SUV39H2
SETD7
SMYD3
SMYD2
SETMAR
PRDM8
SETD4
KMT5C
EHMT2
SMYD5
SUV39H1
NSD2
SETD6
EZH2
DOT1L
SETDB2
KMT5B
SETDB1
EZH1
PRDM2
SETD2
KMT2C
ASH1L
NSD3
KMT2A
KMT2D
NSD1
SETD1B
KMT2B
SETD1A
EHMT1

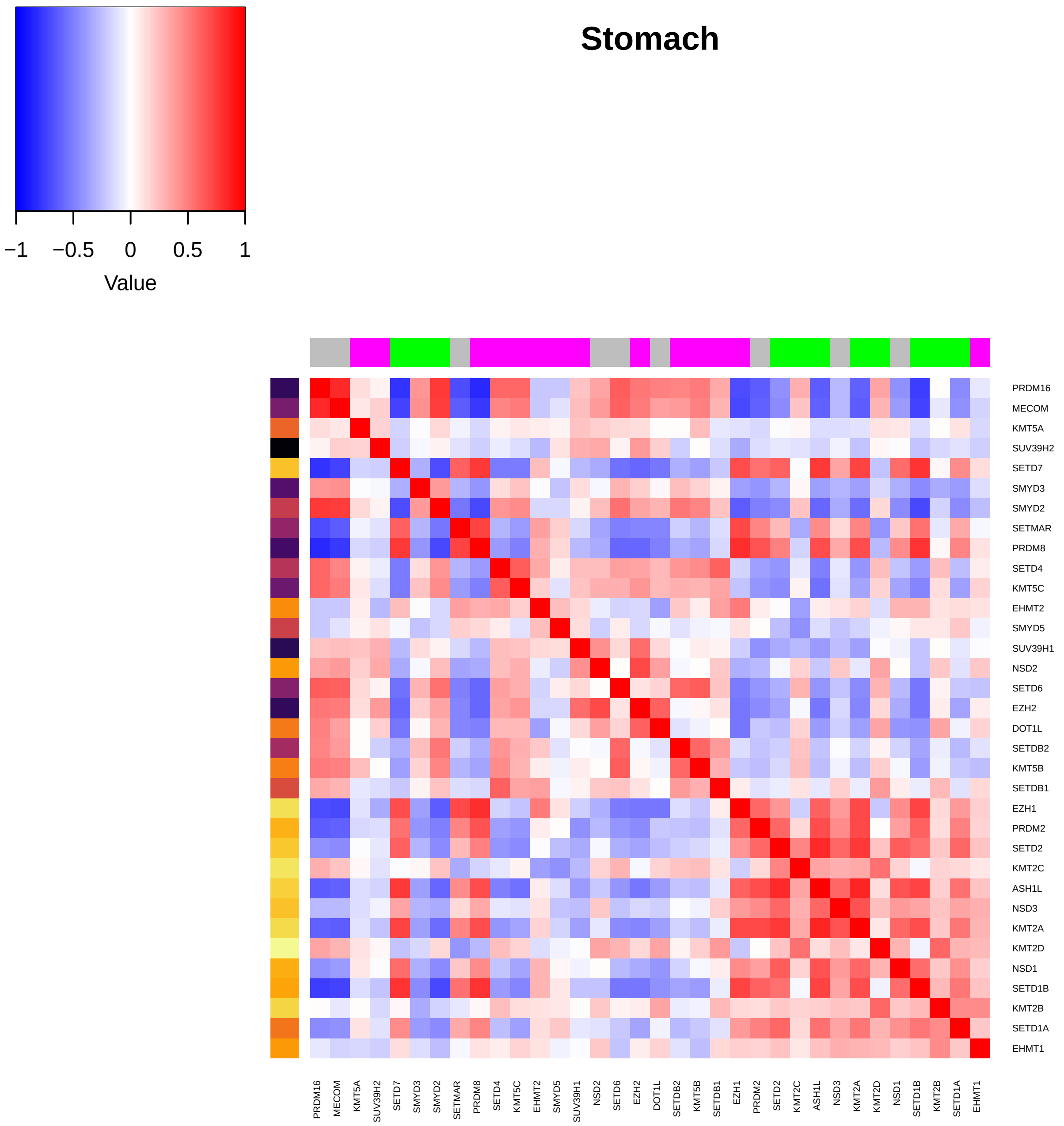
Stomach
−1
−0.5
0
0.5
1
Value
PRDM16
MECOM
KMT5A
SUV39H2
SETD7
SMYD3
SMYD2
SETMAR
PRDM8
SETD4
KMT5C
EHMT2
SMYD5
SUV39H1
NSD2
SETD6
EZH2
DOT1L
SETDB2
KMT5B
SETDB1
EZH1
PRDM2
SETD2
KMT2C
ASH1L
NSD3
KMT2A
KMT2D
NSD1
SETD1B
KMT2B
SETD1A
EHMT1

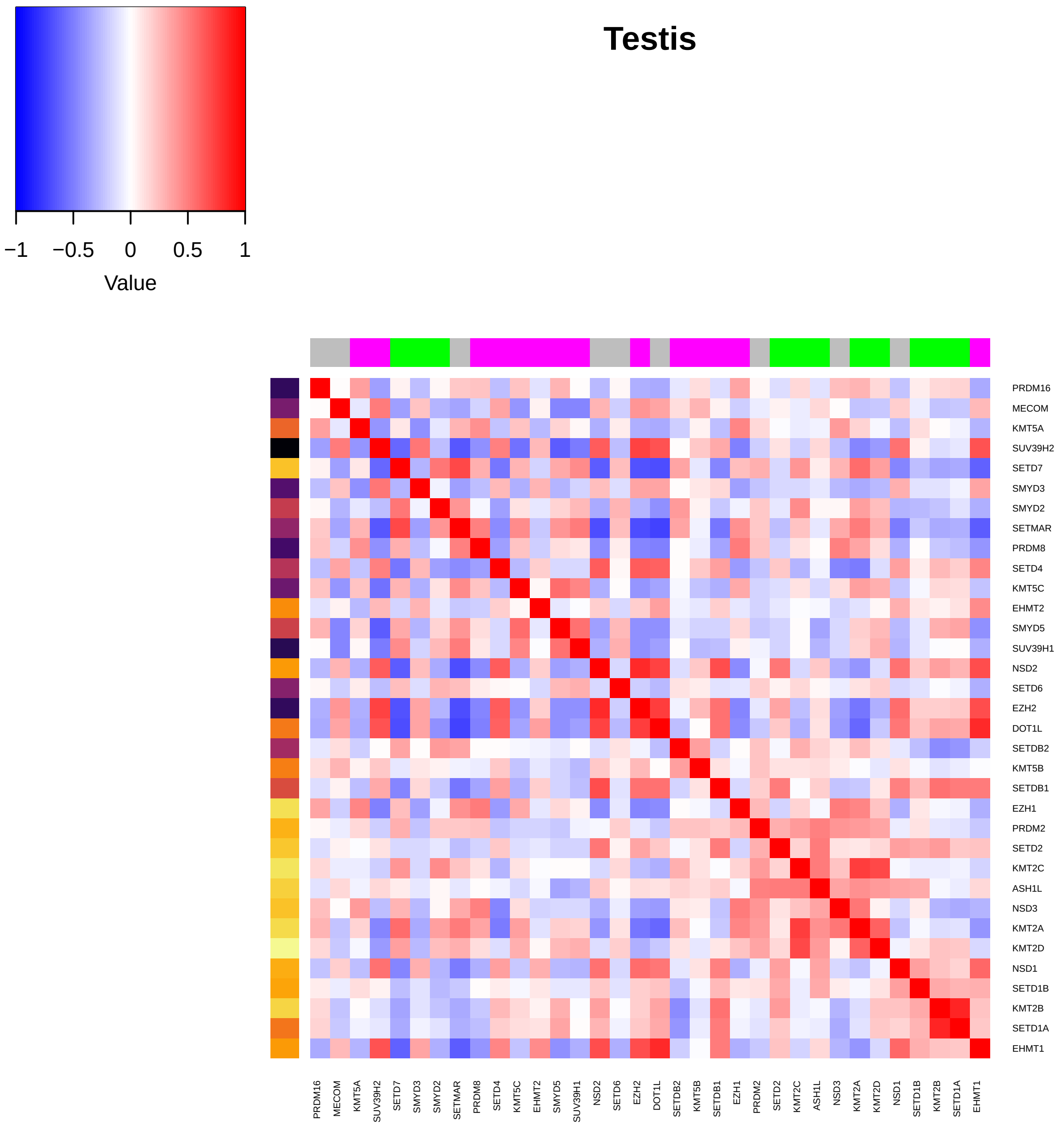
Testis
−1
−0.5
0
0.5
1
Value
PRDM16
MECOM
KMT5A
SUV39H2
SETD7
SMYD3
SMYD2
SETMAR
PRDM8
SETD4
KMT5C
EHMT2
SMYD5
SUV39H1
NSD2
SETD6
EZH2
DOT1L
SETDB2
KMT5B
SETDB1
EZH1
PRDM2
SETD2
KMT2C
ASH1L
NSD3
KMT2A
KMT2D
NSD1
SETD1B
KMT2B
SETD1A
EHMT1

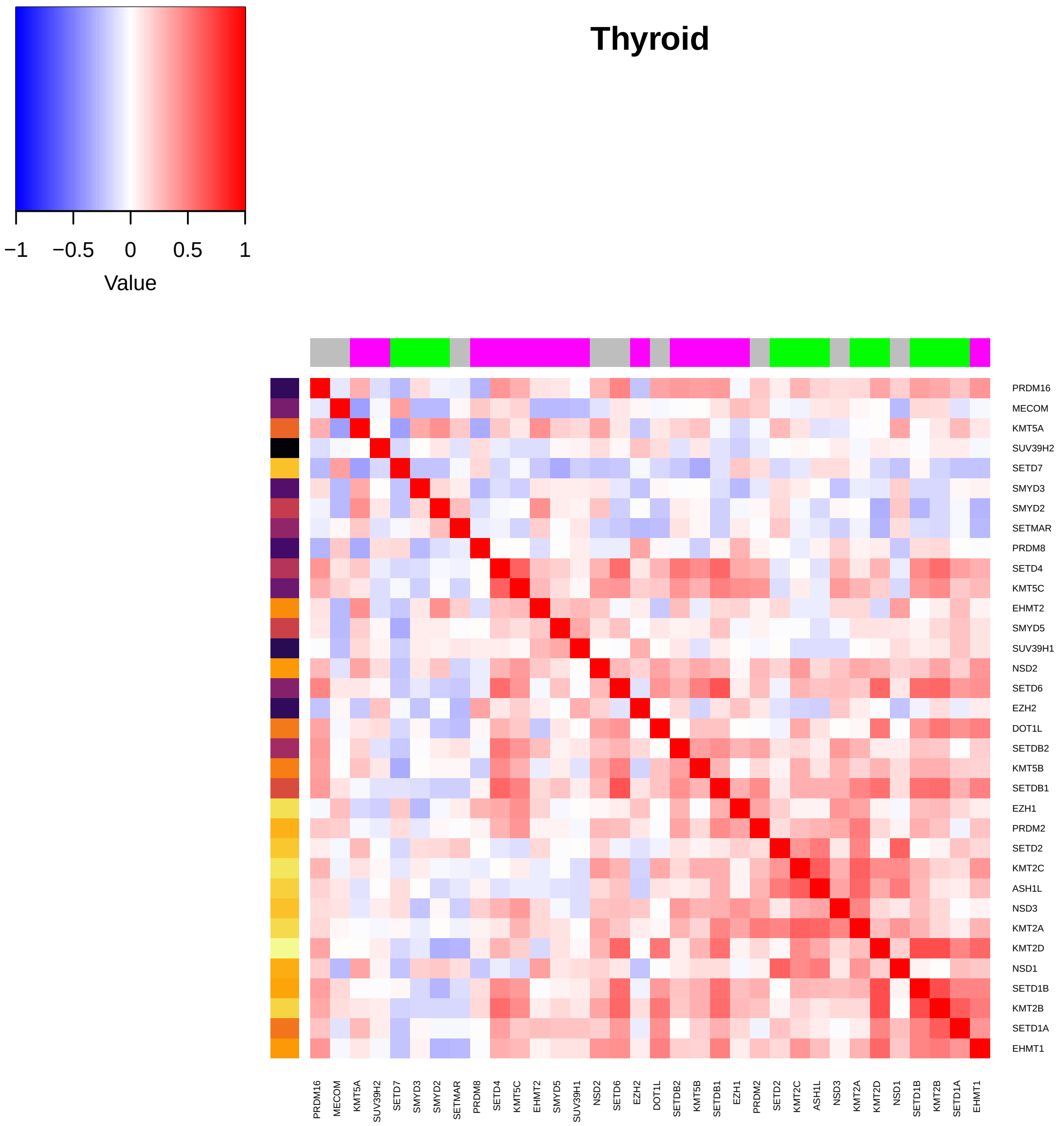

Thyroid
−1
−0.5
0
0.5
1
Value
PRDM16
MECOM
KMT5A
SUV39H2
SETD7
SMYD3
SMYD2
SETMAR
PRDM8
SETD4
KMT5C
EHMT2
SMYD5
SUV39H1
NSD2
SETD6
EZH2
DOT1L
SETDB2
KMT5B
SETDB1
EZH1
PRDM2
SETD2
KMT2C
ASH1L
NSD3
KMT2A
KMT2D
NSD1
SETD1B
KMT2B
SETD1A
EHMT1

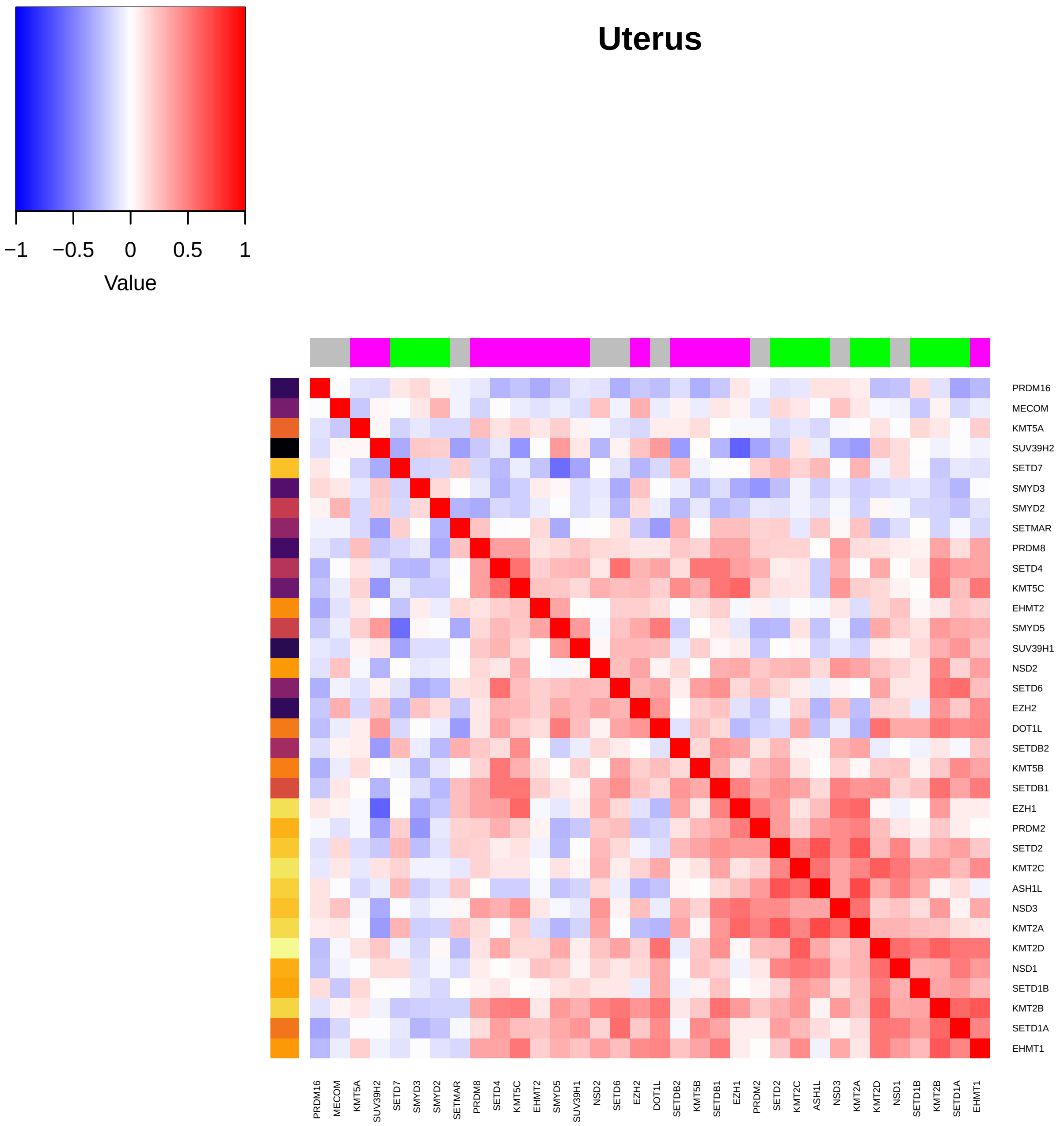
Uterus
Value
−1
−0.5
0
0.5
1
PRDM16
MECOM
KMT5A
SUV39H2
SETD7
SMYD3
SMYD2
SETMAR
PRDM8
SETD4
KMT5C
EHMT2
SMYD5
SUV39H1
NSD2
SETD6
EZH2
DOT1L
SETDB2
KMT5B
SETDB1
EZH1
PRDM2
SETD2
KMT2C
ASH1L
NSD3
KMT2A
KMT2D
NSD1
SETD1B
KMT2B
SETD1A
EHMT1

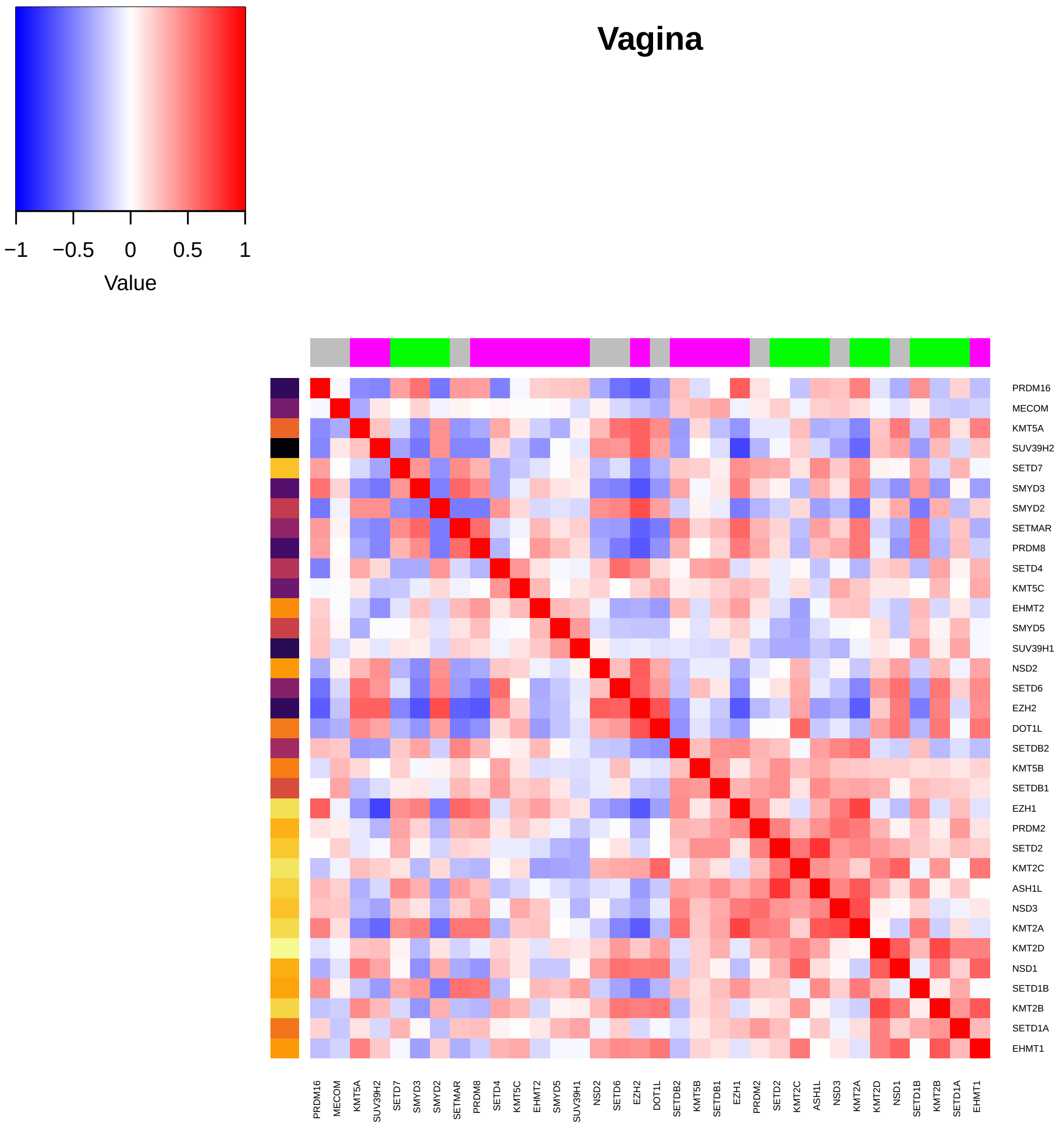
Vagina
Value
−1
−0.5
0
0.5
1
PRDM16
MECOM
KMT5A
SUV39H2
SETD7
SMYD3
SMYD2
SETMAR
PRDM8
SETD4
KMT5C
EHMT2
SMYD5
SUV39H1
NSD2
SETD6
EZH2
DOT1L
SETDB2
KMT5B
SETDB1
EZH1
PRDM2
SETD2
KMT2C
ASH1L
NSD3
KMT2A
KMT2D
NSD1
SETD1B
KMT2B
SETD1A
EHMT1

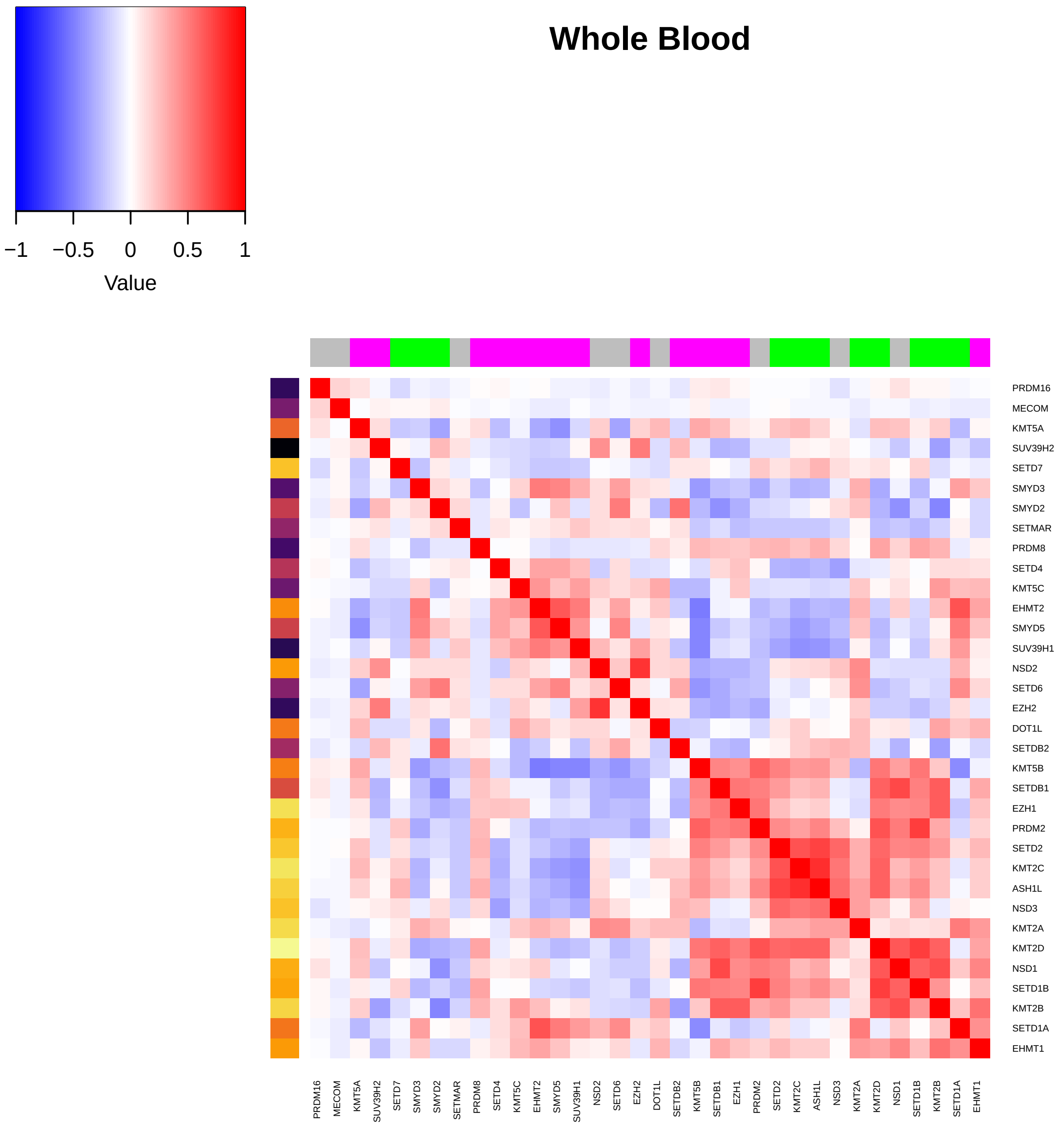
Value
−1
−0.5
0
0.5
1
Whole Blood
PRDM16
MECOM
KMT5A
SUV39H2
SETD7
SMYD3
SMYD2
SETMAR
PRDM8
SETD4
KMT5C
EHMT2
SMYD5
SUV39H1
NSD2
SETD6
EZH2
DOT1L
SETDB2
KMT5B
SETDB1
EZH1
PRDM2
SETD2
KMT2C
ASH1L
NSD3
KMT2A
KMT2D
NSD1
SETD1B
KMT2B
SETD1A
EHMT1
