# Supplementary material for: Histone methyltransferase activity affects metabolism in human cells independently of transcriptional regulation
Source: PLoS Biol. 2023 Oct 26;21(10):e3002354. doi: 10.1371/journal.pbio.3002354 (PMC10602318; doi:10.1371/journal.pbio.3002354)
Supplement: S7 File — (PDF) [file pbio.3002354.s029.pdf]

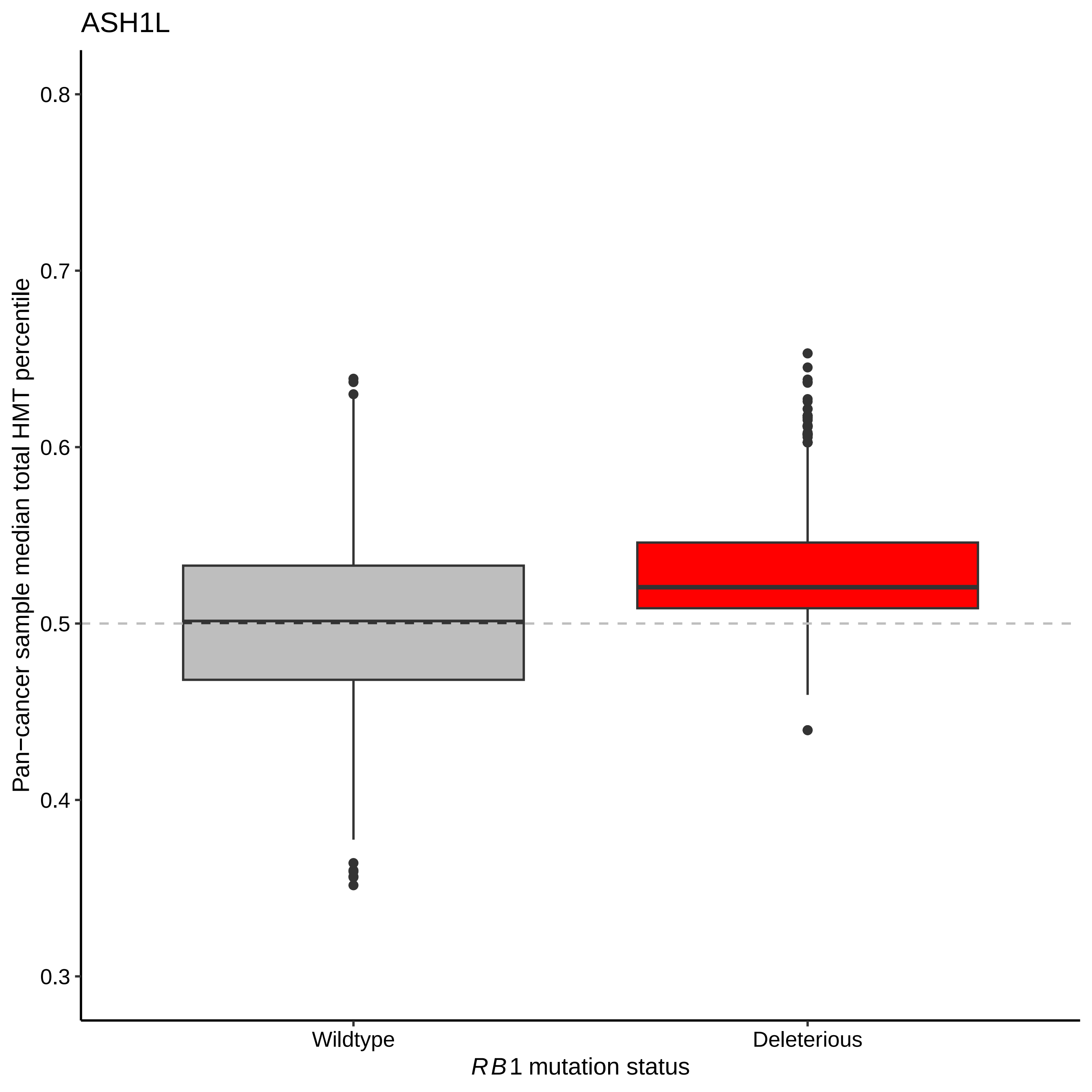
ASH1L
0.8
0.7
0.6
0.5
0.4
0.3
Pan−cancer sample median total HMT percentile
Wildtype
Deleterious
RB1 mutation status

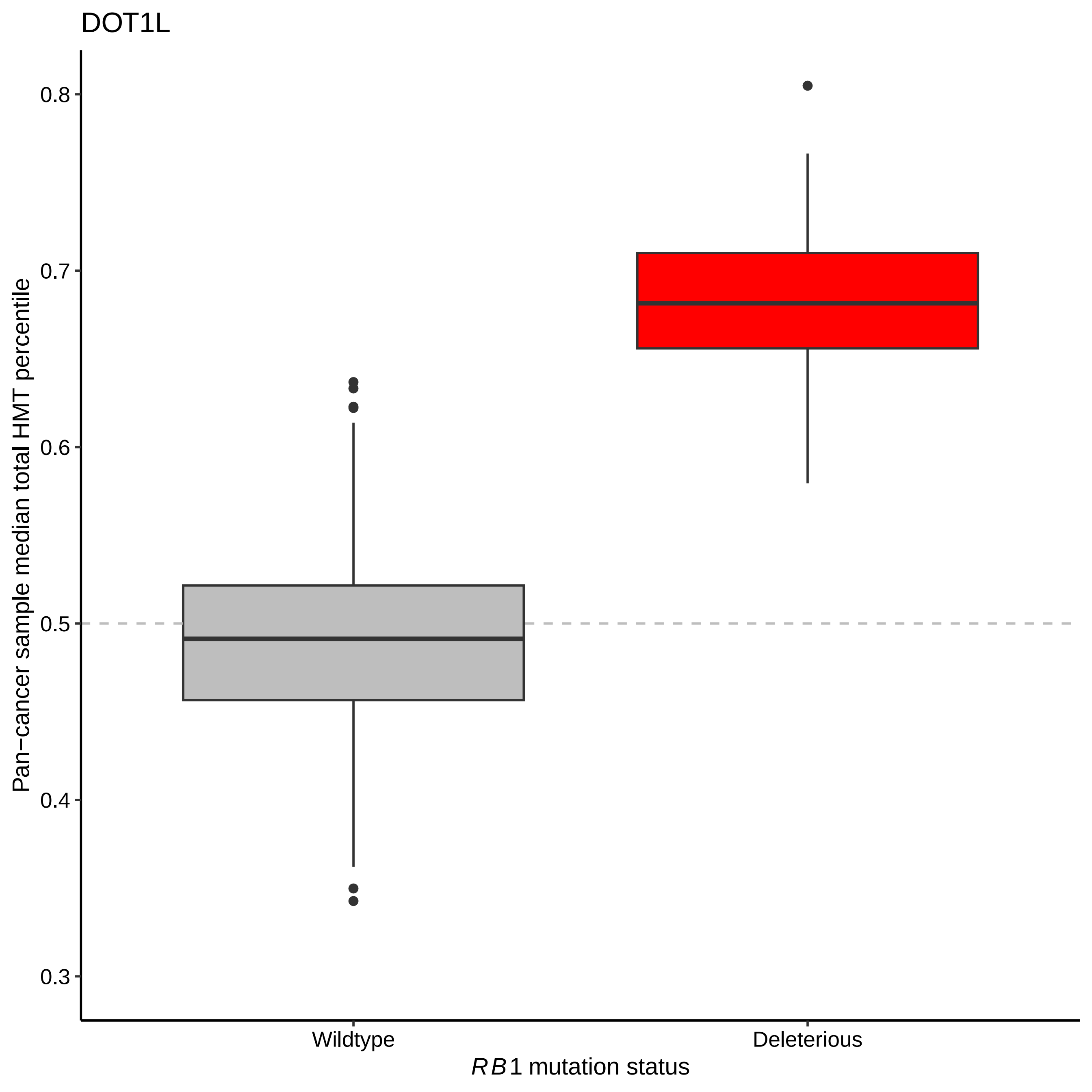
DOT1L
Pan−cancer sample median total HMT percentile
0.8
0.7
0.6
0.5
0.4
0.3
Wildtype
Deleterious
RB1 mutation status

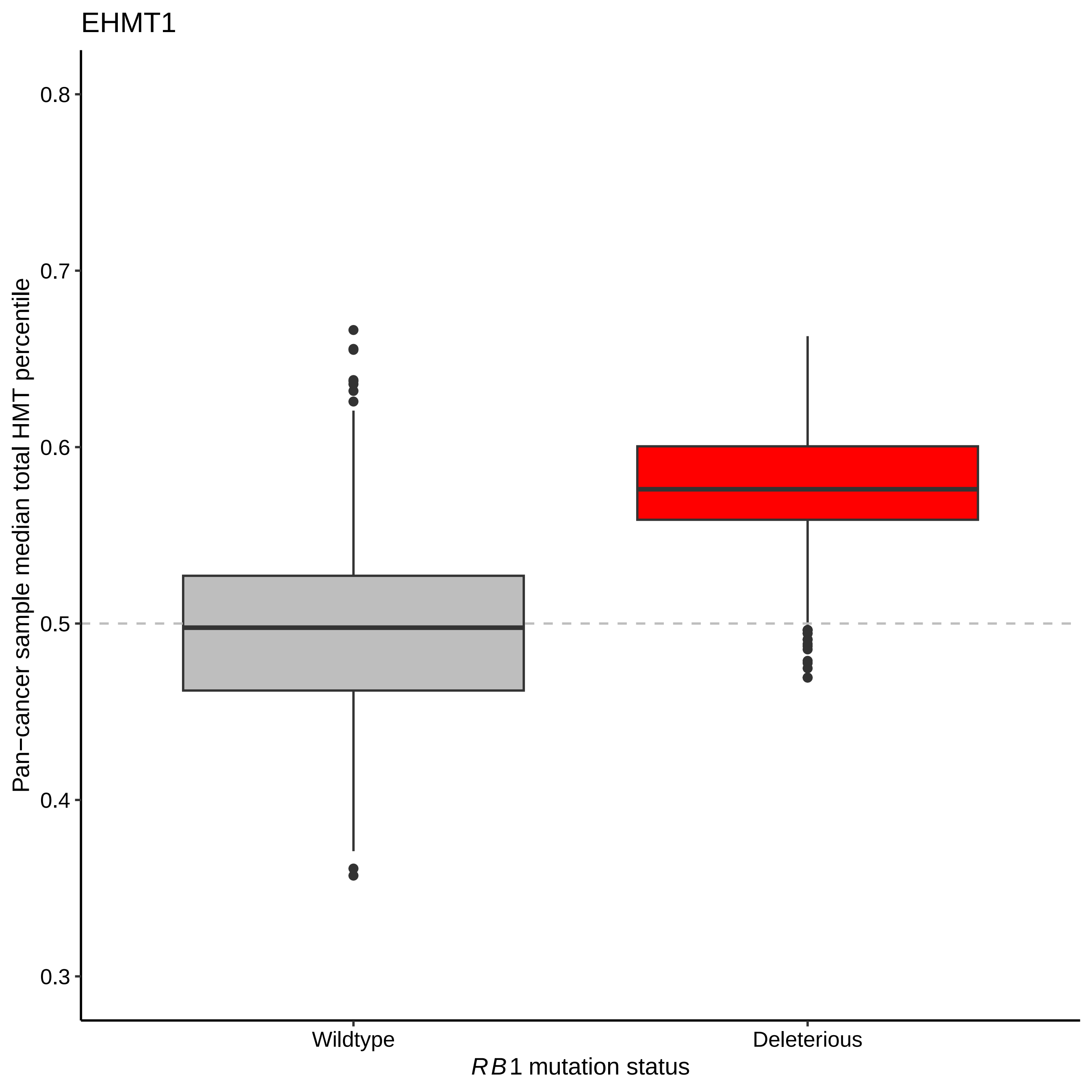

EHMT1
Pan−cancer sample median total HMT percentile
0.8
0.7
0.6
0.5
0.4
0.3
Wildtype
Deleterious
RB1 mutation status

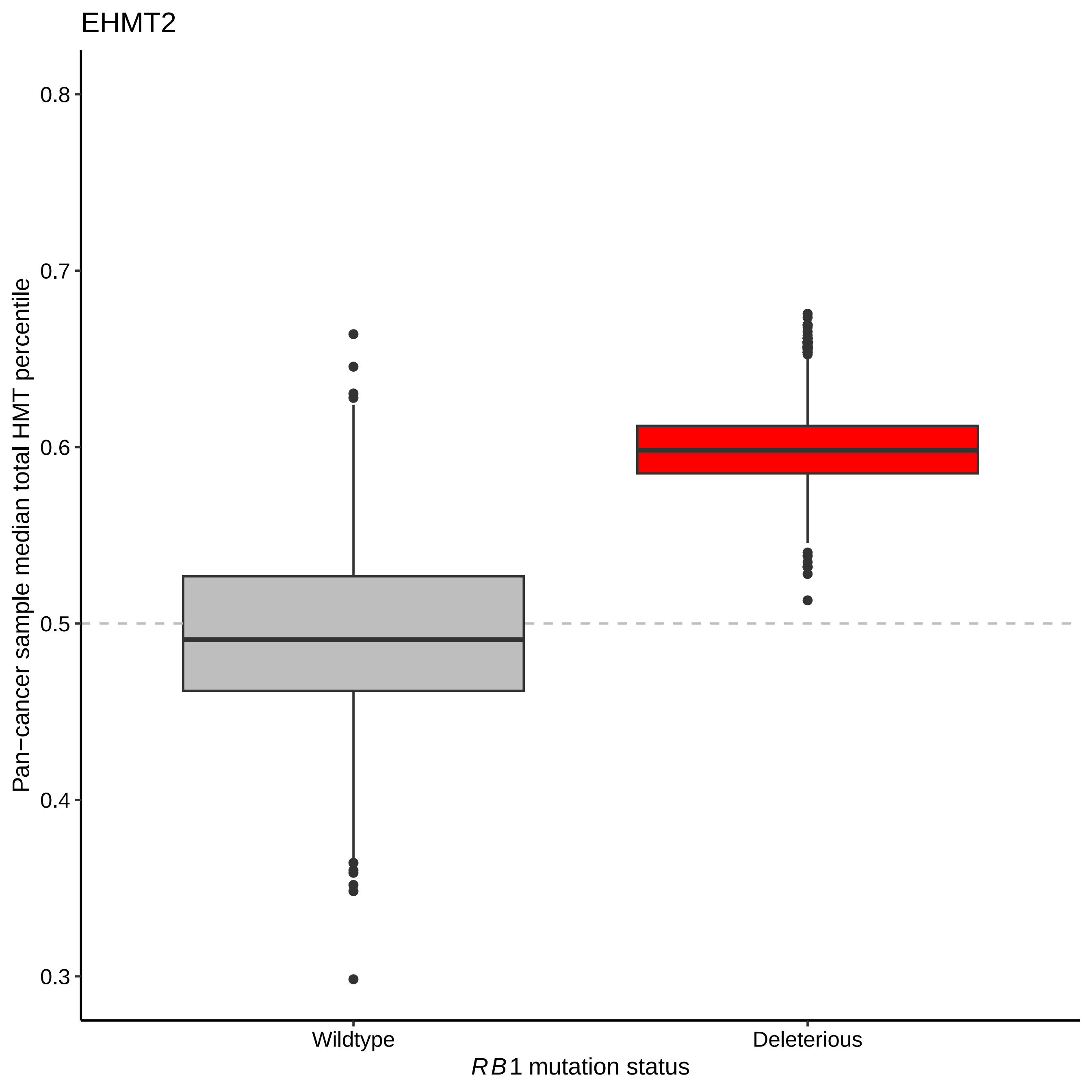
EHMT2
Pan−cancer sample median total HMT percentile
0.8
0.7
0.6
0.5
0.4
0.3
Wildtype
Deleterious
RB1 mutation status

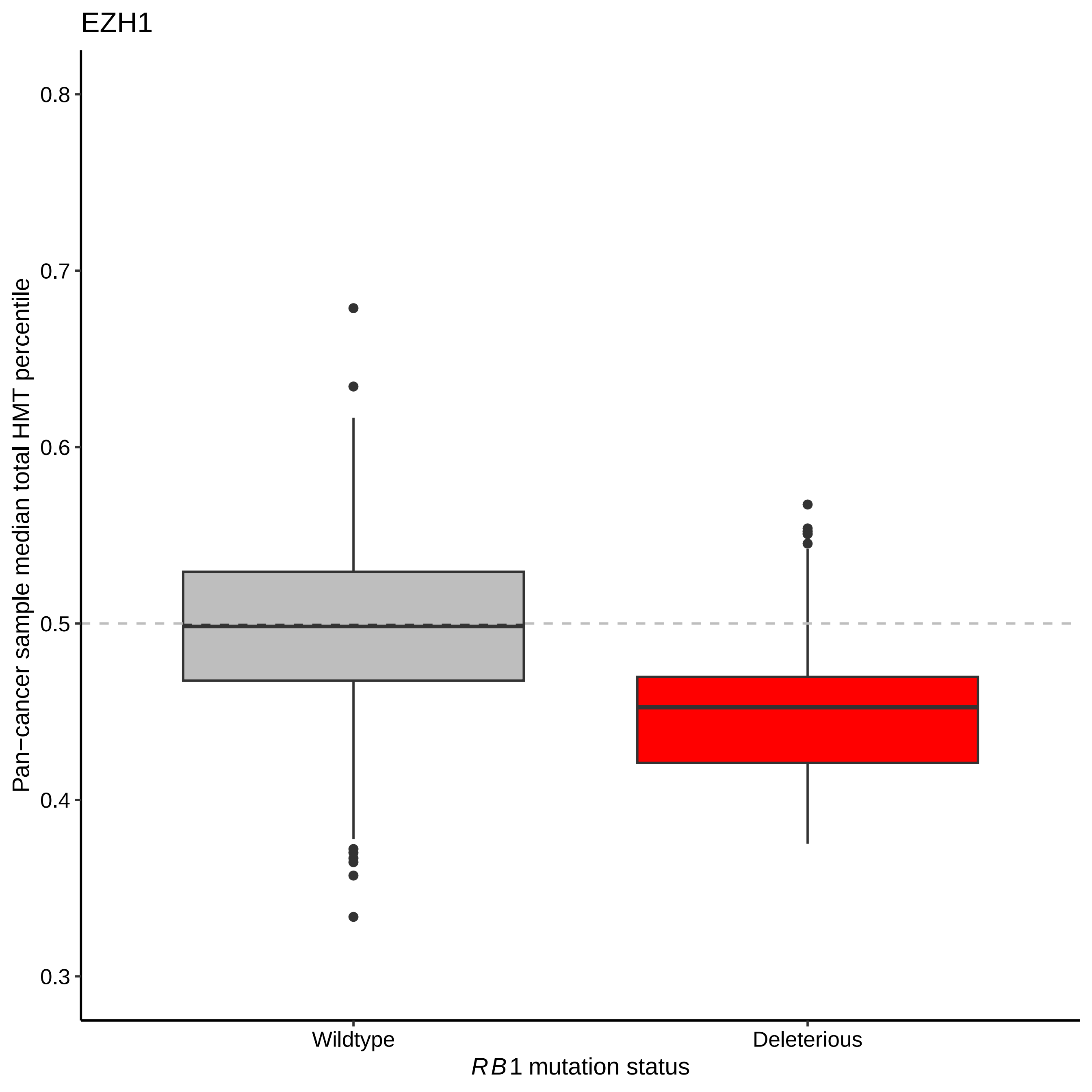

EZH1
0.8
0.7
0.6
0.5
0.4
0.3
Pan−cancer sample median total HMT percentile
Wildtype
Deleterious
RB1 mutation status

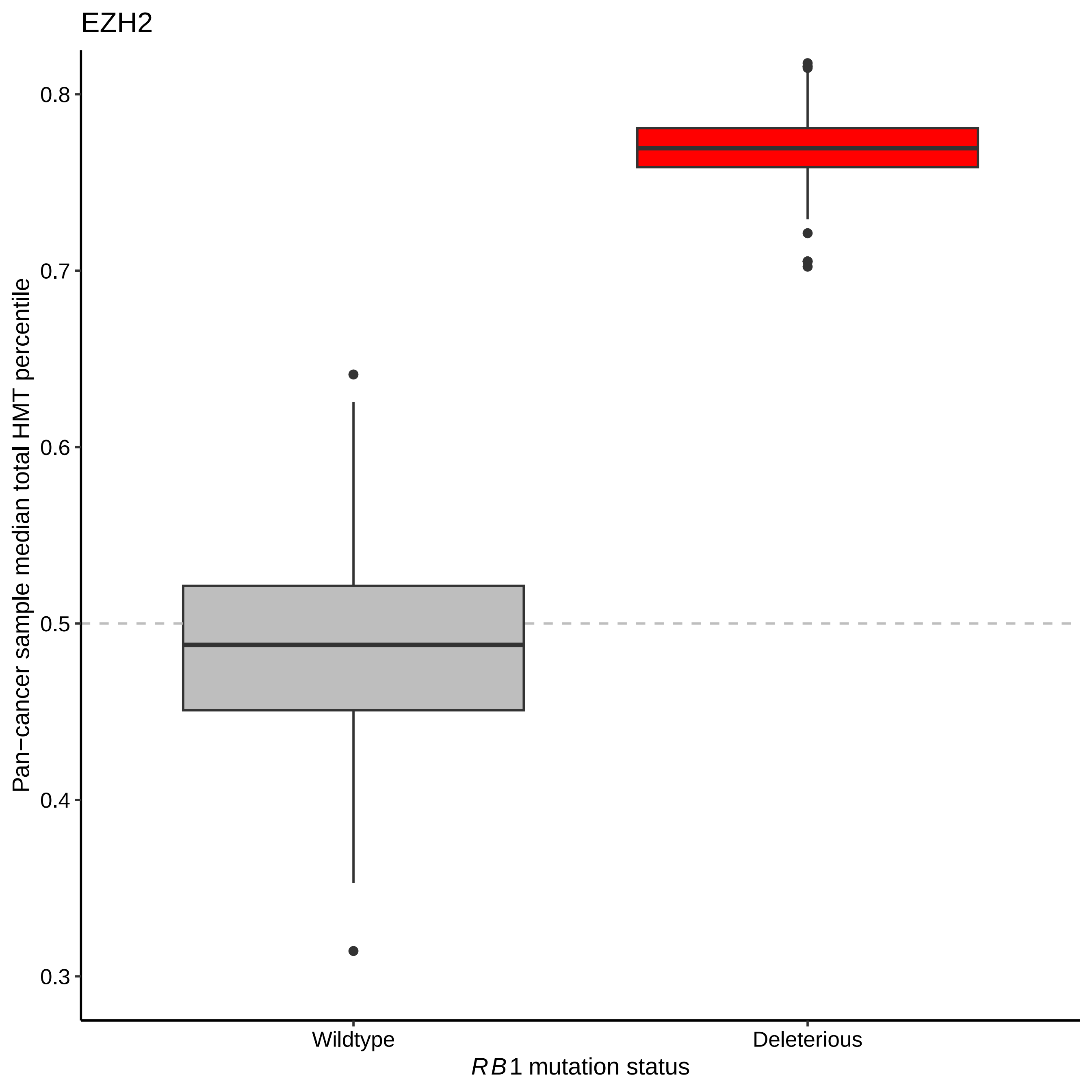
EZH2
Pan−cancer sample median total HMT percentile
0.8
0.7
0.6
0.5
0.4
0.3
Wildtype
Deleterious
RB1 mutation status

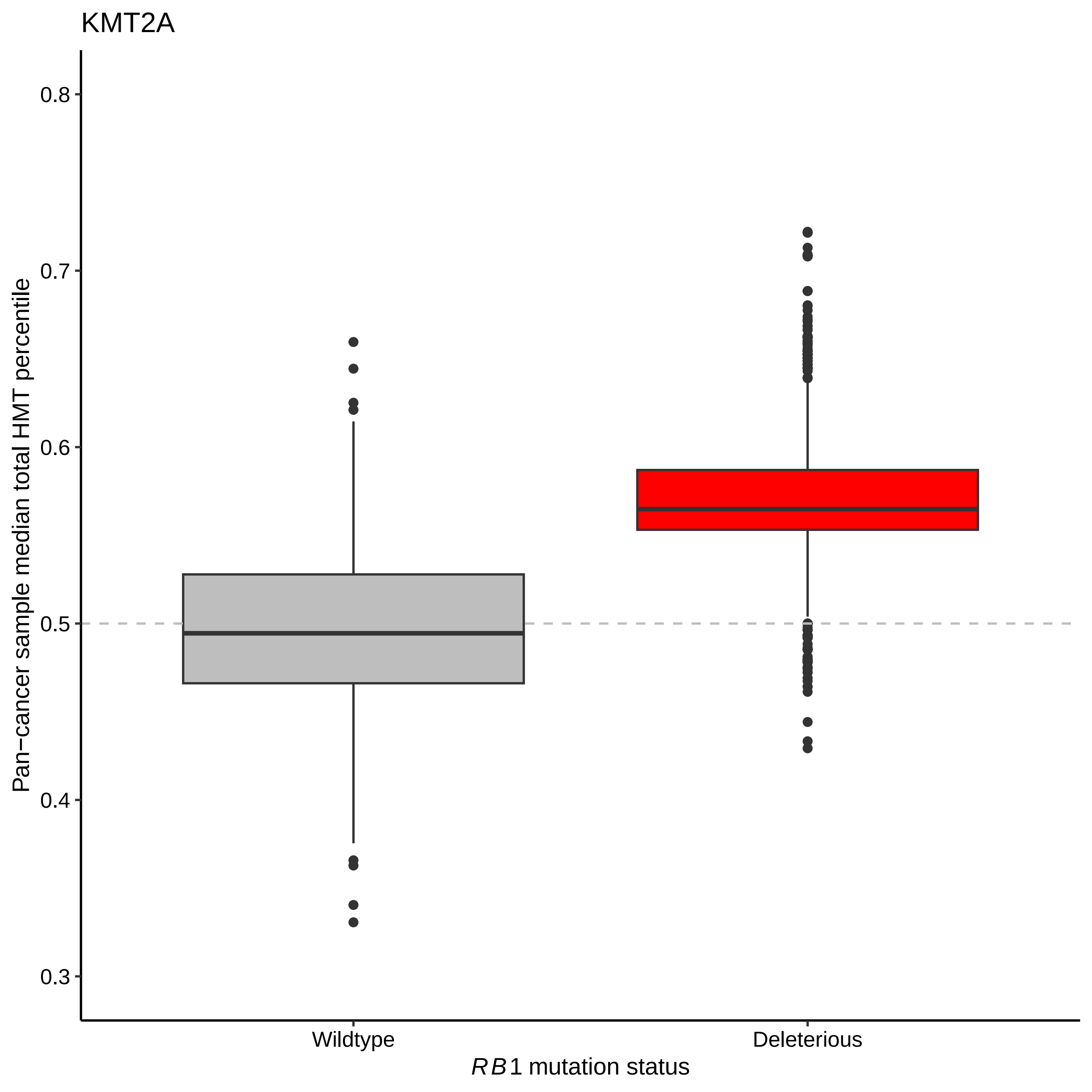
KMT2A
0.8
0.7
0.6
0.5
0.4
0.3
Pan−cancer sample median total HMT percentile
Wildtype
Deleterious
RB1 mutation status

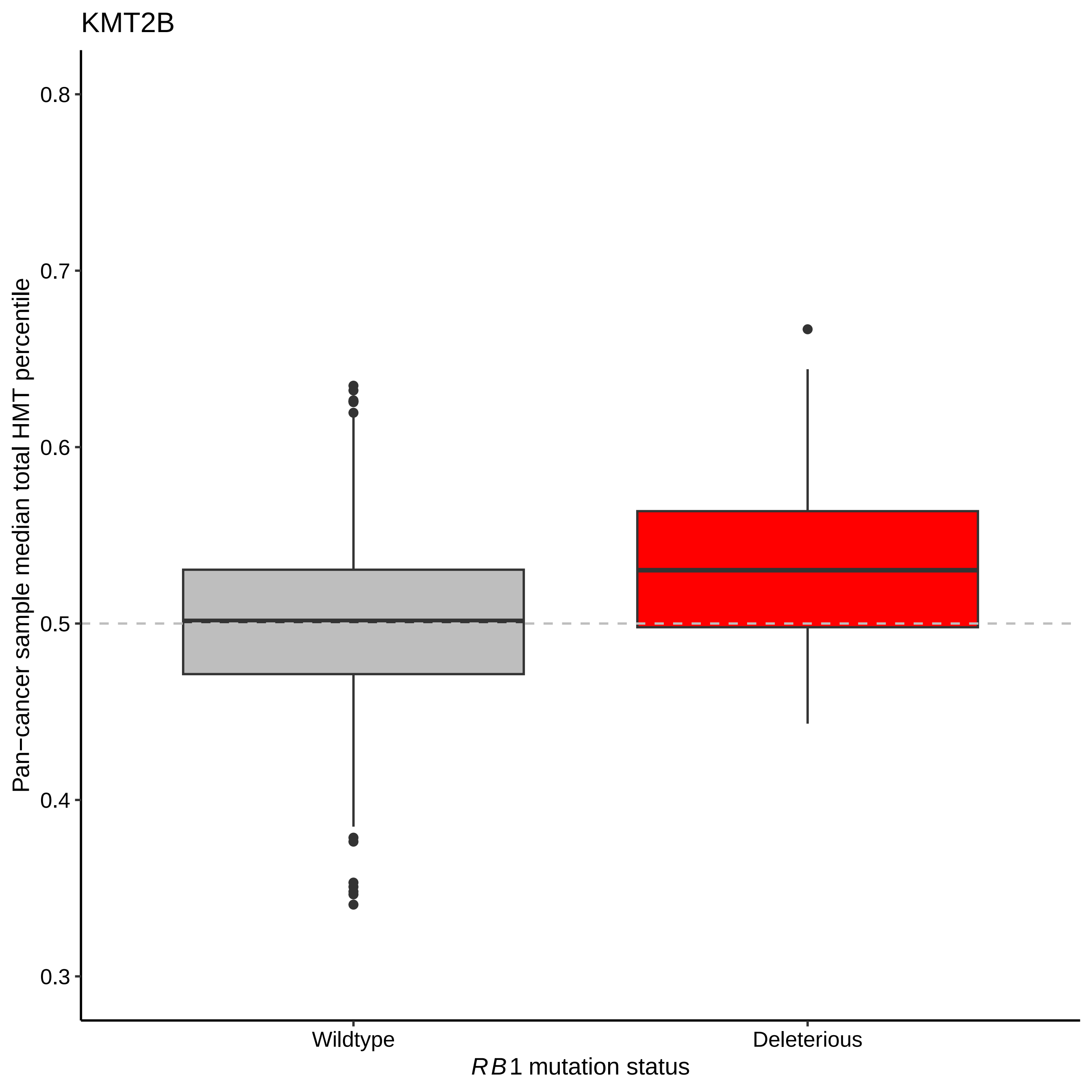
KMT2B
Pan−cancer sample median total HMT percentile
0.8
0.7
0.6
0.5
0.4
0.3
Wildtype
Deleterious
RB1 mutation status

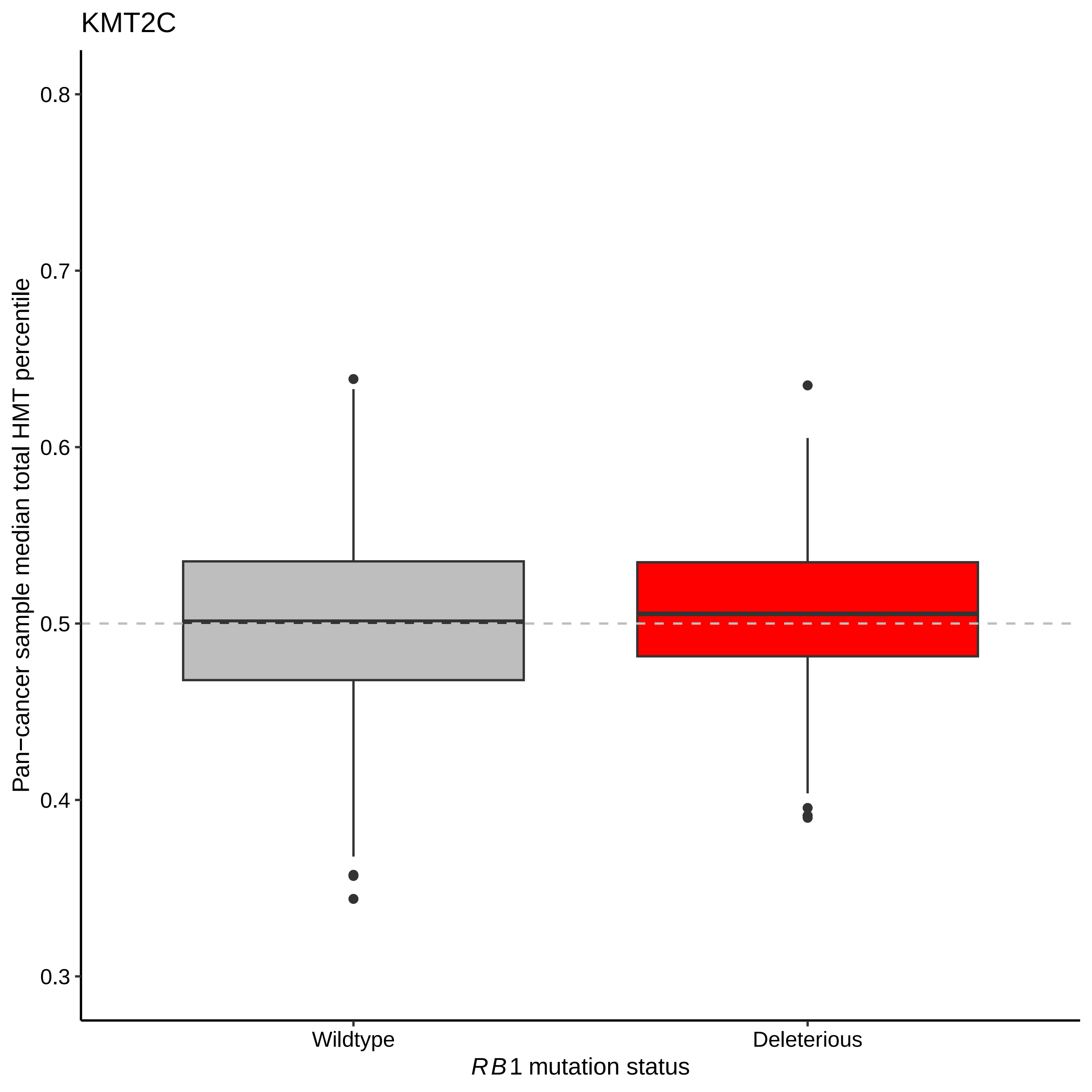
KMT2C
Pan−cancer sample median total HMT percentile
0.8
0.7
0.6
0.5
0.4
0.3
Wildtype
Deleterious
RB1 mutation status

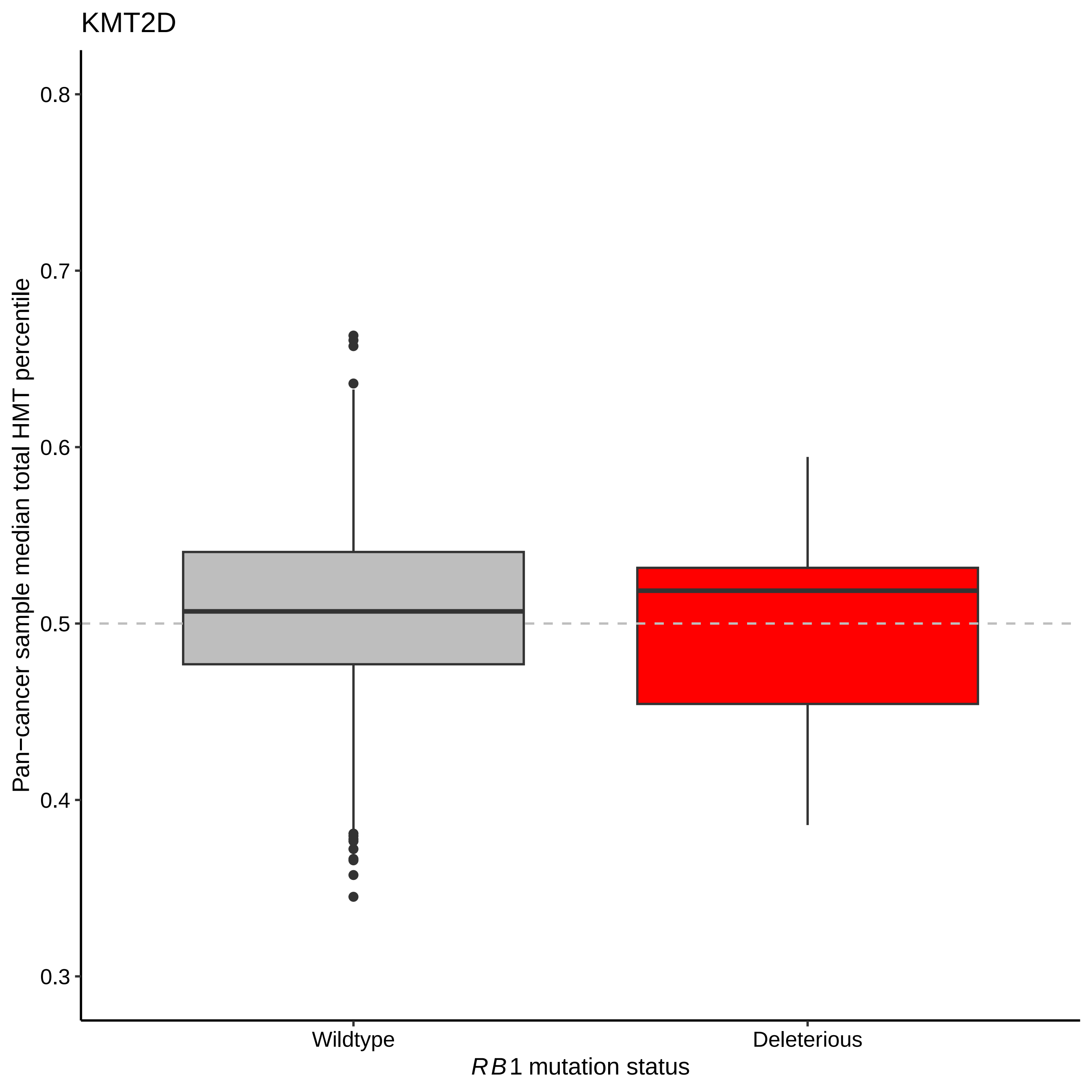
KMT2D
Pan−cancer sample median total HMT percentile
0.8
0.7
0.6
0.5
0.4
0.3
Wildtype
Deleterious
RB1 mutation status

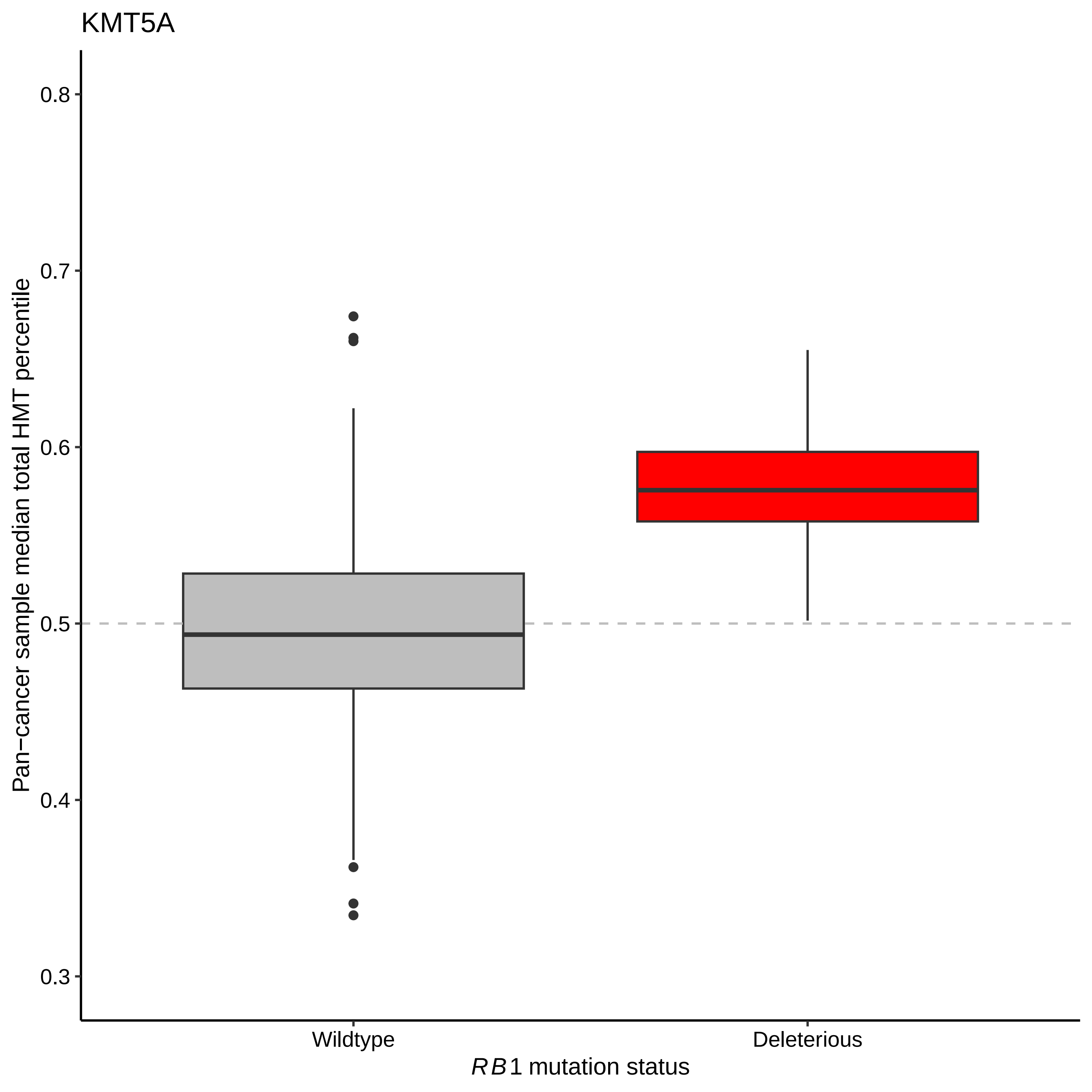
KMT5A
Pan−cancer sample median total HMT percentile
0.8
0.7
0.6
0.5
0.4
0.3
Wildtype
Deleterious
RB1 mutation status

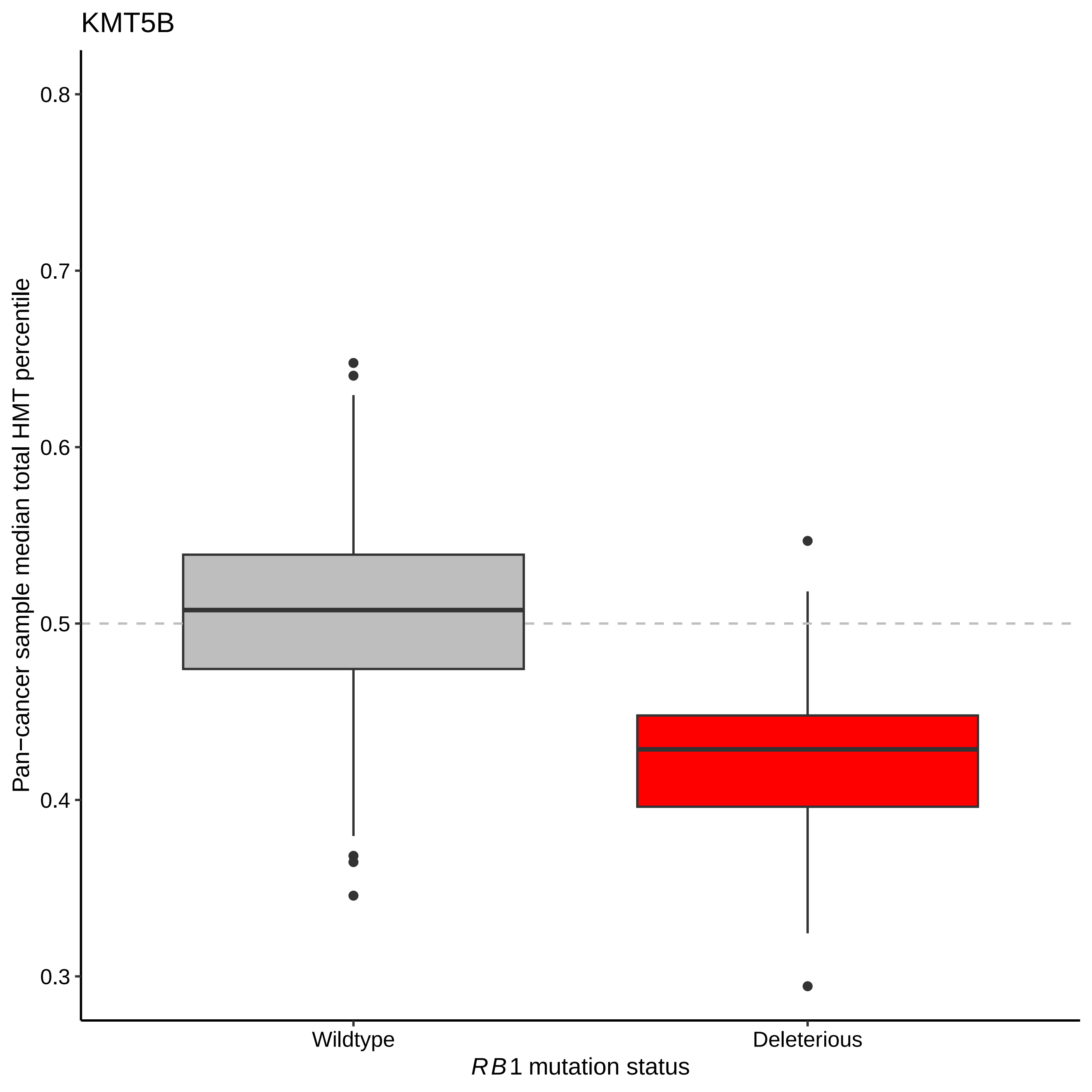
KMT5B
0.8
0.7
0.6
0.5
0.4
0.3
Pan−cancer sample median total HMT percentile
Wildtype
Deleterious
RB1 mutation status

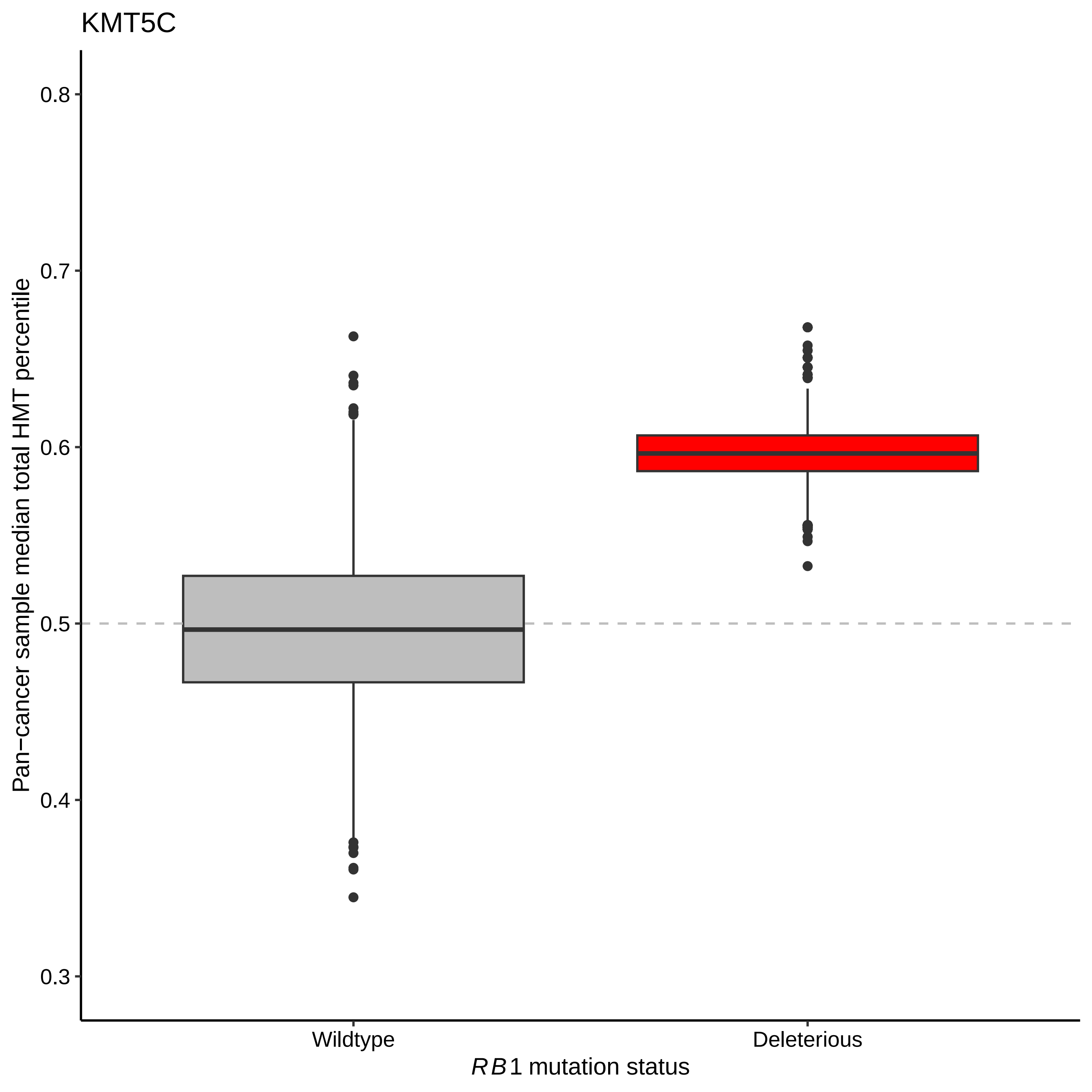

KMT5C
Pan−cancer sample median total HMT percentile
0.3
0.4
0.5
0.6
0.7
0.8
Wildtype
Deleterious
RB1 mutation status

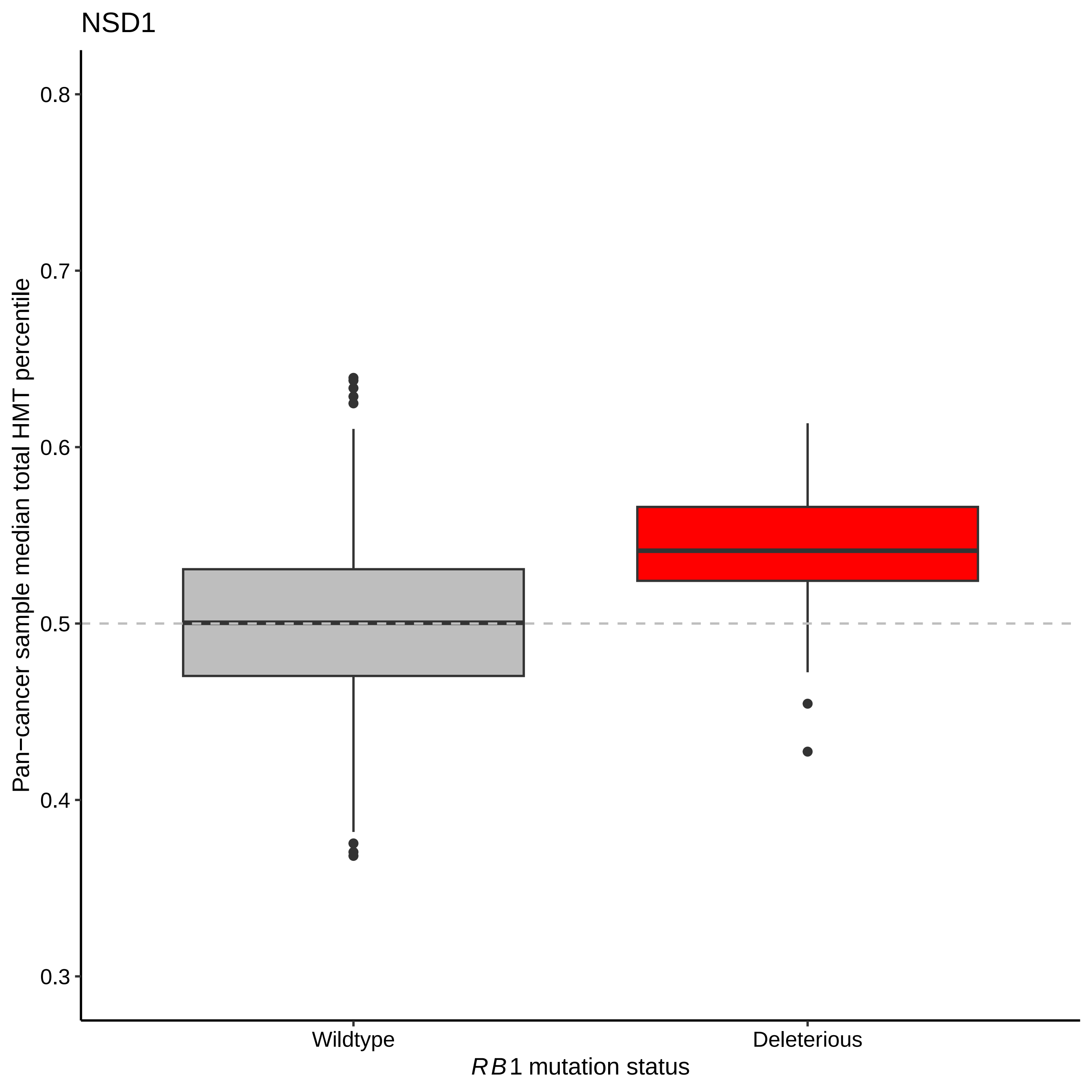
NSD1
Pan−cancer sample median total HMT percentile
0.8
0.7
0.6
0.5
0.4
0.3
Wildtype
Deleterious
RB1 mutation status

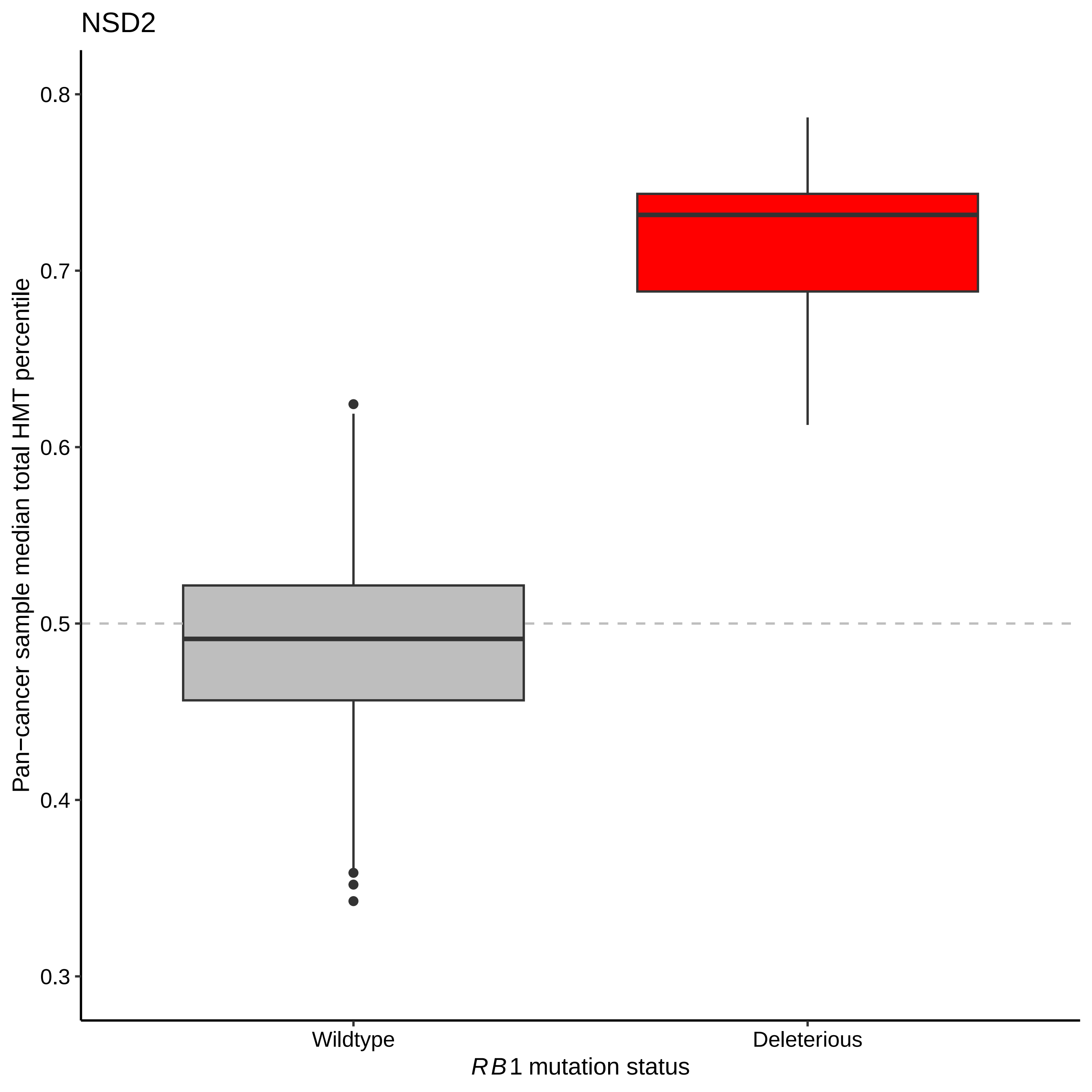
NSD2
Pan−cancer sample median total HMT percentile
0.8
0.7
0.6
0.5
0.4
0.3
Wildtype
Deleterious
RB1 mutation status

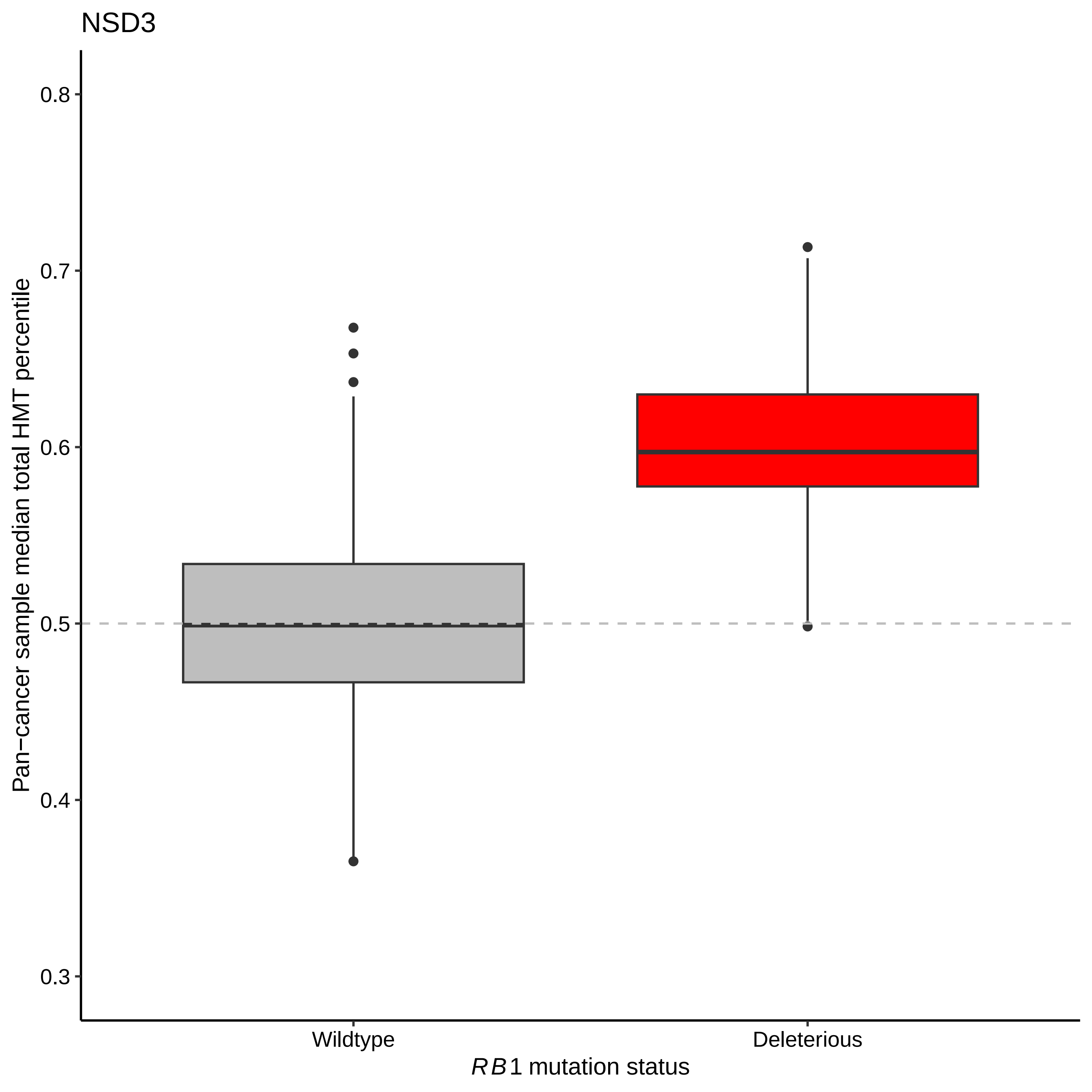
NSD3
Pan−cancer sample median total HMT percentile
0.8
0.7
0.6
0.5
0.4
0.3
Wildtype
Deleterious
RB1 mutation status

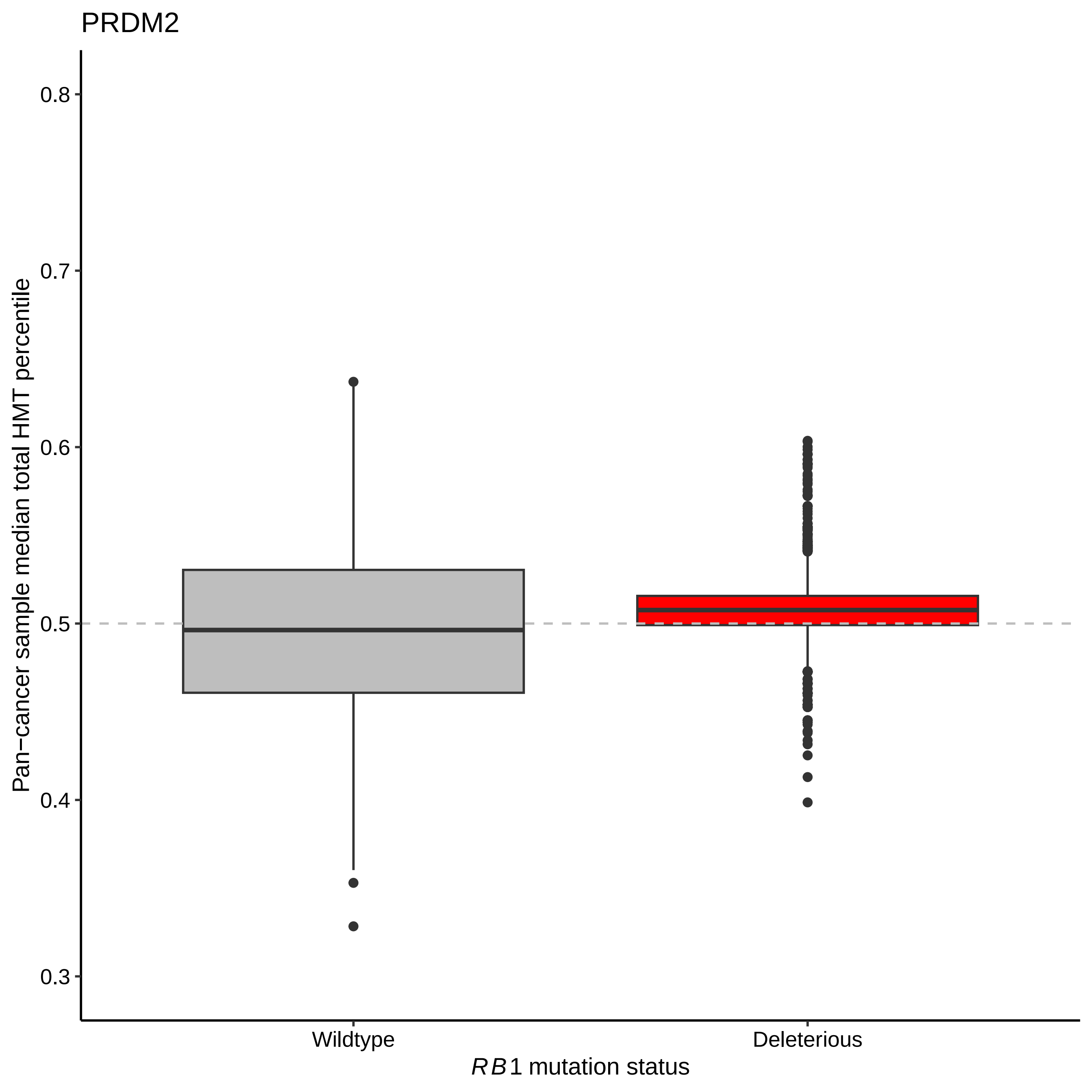
PRDM2
Pan−cancer sample median total HMT percentile
0.8
0.7
0.6
0.5
0.4
0.3
Wildtype
Deleterious
RB1 mutation status

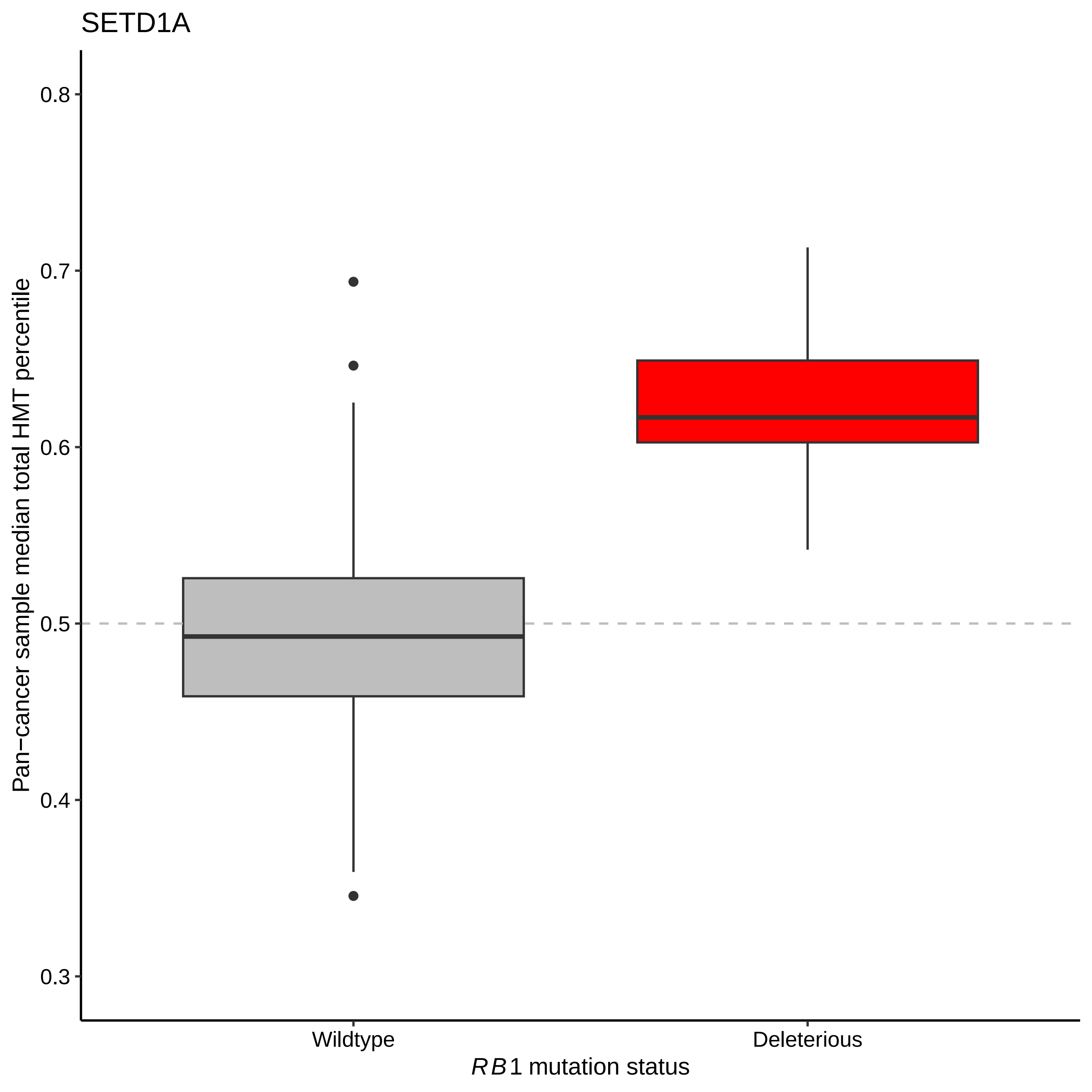
SETD1A
0.8
0.7
0.6
0.5
0.4
0.3
Pan−cancer sample median total HMT percentile
Wildtype
Deleterious
RB1 mutation status

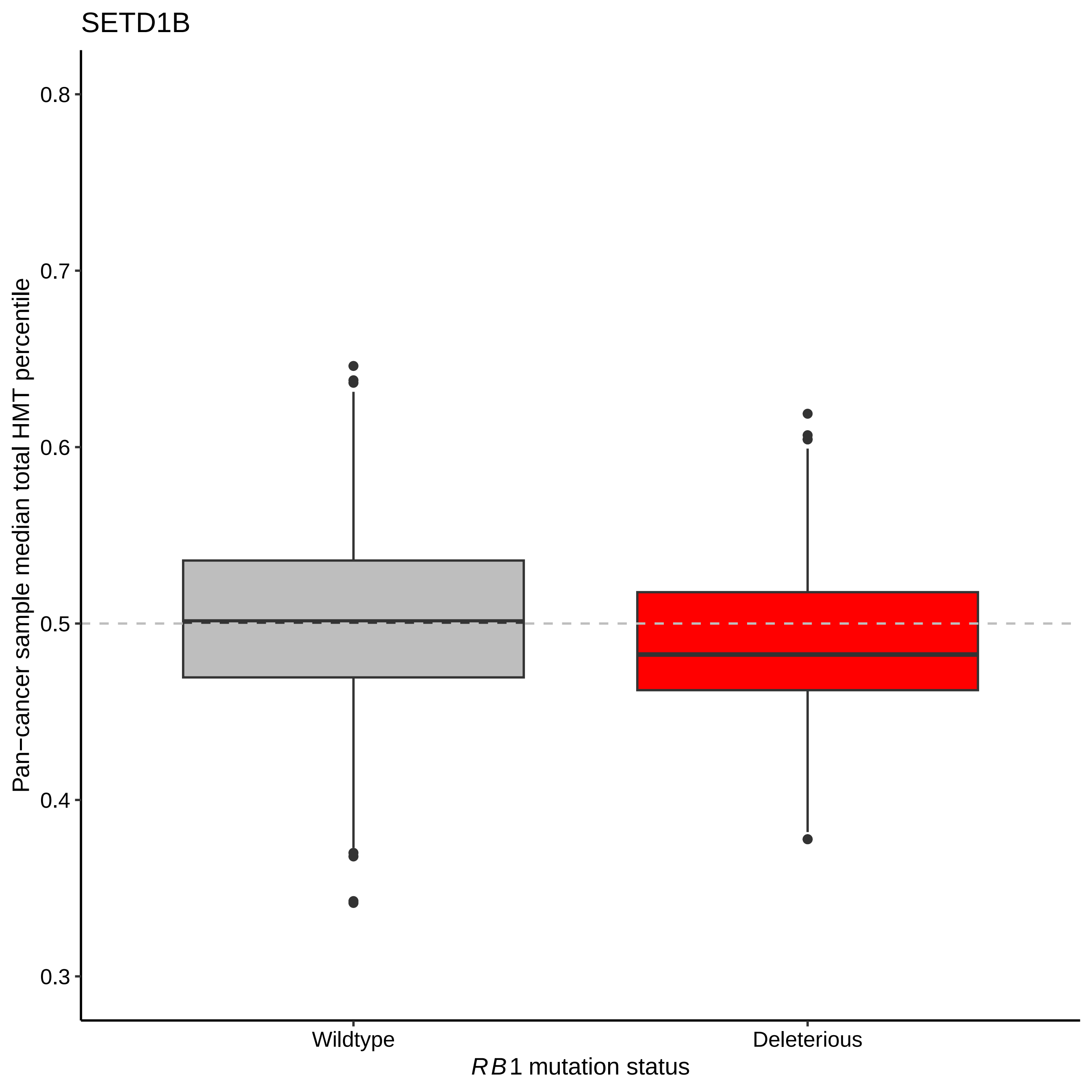
SETD1B
0.8
0.7
0.6
0.5
0.4
0.3
Pan−cancer sample median total HMT percentile
Wildtype
Deleterious
RB1 mutation status

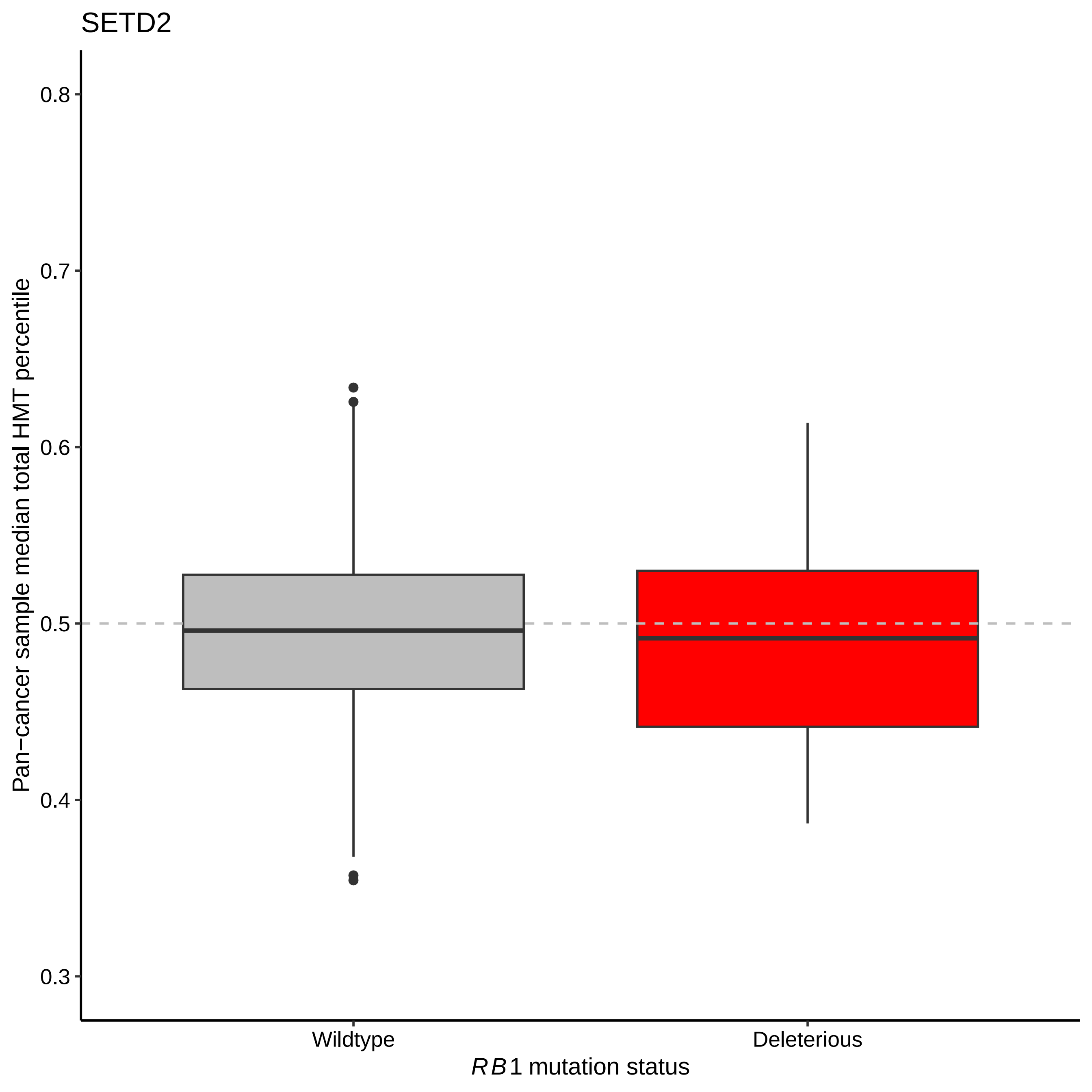
SETD2
Pan−cancer sample median total HMT percentile
0.8
0.7
0.6
0.5
0.4
0.3
Wildtype
Deleterious
$RB1$ mutation status

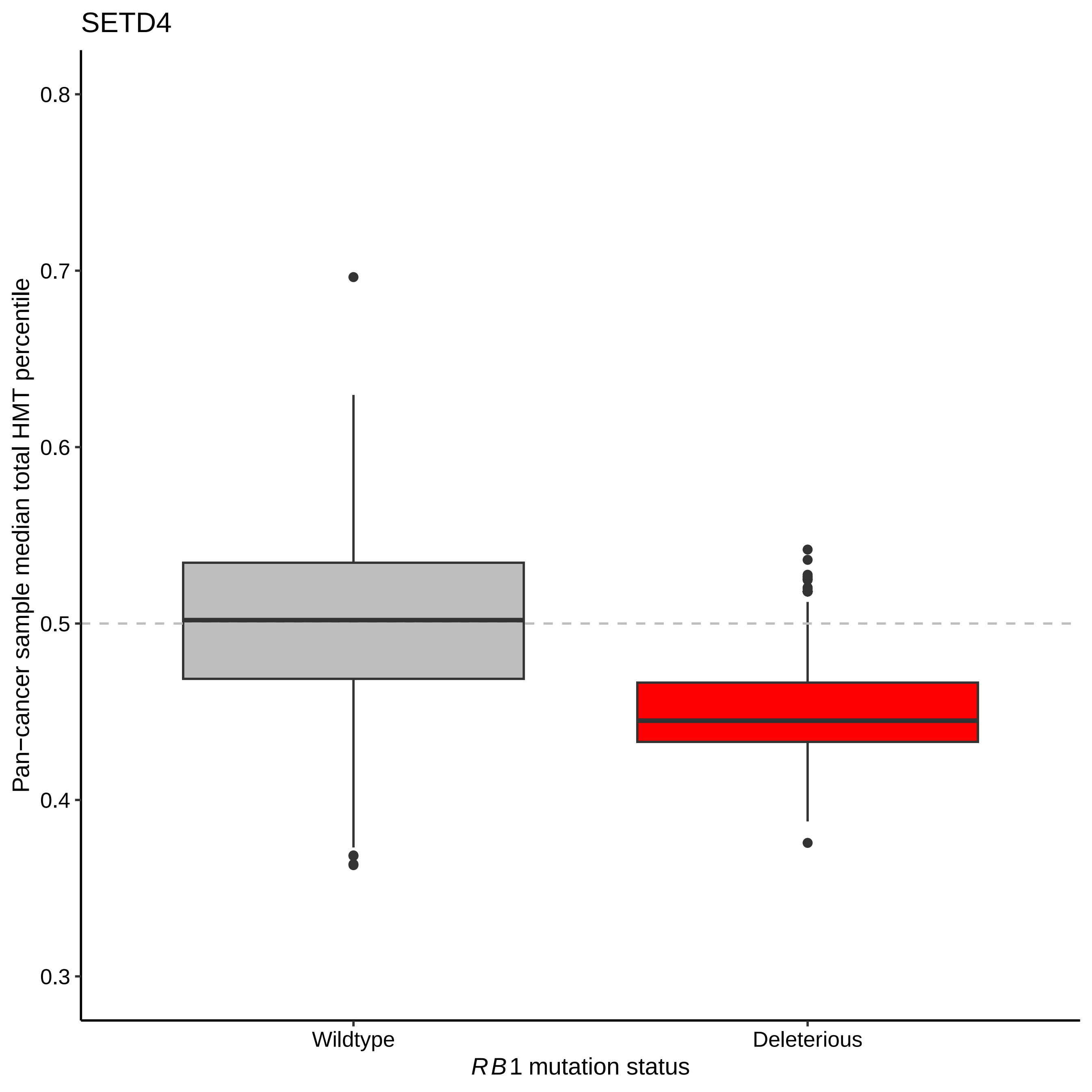
SETD4
Pan−cancer sample median total HMT percentile
0.8
0.7
0.6
0.5
0.4
0.3
Wildtype
Deleterious
RB1 mutation status

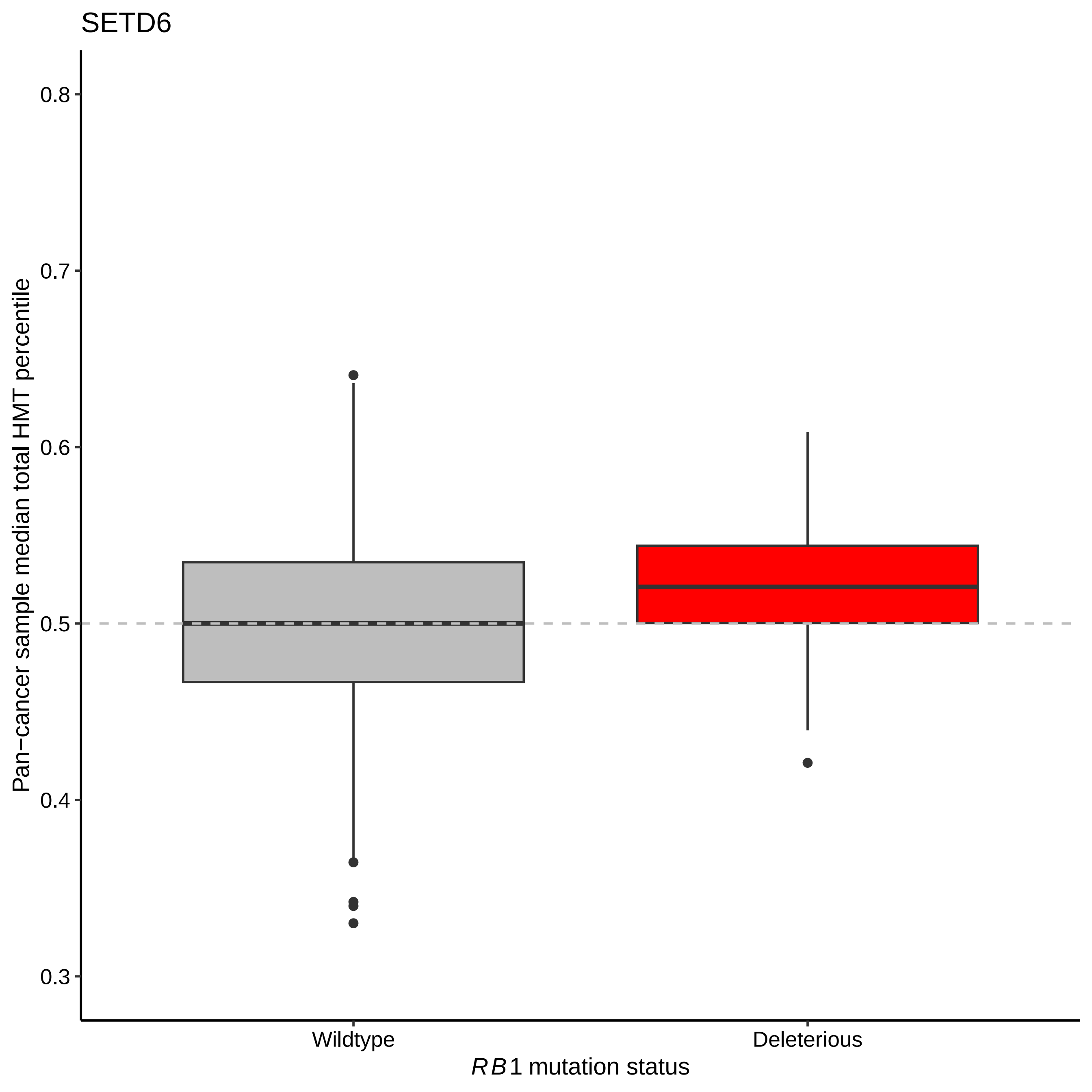
SETD6
Pan−cancer sample median total HMT percentile
0.8
0.7
0.6
0.5
0.4
0.3
Wildtype
Deleterious
RB1 mutation status

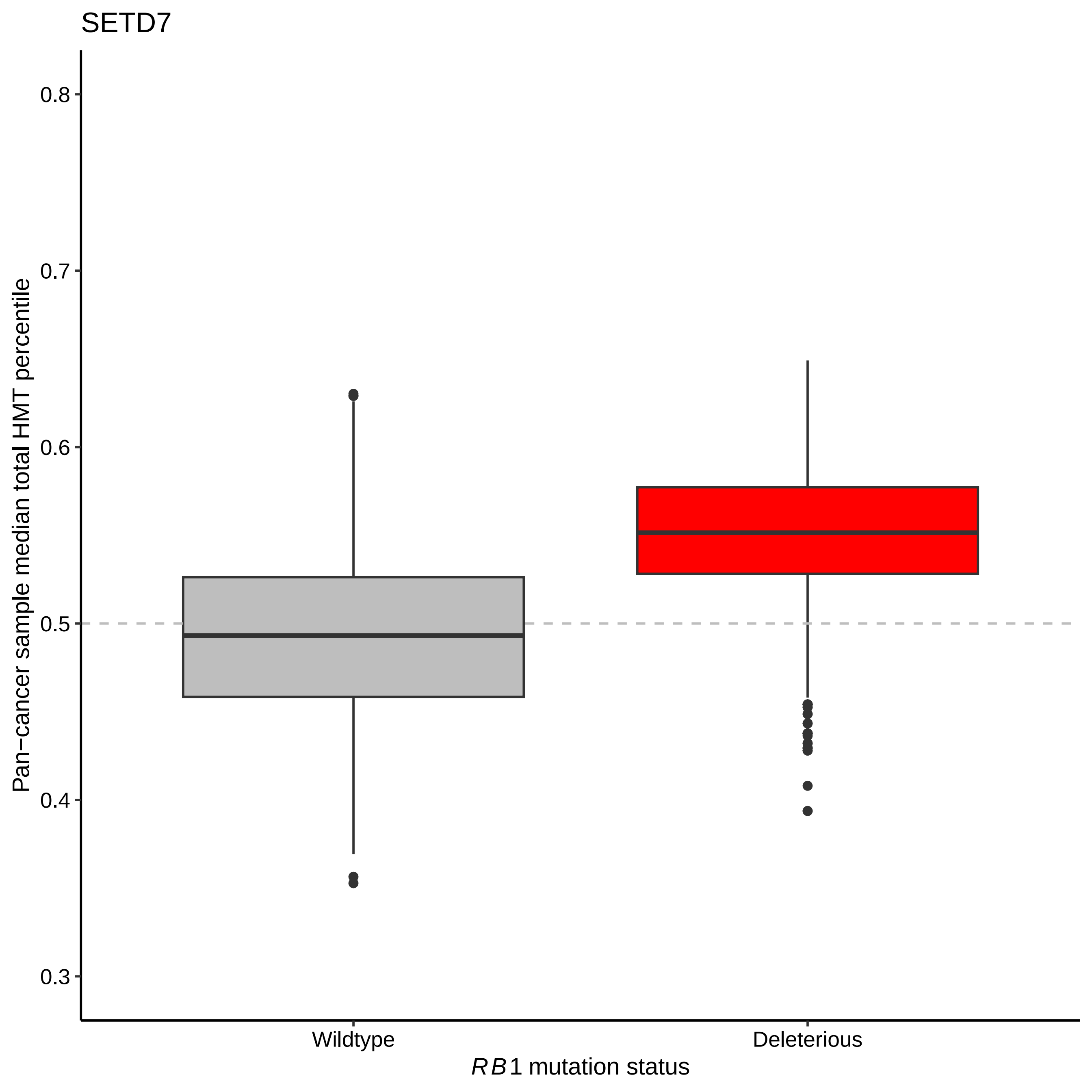
SETD7
0.8
0.7
0.6
0.5
0.4
0.3
Pan−cancer sample median total HMT percentile
Wildtype
Deleterious
RB1 mutation status

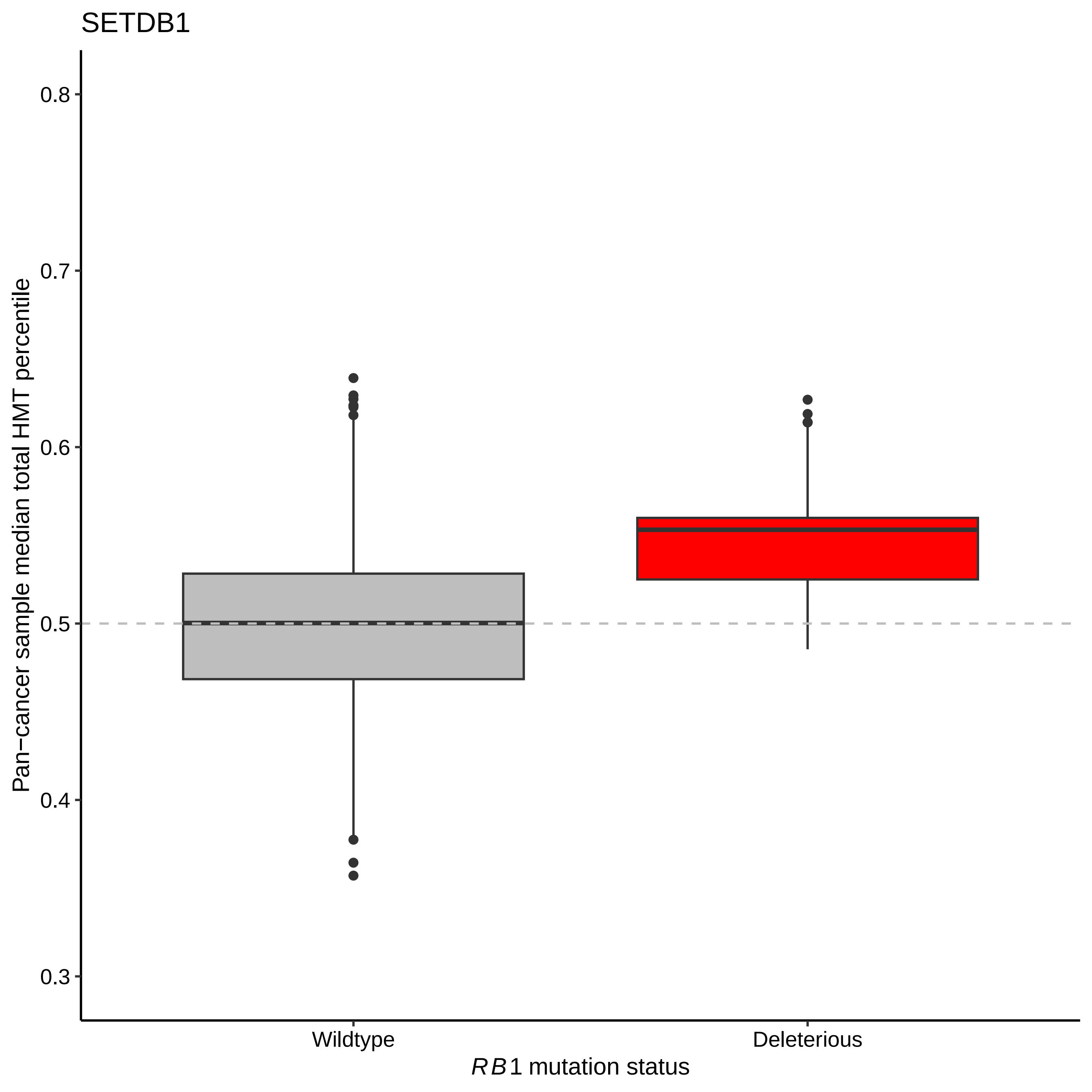
SETDB1
Pan−cancer sample median total HMT percentile
0.8
0.7
0.6
0.5
0.4
0.3
Wildtype
Deleterious
RB1 mutation status

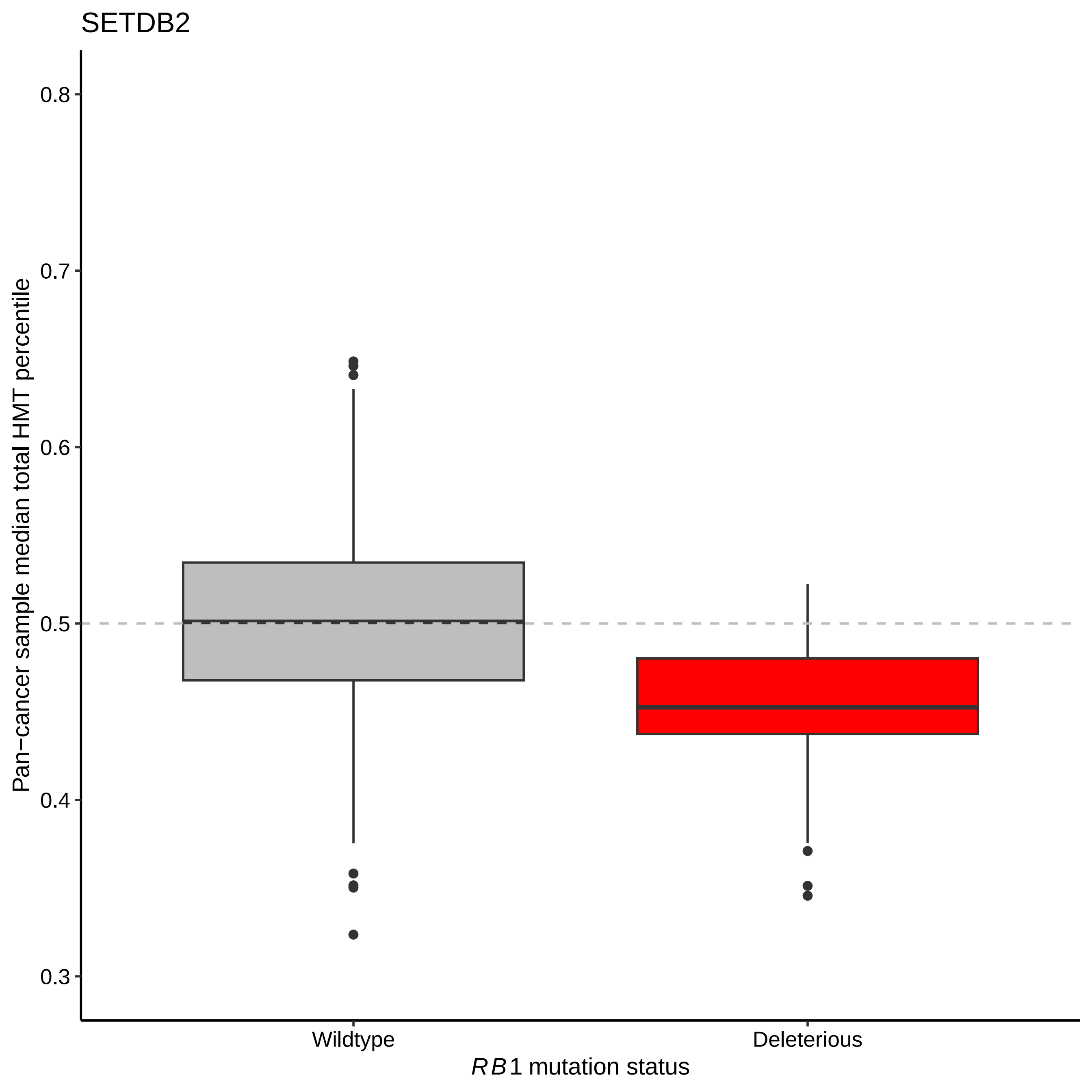
SETDB2
Pan−cancer sample median total HMT percentile
0.8
0.7
0.6
0.5
0.4
0.3
Wildtype
Deleterious
RB1 mutation status

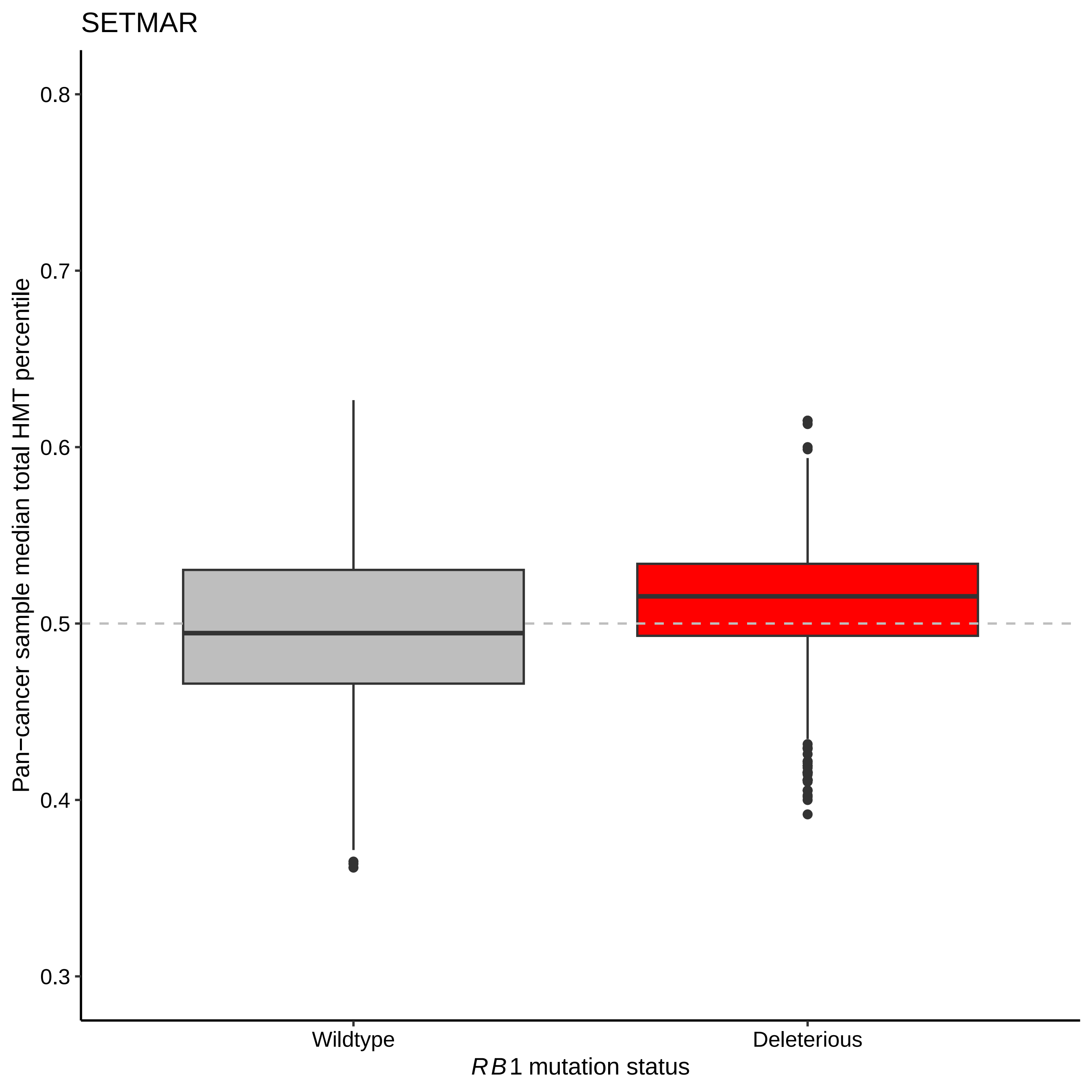
SETMAR
Pan−cancer sample median total HMT percentile
0.8
0.7
0.6
0.5
0.4
0.3
Wildtype
Deleterious
RB1 mutation status

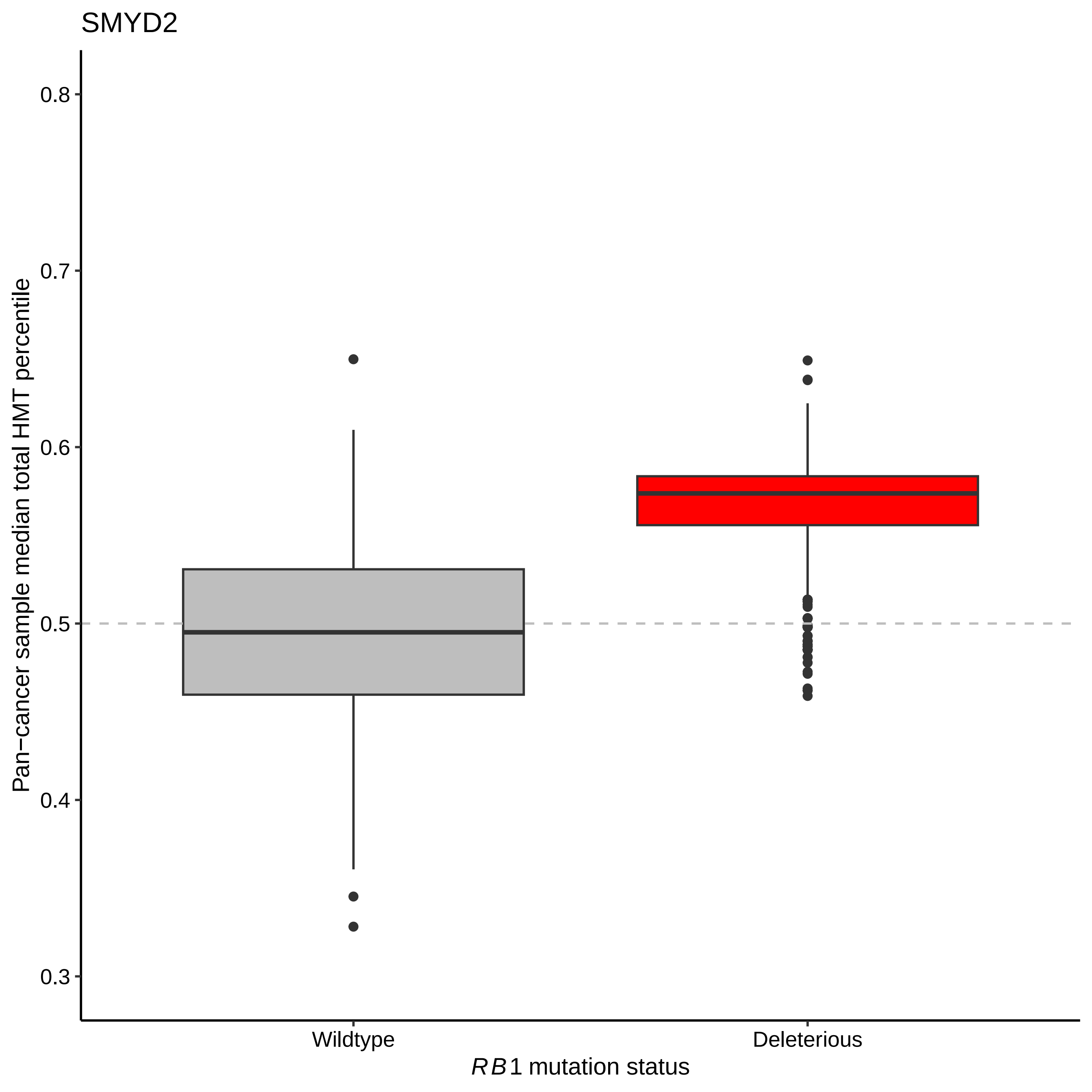
SMYD2
Pan−cancer sample median total HMT percentile
0.8
0.7
0.6
0.5
0.4
0.3
Wildtype
Deleterious
RB1 mutation status

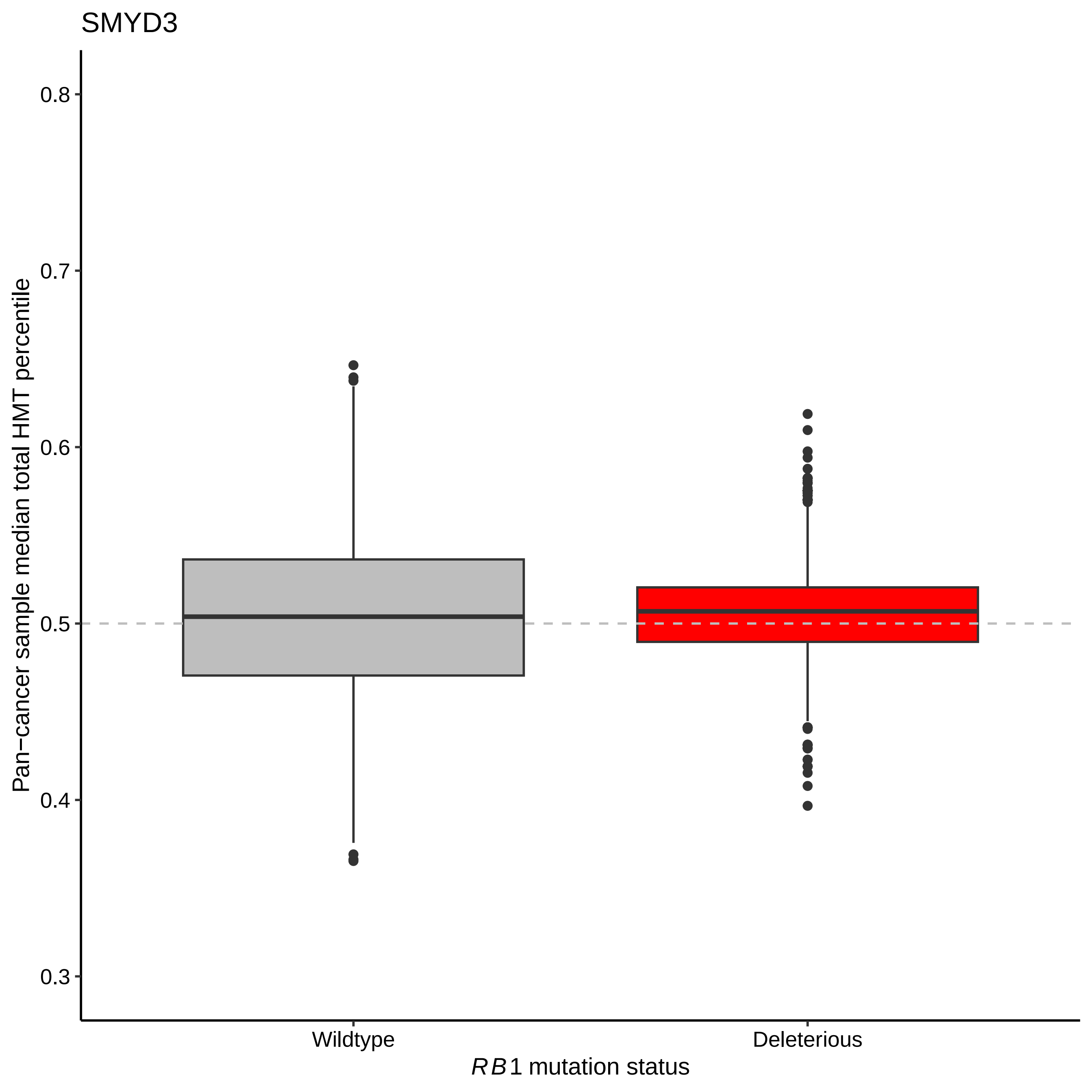
SMYD3
Pan−cancer sample median total HMT percentile
0.8
0.7
0.6
0.5
0.4
0.3
Wildtype
Deleterious
RB1 mutation status

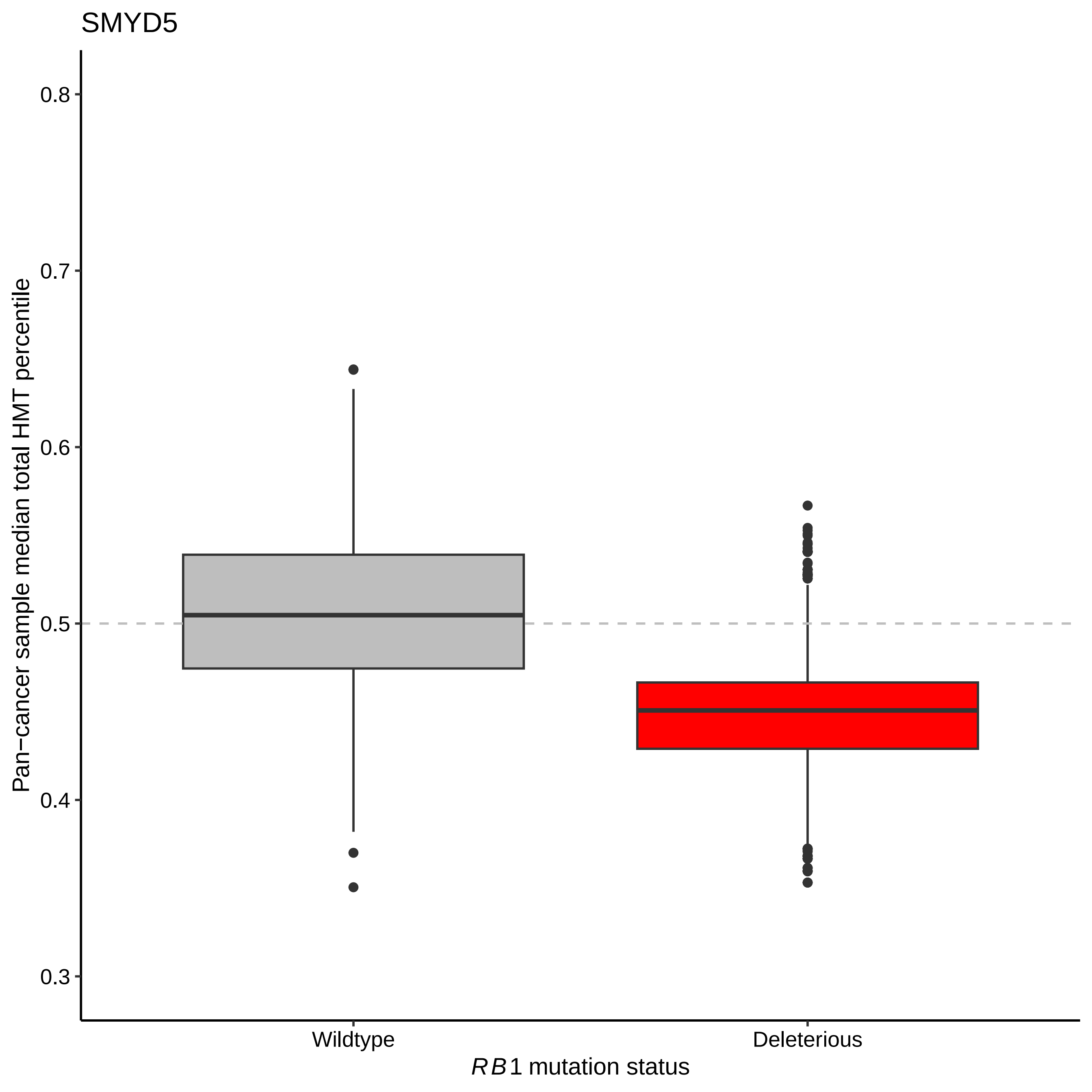

SMYD5
Pan−cancer sample median total HMT percentile
0.8
0.7
0.6
0.5
0.4
0.3
Wildtype
Deleterious
RB1 mutation status

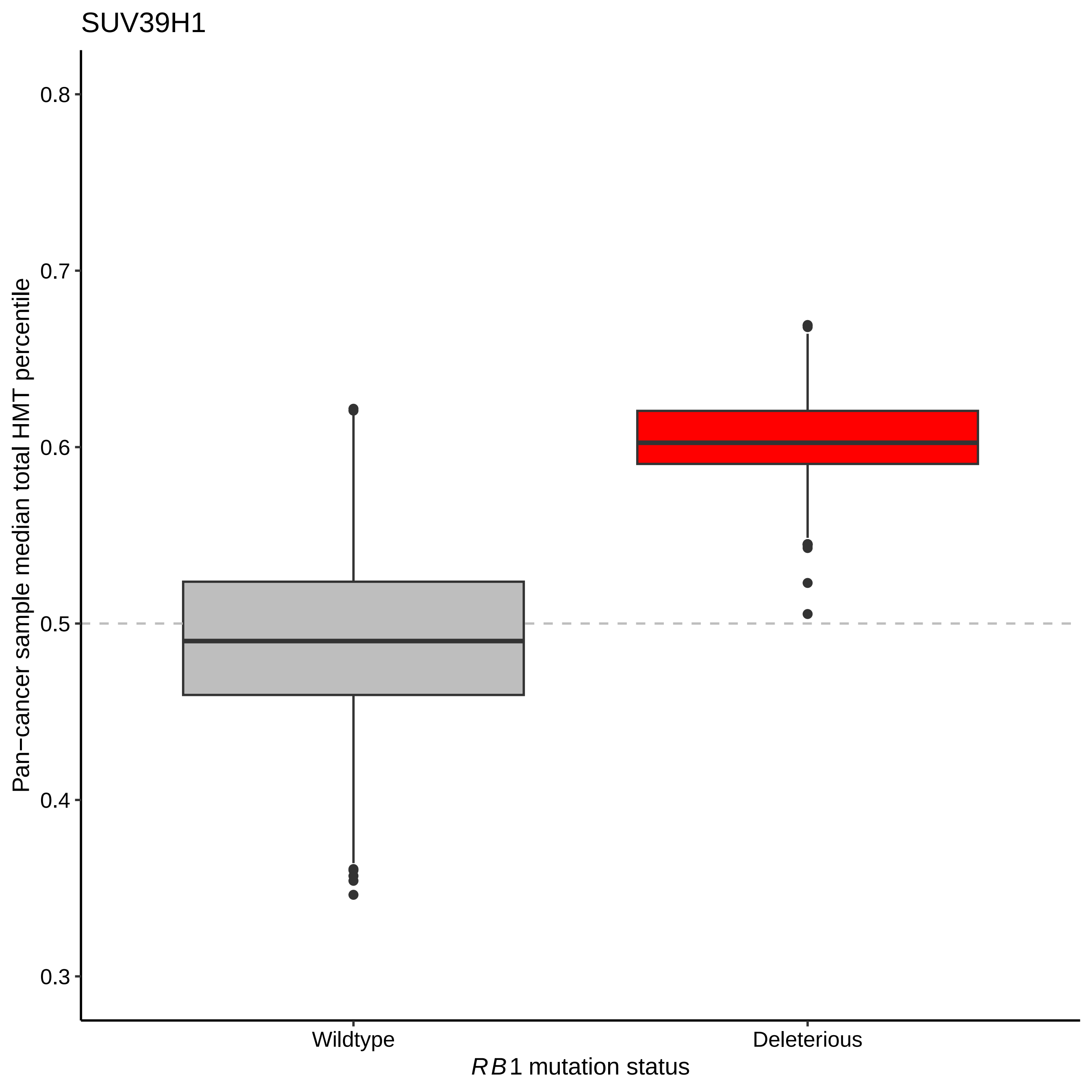

SUV39H1
Pan−cancer sample median total HMT percentile
0.8
0.7
0.6
0.5
0.4
0.3
Wildtype
Deleterious
RB1 mutation status

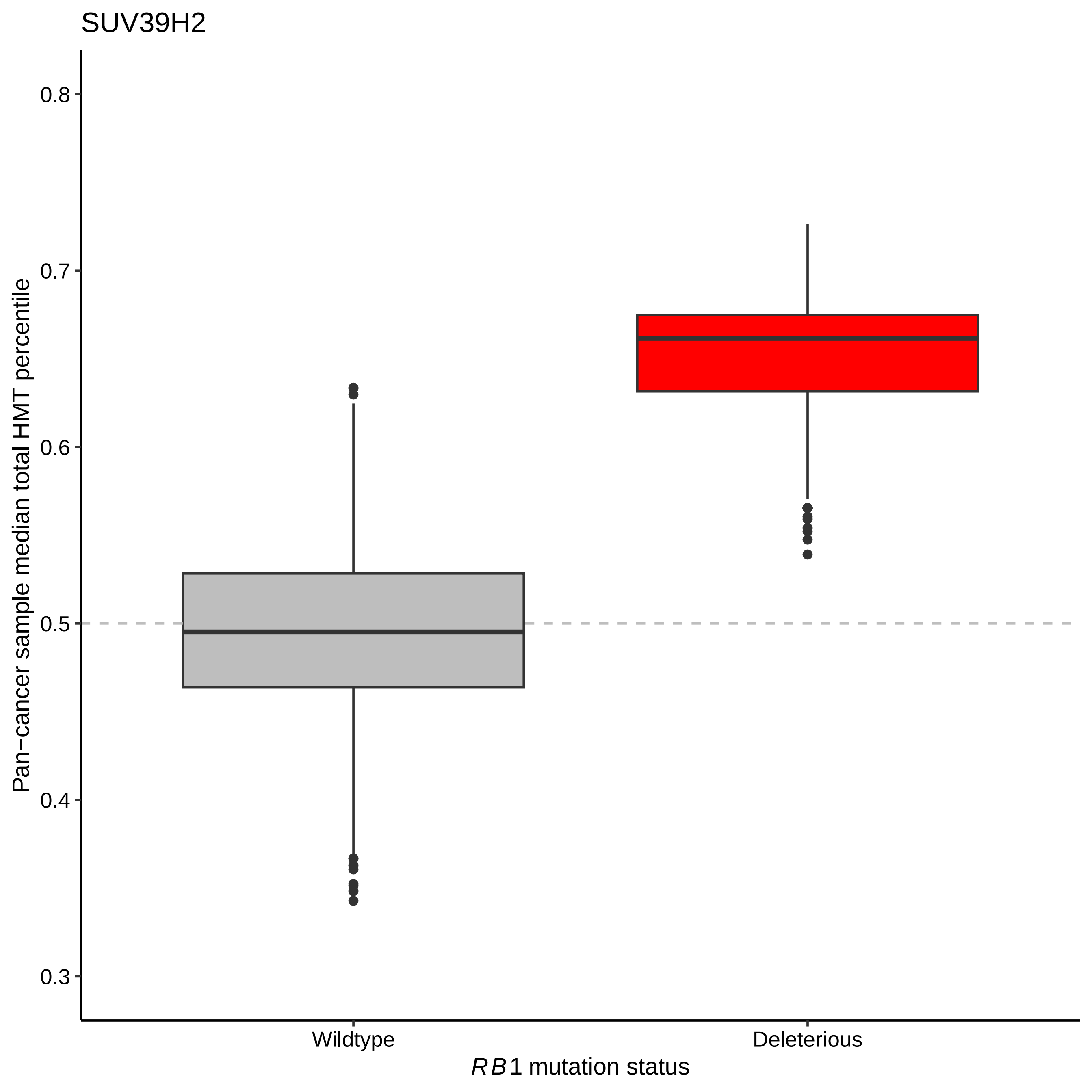
SUV39H2
Pan−cancer sample median total HMT percentile
0.8
0.7
0.6
0.5
0.4
0.3
Wildtype
Deleterious
*RB*1 mutation status
