# Supplementary material for: Histone methyltransferase activity affects metabolism in human cells independently of transcriptional regulation
Source: PLoS Biol. 2023 Oct 26;21(10):e3002354. doi: 10.1371/journal.pbio.3002354 (PMC10602318; doi:10.1371/journal.pbio.3002354)
Supplement: Attachment — Submitted filename: Response_to_reviewers[PSMFP]^4.docx [file pbio.3002354.s030.docx]

**Reviewer 1**

In this manuscript, the authors first performed correlation analyses between the expression level of histone methyltransferases (HMTs) and either metabolite levels or expression of other genes. They found 1) negative correlations between HMTs and the metabolite 1-methylnicotinamide (1MNA), a product of methylation of nicotinamide by the enzyme NNMT, in cancer cell lines, 2) negative correlations between HMTs and NNMT in both cancer cell lines and tumors, and 3) negative correlations between HMTs and PEMT, another enzyme catalyzing a methylation reaction, in normal tissues. In the second part of the manuscript, the authors focused on the positive correlations among HMTs and through bioinformatic analysis identified E2F and Rb as transcriptional regulators of HMTs.

The manuscript shows clear and intriguing associations between HMTs and levels of 1MNA methyl sink enzymes. And the identification of two new transcriptional regulators of HMTs is another major result. My main concern however is that the main claim of the manuscript remains a hypothesis and is beyond the data.

As stated in the title, the main claim is that "Histone methylation has a direct metabolic role in human cells". To make this claim, the author would need to show that it is the activity of HMTs that cause the changes in 1MNA and the expression of NNMT or PEMT. The correlations found do not support any causal relation. The only piece of data for supporting a causal relation (that HMTs are upstream of the methyl pool and other methyl sink pathways) is the analysis of gene expression data of Rb, HMTs, and NNMT (Fig. 5G-H). This is however very indirect evidence as it requires the establishment of Rb as the regulator of HMTs in the first place. Furthermore, the effect size shown in Fig. H is small, reducing the strength of this piece of evidence.

I understand that direct manipulations of the HMT activities would require major experimental effort and may be beyond the scope of the study. But the authors should **tune down their claim** or focus on the E2F and Rb part of their study.

We thank the reviewer for their response to our manuscript. We agree with the reviewer that our analysis identifies a correlation but does not prove a causative link between histone methyltransferase activity and NNMT activity. Additionally, we did not intend to imply that the metabolic changes associated with altered HMT levels demonstrate a primary function for HMTs in metabolic regulation. We have therefore carefully adjusted the wording of our manuscript to clarify that we provide strong evidence for metabolic effects of total HMT activity, independent of transcriptional effects. The title of our revised manuscript reflects this adjustment: ‘**Histone methyltransferase activity affects metabolism in human cells’.**

Nevertheless, we do not agree that the effect size shown in Fig. 4G-H (formerly Fig 5G-H) is small. Inclusion of the HMT expression in *RB1* mutant cancers fully abolishes, and indeed even slightly reverses, the highly significant statistical relationship observed between *RB1* mutation and reduced *NNMT* expression. We accept the reviewer’s point that this is our only piece of evidence which points towards the causal relationship we suggest. We have expanded the Discussion to make this clear.

“We have shown a strong anticorrelation between HMT levels and NNMT activity in cancer. These results fit with earlier findings that changes in *NNMT* expression could modulate histone methylation (Ulanovskaya, Zuhl et al. 2013, Kraus, Yang et al. 2014, Sperber, Mathieu et al. 2015, Eckert, Coscia et al. 2019). This was previously argued to be due to a passive effect of NNMT activity on cellular methylation potential via the SAM/SAH ratio. However, we showed that this relationship corresponds to differences in expression of HMTs and *NNMT.* We argue that this is better explained by the hypothesis that elevated histone methylation activity results in a reduced SAM/SAH ratio. We presented evidence that *NNMT* expression changes downstream of HMT expression in Rb-mutant cancers.

[…]

It is possible that the coordinated change in HMT activity has a function in buffering the SAM/SAH ratio. However, this would predict that HMT expression should be reduced when the SAM/SAH ratio is reduced. We did not observe this; indeed, HMT activity was actually somewhat increased when the SAM/SAH ratio was artificially reduced by perturbing Ahcy function. Thus we favour the hypothesis that changes in the SAM/SAH ratio are an important consequence of alterations in HMT activity rather than their primary function.”

**Reviewer 2**

This manuscript presents evidence that histones function as methyl sinks in both normal and cancer cells. The authors have carefully analyzed numerous publicly available datasets, noting an inverse correlation between the expression of histone methyltransferases (HMTs) and nicotinamide N-methyltransferase (NNMT) in cancer cells, as well as phosphatidylethanolamine N-methyltransferase (PEMT) in normal tissues. Both of these enzymes, previously shown to consume S-adenosylmethionine (SAM) to generate 1-methylnicotinamide (1MNA) and phosphatidylcholine (PC), respectively, also produce S-adenosylhomocysteine (SAH) as a byproduct. Given this anticorrelation, the established influence of NNMT or PEMT on the regulation of the SAM/SAH ratio, and an extensive examination of histone modifications, the authors argue that histone methylation represents an alternative mechanism for adjusting the SAM/SAH ratio. Their further analysis suggests that changes in histone methylation are likely
independent of gene expression. Lastly, they provide interesting evidence indicating that E2F and Rb probably control the coordinated expression of HMTs as a group.

The manuscript is compelling and makes ingenious use of published datasets. The findings are intriguing, their data analysis is thorough and sound, and their conclusions are well substantiated by the provided evidence. The paper is very well-written and was a pleasure to read. I recommend it for publication, subject to minor revisions. I have the following questions and suggestions.

Given the alternative sinks for methyl groups, could the authors provide insight into the factors/conditions that dictate the preferential use of one sink over another? For example, why is NNMT predominantly used in some cancer cells/conditions, while HMTs are employed in others?

We note that in the CCLE dataset high HMT expression is associated with high levels of the characteristic transsulphuration metabolites cystathionine and alpha-hydroxybutyrate. Additionally we note that all of the enzymes involved in transsulphuration and glutathione synthesis are annotated as having nuclear localisation in the Human Protein Atlas (HPA). Although it is not annotated as nuclear in the HPA, the SAH hydrolase AHCY has been reported in several studies to be nuclear and chromatin-associated, which is hypothesised to hydrolyse SAH and maintain a local environment which is conducive to methylation. However the thermodynamics of the reaction strongly favour the formation of SAH, rather than its hydrolysis, unless the SAH breakdown products homocysteine and adenosine are rapidly metabolised. This would argue for nuclear activity of either the methionine cycle or the transsulphuration pathway, driven by histone methylation.

Meanwhile, NNMT has been reported as physically associated with methionine cycle enzymes in the liver (Hong, Zhai et al. 2018). While this may not generalise to all tissues and cancers, it implies that SAH produced by NNMT may be preferentially funnelled back into the methionine cycle and therefore be unavailable for transsulphuration.

This suggests that of the two pathways, histone methylation is more associated with high levels of transsulphuration. While this may not be the ‘purpose’ of a regulated increase in HMT expression, it is possible that cancer cells in particular may undergo selection for HMT expression due to increased transsulphuration activity which produces the critical metabolites cysteine and glutathione.

We have added a paragraph in the Discussion and a Supplementary Table indicating the nuclear localisation of the relevant enzymes in the annotations from the Human Protein Atlas:

“Several studies have placed the SAH hydrolase AHCY in the nucleus associated with chromatin, arguing that it maintains a local environment conducive to histone methylation (Radomski, Kaufmann et al. 1999, Grbeša, Kalo et al. 2017, Ponnaluri, Estève et al. 2018, Aranda, Alcaine-Colet et al. 2019). However AHCY-catalysed SAH hydrolysis is reversible and is thermodynamically favoured only when the breakdown products are rapidly metabolised (Finkelstein 1990). This hypothesis would therefore predict nuclear activity of either the methionine cycle or the transsulphuration pathway. Given that all of the enzymes required for transsulphuration and glutathione synthesis are annotated as having nuclear localisation (**Table S9**) in the Human Protein Atlas (Thul and Lindskog 2018), the existence of a nuclear transsulphuration pathway fuelled by histone methylation, potentially supplying cysteine for nuclear glutathione synthesis, is an interesting possibility for future exploration.”

The transsulphuration pathway facilitates the conversion of SAM to SAH, homocysteine, and subsequently cysteine, which can be synthesized into downstream products such as glutathione (GSH). Do the authors notice any correlations between HMTs, NNMT, or PEMT with enzymes involved in cysteine biosynthesis? Could a limitation in cysteine supply influence the preference for one pathway over another, perhaps due to flux or other physiological parameters optimizing SAM to SAH conversion?

As noted in the Discussion, we see correlations in the CCLE dataset between high HMT-expressing cell lines and high levels of the characteristic transsulphuration metabolites cystathionine and alpha-hydroxybutyrate. However, we do not see strong correlations between the expression of genes encoding for cysteine biosynthesis enzymes (*CBS* and *CTH*) and HMTs, *PEMT* or *NNMT*. In no case are these genes among the top 5% of correlated genes (whether positively or negatively) to HMTs, *NNMT* or *PEMT*.

Lower levels of histone methylation and acetylation have been shown to predict poor outcome in multiple cancer types. Do the authors observe concordant changes in histone acetylation and methylation, especially in the analyses of the cancer tissues? And do these changes correlate with outcome? For instance, do tumors with high NNMT and low histone methylation (or low HMT expression) fare worse than those with low NNMT and high histone methylation? If the authors have access to such datasets, integrating them could bolster the clinical relevance of their findings to cancer prognosis and potentially offer insight into how cells select among various methyl sinks.

We thank the reviewer for their suggestions. Whilst investigation of histone acetylation would be interesting, it is an entirely separate study which is beyond the scope of the current manuscript.

However, we have investigated how histone methylation and NNMT levels might impact survival. We performed survival analysis for TCGA cancer types to compare high and low expression of HMTs or NNMT. There are a few cancer types where *NNMT* or HMT expression have significant effects on survival (high *NNMT* leads to worse prognosis in STAD and LGG, whereas high HMT leads to worse prognosis in ACC but better prognosis in UVM). Combining both HMT and NNMT expression for survival analysis mostly only shows significant effects in the aforementioned cancers, where the effect of only *NNMT* or HMTs is already considerable and which is therefore difficult to disentangle. Since these analyses only show significant effects in a few cancer types, do not have a consistent direction of effect and tell us little about the combination of *NNMT* and HMT expression, we do not think that these would add much to the manuscript.

However we have included another analysis which does suggest that the effect we describe may have clinical relevance in cancer. We note that whilst in most cancer types the strong relationship to *NNMT* emerges upon pooling HMTs together, in skin melanoma a single HMT – *SETDB1* - has a very strong relationship to *NNMT*. In fact, they have a ‘perfect’ relative reciprocal correlation score of 2; *SETDB1* is the most strongly anticorrelated gene in the whole genome to *NNMT* in melanoma and vice versa. This is interesting because *SETDB1* is an established driver of melanoma. This relationship suggests that its oncogenic role could be partly metabolic in nature.

We have introduced this into the text and added a new figure, Fig S4, to illustrate this relationship.

**Fig S4. In melanoma *NNMT* expression is strongly anticorrelated with the histone methyltransferase-encoding *gene SETDB1,* a known driver of melanoma.**

A) Scatterplot showing *NNMT* expression vs *SETDB1* expression in 102 melanoma primary tumour samples from the TCGA. Values plotted are residuals after controlling for age, race, gender and tumour stage.

B) Scatterplot showing *NNMT* expression vs *SETDB1* expression in 367 melanoma metastases from the TCGA. Values plotted are residuals after controlling for age, race, gender and tumour stage.

C) Boxplot showing the expression of individual HMT-encoding genes in melanoma primary tumours. *SETDB1* (red box) is not an exceptionally highly-expressed HMT in melanoma.

D) Boxplot showing *SETDB1* expression in primary tumours of 33 distinct cancer types from the TCGA. Melanoma (TCGA code SKCM), highlighted with a red box, does not have exceptionally high *SETDB1* expression .

“Total HMT expression was more strongly anticorrelated to *NNMT* than any individual HMT in 16/33 cancer types (**Supp. File 2).** However, one notable exception was melanoma (SKCM), which despite being one of the cancer types with the strongest *NNMT-*total HMT relationships exhibited a far stronger anticorrelation of *NNMT* to the euchromatic H3K9me3 writer *SETDB1* (**Fig S4, Supp. File 2**). *SETDB1* is the most strongly anticorrelated gene in the genome to *NNMT* and vice versa. As *SETDB1* is a recurrently amplified, established driver of melanoma (Ceol, Houvras et al. 2011, MacGregor, Montgomery et al. 2011, Orouji, Federico et al. 2019, Griffin, Wu et al. 2021), this raises the possibility that increased *SETDB1* activity might have important metabolic consequences for melanoma.”

Lastly, the authors use the term "sink" to suggest that the deposition of methyl groups on histones might be terminal as far as the methyl groups are concerned. However, are there known mechanisms to recover methyl groups from histones,1MNA or PC molecules? If so, would it not be more appropriate to use the term "storage" instead?

Acetyl groups added to histones can be rapidly recovered as usable acetate, which is why ‘sink’ would in that case be an inappropriate label and ‘reservoir’ or ‘storage’ would apply well. However methyl groups deposited into methyl sinks are not easily recovered, which is why the term sink is appropriate in this case. Methyl groups consumed via NNMT activity are excreted with 1MNA and lost to cellular metabolism. Demethylation of histones produces formaldehyde; formaldehyde may be recycled to regenerate methionine but the route is unclear and likely to be circuitous. Meanwhile methyl groups used by PEMT to convert phosphoethanolamine to phosphatidylcholine could potentially re-enter the methionine cycle, with the choline head group used to regenerate methionine from homocysteine, but this reaction likely only occurs in the liver (Finkelstein 1990).

We have added the following text to the introduction to make this distinction clearer:

“Histones have the potential to act as a methyl sink, as histone methylation consumes methyl groups from SAM but demethylation releases formaldehyde, which cannot be easily recycled to release methyl groups (Mosammaparast and Shi 2010).”

**Reviewer 3**

In this study titled 'Histone methylation has a direct metabolic role in human cells', the authors propose that methyl transferase enzymes control the role of different methyl sinks, and thereby regulate the methylation status in cells. The authors find anti-correlations between histone methyl transferases and other methyl sinks such as NNMT and PEMT enzymes in cancers and healthy tissues. They also uncover a possible role for the Retinoblastoma (Rb) protein in regulating the expression of HMT and NNMT. The authors thereby propose that this mode of regulation of methyl sinks can modulate SAM homeostasis, and thereby the cellular metabolic state.

The strength of this manuscript are the correlations between HMT and the other methyl transferases-NNMT and PEMT in cancers and healthy tissues and uncover the possible mode of regulation of HMT expression. However, the role of these in regulating the cellular SAM pools or metabolic state is not clear, and the claim that this is involved "directly in core, conserved pathways of cellular metabolism" is an overstatement. There are multiple really interesting correlations from this study, and the central hypothesis is very nice, but the authors try to connect these with cellular metabolism without sufficient evidence and this overshadows their actual findings. The following are the concerns with this study.

Major concerns:
1) The title of the study "Histone methylation has a direct metabolic role in human cells" is very broad, and the study does not justify this title. The authors have done extensive analysis to understand how the expression of different methyl transferases correlate. Even though the results can be extended to understand how methyl sinks in the cells are regulated, in the absence of specific analyses to address this question, the title is an exaggeration. Similarly, the abstract and discussion also makes strong claims about how this impacts metabolism, with insufficient data.

We thank the reviewer for their critique of our study. We have endeavoured to tone down the claims that we make so that they are commensurate with the evidence provided, in addition to strengthening and refining the link between SAM/SAH and transcriptional regulation of HMTs/NNMT, as described below. We have also adjusted the title of the manuscript, which is now: ‘**Histone methyltransferase activity affects metabolism in human cells**’. We apologise if the previous title was read to mean that the metabolic changes associated with altered HMT levels demonstrate a primary function for HMTs in metabolic regulation, which we did not intend to imply.

2) It is not clear whether and how HMT expression varies between healthy and normal cells. In fig 1A, HMT expression is negatively corelated with lactate, which is often increased in cancers. Is HMT levels generally downregulated in cancers? If a comparison is made between healthy and cancer cells, how does the expression of different methyl transferases change and what will be the correlation among them?

We thank the reviewer for suggesting that we investigate this. We find that the expression of HMTs is sometimes altered in cancer, but not consistently. Of 12 cancer types with at least 30 pairs of normal-cancer matched samples, we find a significant upregulation in 4 cancer types, downregulation in 2 cancer types and no change in 6 cancer types.

*NNMT* is reported to be often overexpressed in cancers. However in the TCGA, we find that while *NNMT* is upregulated in 3 cancer types, downregulated in 4 and not significantly altered in 5.

However, interestingly cancers which tend to strongly upregulate *NNMT* tend to downregulate HMT expression and vice versa, consistent with the strong anticorrelation that we observed between *NNMT* and HMT expression.

We have now included these results in the manuscript as Fig S6, together with the existing cancer vs normal plot of *NNMT*-HMT correlations which was previously Fig S2K, and amended the text as follows:

“We wondered whether the difference between cancer and healthy tissue could be due to differences in the expression levels of *NNMT* and HMTs. However, while *NNMT* has frequently been reported as overexpressed in cancers (Roeßler, Rollinger et al. 2005, Lim, Cho et al. 2006, Wu, Siadaty et al. 2008, Kim, Hong et al. 2009, Tomida, Mikami et al. 2009, Tang, Yang et al. 2011, Zhou, Gui et al. 2014, Harmankaya, Akar et al. 2021), we did not find strong support for this across the TCGA. *NNMT* was significantly upregulated in tumour samples compared to matched normal samples in 3/12 cancer types, with 4/12 showing significant downregulation (**Fig S6B**). Similarly, total HMT expression was not consistently altered in cancer, being upregulated in 4/12 cancer types and downregulated in 2/12 (**Fig S6C**). Interestingly, however, we observed that these changes were inversely correlated: cancers which upregulate HMTs have reduced *NNMT* expression and vice versa (**Fig S6D**).”

**Fig S6. *NNMT* and HMT expression and relationship in cancer vs normal samples.**

A) Scatterplot showing HMT-*NNMT* relative reciprocal relationship scores in TCGA primary tumour samples and matched normal samples. Note the axes are inverted as a lower score indicates a stronger relationship.

B) Boxplots of *NNMT* expression in matched normal and tumour samples for 12 TCGA cancer types. Tests are paired t-tests with FDR correction for multiple testing across cancer types.

C) Boxplots of total HMT expression in matched normal and tumour samples for 12 TCGA cancer types. Tests are paired t-tests with FDR correction for multiple testing across cancer types.

D) Paired t-test statistic for HMT expression for matched cancer vs normal samples in 12 TCGA cancer types plotted against t statistics for *NNMT* expression.

*, FDR < 0.1. **, FDR < 0.01. ***, FDR < 0.001. ****, FDR < 0.00

3) One of the bigger concerns regarding this study is that it does not address how the changes in the expression of HMT, PEMT and NNMT actually regulate the SAM or SAH levels. Do SAM, SAH (or methionine) levels change in Rb mutant tumors where HMT expression is upregulated? Or does the decrease in NNMT, which compensates for the HMT increase, stabilize the SAM and SAH pools? In the absence of evidence from such experiments, the role of HMT and other methyl transferases in regulating methylation pool is severely overstated.

Note that SAM/SAH ratios cannot be calculated from the metabolomic datasets that we used so we could not look at this directly in the data. However, prompted by the reviewer’s suggestion we did attempt to use the dataset to investigate other potential effects of RB on metabolism; however RB mutant cell lines in the CCLE were limited to 32 mutants and 18 wildtype in small cell lung cancer, which is the only cancer type with a reasonable number of RB1 mutants. Within this set we did not observe any significant differences in metabolite levels but this may be due to lack of power arising from small numbers of cell lines. We accept the reviewer’s point and have toned down our claims in the text.

4) Correspondingly, is there any correlative data to suggest that changes in HMT, NNMT or PEMT occur when there are defects methionine or SAM metabolism? If there is a defect or decrease in SAM synthesis (and therefore the methylation source), would the expression of these methyl transferase enzymes change correspondingly to compensate for these? This would strengthen the correlative argument that the changes in these enzymes have a direct role in regulation methylation pool/ SAM metabolism. In the absence of data related to the same, the claim that "our results indicate histone methylation participates directly in core, conserved pathways of cellular metabolism" is not supported. I'd encourage the authors to think hard about their statements, and what makes very strong arguments, vs which ones are weaker.\

We thank the reviewer for the suggestion, which has turned out to be very useful for our analyses and to generate important new information. We identified two data sets involving loss of function of the SAH hydrolase Ahcy, via either CRISPR deletion or by pharmacological inhibition with the drug DZNep, in mice embryonic fibroblast and rat liver, two cell/tissue types which have established *NNMT* expression/function. The principal effect of Ahcy loss of function is to disrupt the methionine cycle, leading to accumulation of both SAM and SAH but a strong reduction in the SAM/SAH ratio (aka ‘methylation potential’), which we argue is the key parameter which is buffered by methyl sinks. This allows us to investigate how *Nnmt* or HMT expression changes when the SAM/SAH ratio is altered.

We found that in mouse Ahcy loss of function, where the SAM/SAH ratio is reduced, the expression of *Nnmt* is strongly reduced, which is consistent with our hypothesis that NNMT is repressed when the SAM/SAH ratio is reduced. We also observed that HMT expression is modestly upregulated.

These data also proved informative in suggesting a possible molecular link between the SAM/SAH ratio and NNMT expression. Upon Ahcy inhibition the most significantly affected transcription factor was Glyr1. Moreover, in a pan-cancer analysis of the TCGA primary tumour data, GLYR1 was the transcriptional regulator which positively correlated most strongly to total HMT expression and likewise negatively correlated most strongly with NNMT, out of 351 transcriptional regulators assayed. Further, of all the transcriptional regulators it is the one whose activity most weakens the relationship between HMTs and *NNMT*. Its activity is upregulated in Rb-mutant tumours and controlling for its activity abolishes the effect of *RB1* mutation on *NNMT* expression. This suggests that Glyr1 mediates the link between HMT and NNMT activity and may be sensing the SAM/SAH ratio. This suggests a connection between the SAM/SAH ratio and HMT/*NNMT* expression in the TCGA.

These results have been added to the manuscript as Fig S12 and Fig S13, reproduced below, together with some new text:

**“Artificially reducing SAM/SAH ratio leads to *Nnmt* downregulation in mammalian cells**

We wanted to establish whether the transcription of *NNMT* responded to alterations in the SAM/SAH ratios. We identified two RNA-seq datasets where the SAH hydrolase *Ahcy* was either knocked out or inhibited pharmacologically in mouse embryonic fibroblasts (MEFs; (Greco, Cervantes et al. 2020)) and rat hepatic stellate cells (Jiang, Xiang et al. 2021). Ahcy loss-of-function causes both SAM and SAH to accumulate and strongly reduces the SAM/SAH ratio (Belužić, Grbeša et al. 2018, Greco, Cervantes et al. 2020).

We found that reducing Ahcy activity either through *Ahcy* deletion or pharmacological inhibition strongly suppressed *Nnmt* expression in both mouse and rat cells (**Fig S12A**). We also examined how HMT expression responded to artificial reduction of the SAM/SAH ratio. In contrast to the downregulation of *Nnmt*, we observed a moderate upregulation of HMT expression in response to loss of Ahcy activity in mouse cells, and no consistent change in rat cells (**Fig S12B**).

The high number of replicates in the mouse data gave us sufficient power to search for transcriptional regulators that might control the response of *Nnmt* expression to artificial perturbation of the SAM/SAH ratio. Glyr1 was the transcriptional regulator that was most significantly activated by either *Ahcy* deletion or Ahcy inhibition in the mouse dataset (**Fig S13A**, **Table S8**), implying that its activity depends on the SAM/SAH ratio. Prompted by this we examined the TCGA human primary tumour dataset to test whether GLYR1 might be involved in regulating *NNMT* in human cells. Of 351 transcriptional regulators GLYR1 activity had both the strongest negative correlation with *NNMT* expression and the strongest positive correlation with total HMT expression (**Table S6**). Taking estimated GLYR1 activity into account weakened the statistical relationship between *NNMT* and HMTs in the TCGA more than any other transcriptional regulator (**Fig S13B**). GLYR1 activity was elevated in *RB1* mutant cancers (**Fig S13C-D**) and taking GLYR1 activity into account abolished the negative statistical relationship between *RB1* mutation and *NNMT* expression (**Fig S13E**). These results imply a role for GLYR1 in suppressing *NNMT* transcription in response to a low SAM/SAH ratio.”

**Figure S12. Transcription of *Nnmt* and HMTs is altered in response to perturbation of the SAM/SAH ratio.**

Loss of function of the SAH hydrolase Ahcy either by CRISPR deletion or by pharmacological inhibition with DZNep in mouse embryonic fibroblasts (EFs) or rat hepatic stellate cells (HSCs) leads to strong downregulation of *Nnmt* in both mouse and rat cells (**A**) and moderate upregulation of HMTs in mouse cells (**B**). Abbreviations: KO, knockout.

**Fig S13. The transcriptional regulator GLYR1 is a candidate mediator of *NNMT* regulation in response to the SAM/SAH ratio.**

A) Boxplots of *Glyr1* expression and inferred Glyr1 activity for Ahcy deletion and pharmacological inhibition with DZNep in mouse embryonic fibroblasts.

B) Violin plot of partial correlations of *NNMT* expression with total HMT expression when taking into account the inferred activities of 351 transcriptional regulators in a pan-cancer analysis of TCGA tumours. GLYR1 activity uniquely weakens the correlation of *NNMT* and HMT expression. Dashed line indicates the *NNMT*-HMT full correlation.

C) Boxplots show median *GLYR1* expression percentile drawn from 1000 iterations of pan-cancer sampling of tumours with wildtype *RB1* or potentially deleterious *RB1* mutations. p-value derived from t test.

D) Boxplots show median inferred GLYR1 activity percentile drawn from 1000 iterations of pan-cancer sampling of tumours with wildtype *RB1* or potentially deleterious *RB1* mutations. p-value derived from t test.

E) Linear model t-values explaining GLYR1 activity and *NNMT* expression for *RB1* mutation status as the sole explanatory variable or jointly considered with *NNMT* expression/GLYR1 activity respectively. p-value derived from t test.

5) How is the expression of PEMT correlated with other methylation/methyltransferase enzymes, especially NNMT in cancer cells vs healthy tissues? There is enough evidence that phosphatidylcholine metabolism is differentially regulated in cancers. Will changes in PEMT expression, and its correlation with other methyl transferase expression tell us more about how methylation states are controlled in cancers?

We have investigated the differences in *PEMT* expression in normal vs cancer cells, in the figure below. There is no systematic tendency for *PEMT* expression to be either increased or decreased in cancer.

We also investigated whether *PEMT* and *NNMT* show a stronger correlation to each other in cancers. We see that there is no remarkable relationship between them in either normal or cancer cells, and no consistent change in that relationship in cancers:

**PEMT-NNMT correlations in matched normal and cancer samples of TCGA**

|  | **Normal samples** | **Matched cancer samples** |
| --- | --- | --- |
| **TCGA-BRCA** | 0.5664044 | 0.11494714 |
| **TCGA-COAD** | 0.1358885 | 0.18118467 |
| **TCGA-HNSC** | -0.2724252 | -0.15795832 |
| **TCGA-KIRC** | 0.1011641 | 0.18602482 |
| **TCGA-KIRP** | -0.2620968 | 0.19919355 |
| **TCGA-LIHC** | 0.2352461 | 0.17704682 |
| **TCGA-LUAD** | 0.1505056 | 0.20624838 |
| **TCGA-LUSC** | 0.3002041 | 0.04479592 |
| **TCGA-PRAD** | -0.2275250 | 0.12806284 |
| **TCGA-STAD** | -0.2094017 | 0.14468864 |
| **TCGA-THCA** | -0.2029284 | -0.31366698 |
| **TCGA-UCEC** | -0.7176736 | 0.26933936 |

Overall these data are not in support of a clear role for PEMT in regulating methylation states in cancers.

6) Is there any information regarding the localization of methyl transferases, their correlation between their expression? For example, is the correlation between methyl transferases present in the same cellular compartment better compared to those in other compartments? Is this involved in regulating SAM pools in specific compartments such as nucleus, cytosol and mitochondria? If there is some data in this space, it might allow the authors to make a much stronger correlative claim, related to metabolic state and the function/activity of methyltransferases.

We thank the reviewer for the interesting suggestion. As a small molecule SAM is freely diffusible through the nuclear pore to functionally link eg. nuclear HMTs and cytoplasmic NNMT so there are unlikely to be differences in concentration between nuclear and cytoplasmic SAM levels, and we are not aware of any evidence in the literature to suggest otherwise. Therefore the only way we could look at this would be to investigate mitochondria; however no HMTs are mitochondrially localised so we would not be able to perform this analysis.

Minor concerns:
1) Fig 1E should be placed before the figure which introduces NNMT. The current placement breaks the flow from 1D to 1F.

As suggested, we have swapped panels Fig 1D and 1E to introduce NNMT via the schematic prior to introducing data about NNMT.

**REFERENCES**

Aranda, S., A. Alcaine-Colet, E. Blanco, E. Borràs, C. Caillot, E. Sabidó and L. Di Croce (2019). "Chromatin capture links the metabolic enzyme AHCY to stem cell proliferation." Science Advances **5**(3): eaav2448.

Belužić, L., I. Grbeša, R. Belužić, J. H. Park, H. K. Kong, N. Kopjar, G. Espadas, E. Sabidó, A. Lepur and F. Rokić (2018). "Knock-down of AHCY and depletion of adenosine induces DNA damage and cell cycle arrest." Scientific Reports **8**(1): 14012.

Ceol, C. J., Y. Houvras, J. Jane-Valbuena, S. Bilodeau, D. A. Orlando, V. Battisti, L. Fritsch, W. M. Lin, T. J. Hollmann and F. Ferré (2011). "The histone methyltransferase SETDB1 is recurrently amplified in melanoma and accelerates its onset." Nature **471**(7339): 513-517.

Eckert, M. A., F. Coscia, A. Chryplewicz, J. W. Chang, K. M. Hernandez, S. Pan, S. M. Tienda, D. A. Nahotko, G. Li and I. Blaženović (2019). "Proteomics reveals NNMT as a master metabolic regulator of cancer-associated fibroblasts." Nature **569**(7758): 723-728.

Finkelstein, J. D. (1990). "Methionine metabolism in mammals." The Journal of nutritional biochemistry **1**(5): 228-237.

Grbeša, I., A. Kalo, R. Belužić, L. Kovačević, A. Lepur, F. Rokić, H. Hochberg, I. Kanter, V. Simunović and P. M. Muńoz-Torres (2017). "Mutations in S-adenosylhomocysteine hydrolase (AHCY) affect its nucleocytoplasmic distribution and capability to interact with S-adenosylhomocysteine hydrolase-like 1 protein." European Journal of Cell Biology **96**(6): 579-590.

Greco, C. M., M. Cervantes, J.-M. Fustin, K. Ito, N. Ceglia, M. Samad, J. Shi, K. B. Koronowski, I. Forne and S. Ranjit (2020). "S-adenosyl-l-homocysteine hydrolase links methionine metabolism to the circadian clock and chromatin remodeling." Science advances **6**(51): eabc5629.

Griffin, G. K., J. Wu, A. Iracheta-Vellve, J. C. Patti, J. Hsu, T. Davis, D. Dele-Oni, P. P. Du, A. G. Halawi and J. J. Ishizuka (2021). "Epigenetic silencing by SETDB1 suppresses tumour intrinsic immunogenicity." Nature **595**(7866): 309-314.

Harmankaya, İ., S. Akar, S. Uğraş, A. H. Güler, H. Ezveci, M. Aydoğdu and Ç. Çelik (2021). "Nicotinamide N-methyltransferase overexpression may be associated with poor prognosis in ovarian cancer." Journal of Obstetrics and Gynaecology **41**(2): 248-253.

Hong, S., B. Zhai and P. Pissios (2018). "Nicotinamide N-methyltransferase interacts with enzymes of the methionine cycle and regulates methyl donor metabolism." Biochemistry **57**(40): 5775-5779.

Jiang, Y., C. Xiang, F. Zhong, Y. Zhang, L. Wang, Y. Zhao, J. Wang, C. Ding, L. Jin and F. He (2021). "Histone H3K27 methyltransferase EZH2 and demethylase JMJD3 regulate hepatic stellate cells activation and liver fibrosis." Theranostics **11**(1): 361.

Kim, J., S. J. Hong, E. K. Lim, Y.-S. Yu, S. W. Kim, J. H. Roh, I.-G. Do, J.-W. Joh and D. S. Kim (2009). "Expression of nicotinamide N-methyltransferase in hepatocellular carcinoma is associated with poor prognosis." Journal of Experimental & Clinical Cancer Research **28**(1): 1-9.

Kraus, D., Q. Yang, D. Kong, A. S. Banks, L. Zhang, J. T. Rodgers, E. Pirinen, T. C. Pulinilkunnil, F. Gong and Y.-c. Wang (2014). "Nicotinamide N-methyltransferase knockdown protects against diet-induced obesity." Nature **508**(7495): 258-262.

Lim, B.-H., B.-I. Cho, Y. N. Kim, J. W. Kim, S.-T. Park and C.-W. Lee (2006). "Overexpression of nicotinamide N-methyltransferase in gastric cancer tissues and its potential post-translational modification." Experimental & molecular medicine **38**(5): 455-465.

MacGregor, S., G. W. Montgomery, J. Z. Liu, Z. Z. Zhao, A. K. Henders, M. Stark, H. Schmid, E. A. Holland, D. L. Duffy and M. Zhang (2011). "Genome-wide association study identifies a new melanoma susceptibility locus at 1q21. 3." Nature genetics **43**(11): 1114-1118.

Mosammaparast, N. and Y. Shi (2010). "Reversal of histone methylation: biochemical and molecular mechanisms of histone demethylases." Annual review of biochemistry **79**: 155-179.

Orouji, E., A. Federico, L. Larribère, D. Novak, D. B. Lipka, Y. Assenov, S. Sachindra, L. Hüser, K. Granados and C. Gebhardt (2019). "Histone methyltransferase SETDB1 contributes to melanoma tumorigenesis and serves as a new potential therapeutic target." International journal of cancer **145**(12): 3462-3477.

Ponnaluri, V. C., P.-O. Estève, C. I. Ruse and S. Pradhan (2018). "S-adenosylhomocysteine hydrolase participates in DNA methylation inheritance." Journal of molecular biology **430**(14): 2051-2065.

Radomski, N., C. Kaufmann and C. Dreyer (1999). "Nuclear accumulation of S-adenosylhomocysteine hydrolase in transcriptionally active cells during development of Xenopus laevis." Molecular biology of the cell **10**(12): 4283-4298.

Roeßler, M., W. Rollinger, S. Palme, M.-L. Hagmann, P. Berndt, A. M. Engel, B. Schneidinger, M. Pfeffer, H. Andres and J. Karl (2005). "Identification of nicotinamide N-methyltransferase as a novel serum tumor marker for colorectal cancer." Clinical cancer research **11**(18): 6550-6557.

Sperber, H., J. Mathieu, Y. Wang, A. Ferreccio, J. Hesson, Z. Xu, K. A. Fischer, A. Devi, D. Detraux and H. Gu (2015). "The metabolome regulates the epigenetic landscape during naive-to-primed human embryonic stem cell transition." Nature cell biology **17**(12): 1523-1535.

Tang, S.-W., T.-C. Yang, W.-C. Lin, W.-H. Chang, C.-C. Wang, M.-K. Lai and J.-Y. Lin (2011). "Nicotinamide N-methyltransferase induces cellular invasion through activating matrix metalloproteinase-2 expression in clear cell renal cell carcinoma cells." Carcinogenesis **32**(2): 138-145.

Thul, P. J. and C. Lindskog (2018). "The human protein atlas: a spatial map of the human proteome." Protein Science **27**(1): 233-244.

Tomida, M., I. Mikami, S. Takeuchi, H. Nishimura and H. Akiyama (2009). "Serum levels of nicotinamide N-methyltransferase in patients with lung cancer." Journal of cancer research and clinical oncology **135**(9): 1223-1229.

Ulanovskaya, O. A., A. M. Zuhl and B. F. Cravatt (2013). "NNMT promotes epigenetic remodeling in cancer by creating a metabolic methylation sink." Nature chemical biology **9**(5): 300-306.

Wu, Y., M. Siadaty, M. Berens, G. Hampton and D. Theodorescu (2008). "Overlapping gene expression profiles of cell migration and tumor invasion in human bladder cancer identify metallothionein 1E and nicotinamide N-methyltransferase as novel regulators of cell migration." Oncogene **27**(52): 6679-6689.

Zhou, W., M. Gui, M. Zhu, Z. Long, L. Huang, J. Zhou, L. He and K. Zhong (2014). "Nicotinamide N‑methyltransferase is overexpressed in prostate cancer and correlates with prolonged progression‑free and overall survival times." Oncology Letters **8**(3): 1175-1180.
